# Targeting GPR65 alleviates hepatic inflammation and fibrosis by suppressing the JNK and NF-κB pathways

**DOI:** 10.1186/s40779-023-00494-4

**Published:** 2023-11-25

**Authors:** Kun Zhang, Meng-Xia Zhang, Xiao-Xiang Meng, Jing Zhu, Jia-Jun Wang, Yi-Fan He, Ye-Hua Li, Si-Cong Zhao, Zhe-Min Shi, Li-Na Zheng, Tao Han, Wei Hong

**Affiliations:** 1 Department of Histology and Embryology, School of Basic Medical Sciences, Tianjin Medical University, Tianjin, 300070 China; 2 Department of Hepatology and Gastroenterology, Tianjin Union Medical Center, Tianjin Medical University, Tianjin Union Medical Center affiliated to Nankai University, Tianjin, 300000 China

**Keywords:** GPR65, Hepatic fibrosis, Hepatic macrophages, Inflammation, JNK, NF-κB

## Abstract

**Background:**

G-protein coupled receptors (GPCRs) are recognized as attractive targets for drug therapy. However, it remains poorly understood how GPCRs, except for a few chemokine receptors, regulate the progression of liver fibrosis. Here, we aimed to reveal the role of GPR65, a proton-sensing receptor, in liver fibrosis and to elucidate the underlying mechanism.

**Methods:**

The expression level of GPR65 was evaluated in both human and mouse fibrotic livers. Furthermore, *Gpr65*-deficient mice were treated with either bile duct ligation (BDL) for 21 d or carbon tetrachloride (CCl_4_) for 8 weeks to investigate the role of GPR65 in liver fibrosis. A combination of experimental approaches, including Western blotting, quantitative real-time reverse transcription‑polymerase chain reaction (qRT-PCR), and enzyme-linked immunosorbent assay (ELISA), confocal microscopy and rescue studies, were used to explore the underlying mechanisms of GPR65’s action in liver fibrosis. Additionally, the therapeutic potential of GPR65 inhibitor in the development of liver fibrosis was investigated.

**Results:**

We found that hepatic macrophages (HMs)-enriched GPR65 was upregulated in both human and mouse fibrotic livers. Moreover, knockout of *Gpr65* significantly alleviated BDL- and CCl_4_-induced liver inflammation, injury and fibrosis in vivo, and mouse bone marrow transplantation (BMT) experiments further demonstrated that the protective effect of *Gpr65* knockout is primarily mediated by bone marrow-derived macrophages (BMMs). Additionally, in vitro data demonstrated that *Gpr65* silencing and GPR65 antagonist inhibited, while GPR65 overexpression and application of GPR65 endogenous and exogenous agonists enhanced the expression and release of tumor necrosis factor-α (TNF-α), interleukin-6 (IL-6) and transforming growth factor-β (TGF-β), all of which subsequently promoted the activation of hepatic stellate cells (HSCs) and the damage of hepatocytes (HCs). Mechanistically, GPR65 overexpression, the acidic pH and GPR65 exogenous agonist induced up-regulation of TNF-α and IL-6 via the Gαq-Ca^2+^-JNK/NF-κB pathways, while promoted the expression of TGF-β through the Gαq-Ca^2+^-MLK3-MKK7-JNK pathway. Notably, pharmacological GPR65 inhibition retarded the development of inflammation, HCs injury and fibrosis in vivo.

**Conclusions:**

GPR65 is a major regulator that modulates the progression of liver fibrosis. Thus, targeting GPR65 could be an effective therapeutic strategy for the prevention of liver fibrosis.

**Supplementary Information:**

The online version contains supplementary material available at 10.1186/s40779-023-00494-4.

## Background

Hepatic fibrosis has emerged as a major chronic liver pathological process, characterized by the excessive deposition of extracellular matrix (ECM), the development of inflammation and hepatic injury [1]. This process is caused by a persistent hepatic damage and injury repair response induced by various etiologies including biliary obstruction, hepatitis B/C virus infection and alcohol abuse [1–3]. If the damage persists, the fibrotic process will result in liver failure and cirrhosis, causing more than 1.32 million deaths annually globally [4]. Regrettably, no effective drug for the treatment of hepatic fibrosis has been approved by the Food and Drug Administration so far. Therefore, a more detailed understanding of the pathophysiological mechanism of fibrosis is needed to develop promising drug targets and corresponding therapeutic molecules that prevent the progression of hepatic fibrosis.

G-protein coupled receptors (GPCRs), which are the largest superfamily of transmembrane proteins encoded by the human genome, mediate the majority of cellular responses to external stimuli, including light, odors, ions, hormones, and growth factors [5, 6]. Although GPCRs as the most productive drug targets, are targeted by one-third of the drugs in clinical use, few GPCRs are reported to be involved in hepatic fibrosis except for a few chemokine receptors [6, 7]. GPR65 (also known as TDAG8) was initially identified as orphaned GPCR in apoptotic thymocytes [8]. Currently, it has been accepted that GPR65, along with GPR4, GPR68 (also known as OGR1) and GPR132 (also known as G2A), forms a group of proton-sensing GPCRs [9]. The signaling networks downstream of GPR65 have been implicated in many pathophysiological processes including tumor, immune-related diseases and inflammation through Gαs/cyclic AMP/protein kinase A/cAMP-responsive-element-binding protein (Gαs/cAMP/PKA/CREB) and Gαq/phospholipase C beta/protein kinase C (Gαq/PLC-β/PKC) pathways in response to extracellular stimuli [10–12]. However, GPR65 may have both protective and detrimental roles [10, 11, 13–15], for example, it is downregulated in hematological malignancies and functions as a tumor suppressor to promote apoptosis of murine lymphoma cells [10]. Also, GPR65 partly mediates the inhibition of extracellular acidification-induced pro-inflammatory cytokines via the Gαs/PKA pathway [11]. On the other hand, GPR65 functions as an oncogenic GPCR as it is increased in a series of human cancer tissues [13]. Similarly, blocking GPR65 expression and function is associated with reduced pro-inflammatory cytokines releasing, microglial activation and eosinophil viability [12, 14]. Hepatic macrophages (HMs) are heterogeneous cell populations, consisting of liver-resident macrophages, traditionally termed Kupffer cells (KCs), and monocyte-derived macrophages that are recruited from the circulation upon acute or chronic liver injury [16, 17]. Additionally, macrophages can be broadly classified into two main subsets: classically activated macrophages (M1), characterized by a pro-inflammatory phenotype, and alternatively activated macrophages (M2), which are associated with anti-inflammatory and tissue repair functions [16, 17]. Nevertheless, the function of GPR65 in HMs in the process of inflammation and fibrosis is still unclear.

In the present study, we identified that HMs-enriched GPR65 was increased in both human and mouse fibrotic livers and HMs from fibrotic livers. We conducted a series of experiments, such as gain- and loss-of-function assays, the application of the specific antagonist and agonist of GPR65, and combined with *Gpr65*-deficient mice as well as mouse bone marrow transplantation (BMT) experiments to investigate the function and the underlying mechanisms of GPR65 in HMs, hepatic stellate cells (HSCs) and hepatocytes (HCs), and the intercellular network during liver fibrogenesis, thus providing a new therapeutic strategy for the prevention of liver fibrosis.

## Methods

### Animals in vivo study

All of the animal works were conducted according to the approved guidelines and approved by the Animal Care and Use Committee of Tianjin Medical University (TMUaMEC2018025). Male 6–8 weeks wild-type (WT) mice and *Gpr65* knockout (GPR65-KO) mice, which were generated using CRISPR-Cas9 technology (Cyagen Biosciences, Suzhou, China) on a C57BL/6N background were used in the carbon tetrachloride (CCl_4_)- or bile duct ligation (BDL)-induced hepatic fibrosis models, as previously described [18, 19]. In brief, the gRNAs sequences targeting *Gpr65* were gRNA1: GGAGATTGGTCGGTGCAAATGGG; gRNA2: ATAACCCCTAAGAAGCACGCGGG. The primers used for identification were as follows: upstream primer 1: 5′-AATGTGACTTTTGAAATGCC-3′; upstream primer 2: 5′-ATAGACTAAGAGGTGGAGGC-3′; downstream primer: 5′-AACTAGGCAGGGTCAATTCC-3′. Sequence primer for the product: 5′-AATGTGACTTTTGAAATGCC-3′. WT and GPR65-KO mice came from the same heterozygous mice and hosted under pathogen-free conditions. WT or GPR65-KO mice were separated into 2 groups randomly: WT (*n* = 5) and WT + CCl_4_/BDL (*n* = 10) or GPR65-KO (*n* = 5) and GPR65-KO + CCl_4_/BDL (*n* = 10). Thirty 6–8 weeks-old BALB/c male mice were purchased from Beijing HFK Bioscience (Beijing, China) and separated into 4 groups randomly: Ctrl (*n* = 5), Ctrl + CCl_4_ (*n* = 10), ZINC62678696 (the inhibitor of GPR65; *n* = 5), ZINC62678696 + CCl_4_ (*n* = 10). The GPR65 inhibitor (10 mg/kg) was administered 4 weeks after the first CCl_4_ injection every two days. For CCl_4_-induced hepatic fibrosis mouse model, mice were treated with 0.25 ml/kg CCl_4_ dissolved in olive oil or olive oil twice a week via intraperitoneal injection for 6–8 weeks. For BDL-induced hepatic fibrosis mouse model, mice were administered BDL operation or sham operation and were sacrificed under anesthesia with 3% sodium pentobarbital (45 mg/kg, ip) after 21 d. For mouse BMT experiments, the male WT recipient mice at 8 weeks of age were given lethal irradiation with 9 Gy (RS2000PRO-225 X-RAY, 4.5 Gy twice between 4 h) and then intravenous injection of 1 × 10^7^ bone marrow cells (100–150 μl) harvested from WT or GPR65-KO donor mice (*n* = 8). Four weeks after irradiation and BMT, the chimeric mice were treated with 0.25 ml/kg CCl_4_ dissolved in olive oil thrice a week via intraperitoneal injection for 6 weeks. Serums and liver specimens were collected for further analyses.

### Patient samples

Six normal livers and 28 human fibrotic livers were obtained from Tianjin Third Central Hospital (Tianjin, China) as previously reported [18, 20, 21]. All subjects were of the same ethnicity. Clinical and pathological characteristics including age, gender, alanine aminotransferase (ALT), aspartate aminotransferase (AST), γ-glutamyl transpeptidase (GGT) and etiologies were recorded and summarized in Additional file 1: Table S1. For histological scoring of liver fibrosis, we stained paraffin-embedded 5 μm liver sections with Sirius red staining, HE staining and Masson’s trichrome staining. Hepatic fibrosis was scored (stages F0–F4) according to the METAVIR fibrosis stage system by three hepatopathologists blinded to the study protocol and stratified as normal liver (F0), mild fibrosis (F1–F2) or advanced fibrosis (F3–F4). The research methods were consistent with the standards in the Helsinki Declaration. Written informed consents were acquired from each patient and the study has been approved by the local Ethical Committee of Tianjin Third Central Hospital (SZX-IRB2020-005-02).

### Culture and treatments of primary cells and cell lines

Mouse primary HMs, HSCs, HCs and liver sinusoidal endothelial cells (LSECs) were isolated by in situ perfusion digestion as previously reported [17–21]. HMs, HCs, and LSECs were isolated from 8 to 10 weeks-old male mice, while HSCs was isolated from male BALB/c mice at 40 weeks of age. In brief, mice were anaesthetized via sodium pentobarbital (3%, ip). Sequential infusion of ethylene glycol bis(2-aminoethyl ether) tetraacetic acid (EGTA, 2 min), 0.05% pronase E (10 min, Roche, Germany, Cat# 11459643001), 0.025% collagenase type IV (10 min, Sigma-Aldrich, USA, Cat# C5138) were performed via the portal vein. Liver tissues were then digested in GBSS/B containing 0.033% pronase E (Roche, Germany, Cat# 11459643001), 0.04% collagenase type IV (Sigma-Aldrich, USA, Cat# C5138) and 1% DNase 1 (Roche, Germany, Cat# 10104159001) for 20 min at 37 °C in shaker bath. The digested cell suspension was then filtered via a 70 mm cell filter and centrifuged at 50 *g* for 6 min three times to remove HCs. HMs, HSCs and LSECs were isolated from the suspension using 8.2%, 12% and 18% Histodenz solution (Sigma-Aldrich, USA, Cat# D2158) at 1450 *g* for 22 min. Selective adhesion or magnetic-activated cell sorting (MACS)-based positive selection using an F4/80 antibody (Miltenyi Biotec, Germany, Cat# 130-110-443) and an CD146 antibody (Miltenyi Biotec, Germany, Cat# 130-092-007) were used to purify HMs and LSECs respectively. The purity of HSCs was detected by perinuclear lipid droplets and morphology when the primary HSCs were isolated, and we performed α-smooth muscle actin (α-SMA) staining in primary HSCs at day 7. Trypan blue exclusion assay was used to measure the cell viability. All primary cells were maintained in Dulbecco's Modified Eagle’s Medium (DMEM, Gibco, USA, Cat# C11995500BT) containing 10% fetal bovine serum (FBS, Gibco, USA, Cat# 16000-044), and 1% penicillin/streptomycin (Solarbio, China, Cat# P1400). In addition, primary HCs and LSECs were seeded in plates coated with type I collagen (Sigma-Aldrich, USA, Cat# C7661).

The murine immortalized macrophages RAW264.7, human umbilical vein endothelial cells (HUVECs) and HEK-293T cells (ATCC, USA) were cultured in DMEM containing 10% FBS, 1% penicillin/streptomycin (Solarbio, China, Cat# P1400). Human hepatic stellate cell line LX-2 cells (Sigma-Aldrich, USA) were maintained in DMEM (Gibco, USA, Cat# C11995500BT) supplemented with 10% heat-inactivated FBS (Gibco, USA, Cat# 26140-079) and 1% penicillin/streptomycin. Bone marrow-derived macrophages (BMMs) were isolated from the femur and tibia of WT or GPR65-KO mice (8–9 weeks) and cultured in minimum essential medium alpha (α-MEM, Gibco, USA, Cat# C11095500BT) containing 10% FBS, 1% penicillin/streptomycin (Solarbio, China, Cat# P1400) and 10 ng/ml murine macrophage colony stimulating factor (M-CSF, PeproTech, USA, Cat# 300-25) for 6 d. The non-tumorigenic mouse hepatocyte cell line AML12 cells (National Collection of Authenticated Cell Cultures, China) were cultured in DMEM/F-12 (Gibco, USA, Cat# C11330500BT) containing 10% FBS, 40 ng/ml dexamethasone (Sigma-Aldrich, USA, Cat# D4902), 1 × insulin–transferrin–sodium selenite media supplement (ITS, Sigma-Aldrich, USA, Cat# I3146), 1% penicillin/streptomycin (Solarbio, China, Cat# P1400). Cells were treated with the GPR65 agonist BTB09089 (Maybridge, Belgium, Cat# BTB09089; or Otava, Ukraine, Cat# 14900668; 30 μmol/L), GPR65 inhibitor ZINC62678696 (Enamine, USA, Cat# EN300-261362; or Molport, Latvia, Cat# molport-019-671-510; 30 μmol/L), BAY 11-7082 (MCE, USA, Cat# HY-13453; 5 μmol/L), ammonium pyrrolidine dithiocarbamate (APDC, MCE, USA, Cat# HY-18738; 20 μmol/L), SP600125 (MCE, USA, Cat# HY-12041; 10 μmol/L), JNK inhibitor XVI (JNK-IN-8, Selleck, USA, Cat# S4901; 5 μmol/L), YM-254890 (Focus Biomolecules, USA, Cat# 10-1590; 1 μmol/L), BAPTA (MCE, USA, Cat# HY-100168, 10 μmol/L) or Compound C (MCE, USA, Cat# HY-13418A; 10 μmol/L). For acidic exposure, DMEM or α-MEM with 10% FBS was prepared by using biologic buffer [either 4-(2-hydroxyethyl)-1-piperazineethanesulfonic acid (HEPES), HCl or NaOH for pH 6.6, pH 8.2 and pH 7.2, respectively]. Cells were treated with the appropriate pH and the pH of the culture media was regularly monitored using an acid–base indicator (e.g., phenol red) and adjusted for maintenance. For co-culture experiment, HMs and RAW264.7 cells were treated as described in individual experiment. The centrifuged cell supernatant was filtered through 0.22 μm filtration and was finally collected as the conditioned medium (CM). The CM with 20 μg/ml transforming growth factor-β (TGF-β) neutralization antibody 1D11 (Thermo Fisher Scientific, USA, Cat# 16-9243-85; RRID: AB_2573124) or mouse IgG, was used to treat primary HSCs.

### Quantitative real-time reverse transcription-polymerase chain reaction (qRT-PCR)

RNA isolation and qRT-PCR were performed as previously reported [17–21]. The gene expression was normalized to housekeeping gene *GAPDH*, and fold change was determined utilizing the 2^−∆∆Ct^ method. For cell-by-cell qPCR analysis, the data were analyzed based on GAPDH calibration and compared with normal HMs. The primer sequences for qRT-PCR are provided in Additional file 1: Table S2.

### Western blotting

Protein isolation and Western blotting were performed as previously reported [17–21]. Antibodies used for Western blotting included collagen I (rabbit polyclonal, Abcam, USA, ab34710; RRID: AB_731684; 1:1000; rabbit polyclonal, ABclonal, China, A1352; RRID:AB_2760381; 1:1000; for tissues; rabbit polyclonal, Novus, USA, NB600-408 for cells; RRID: AB_10000511; 1:2000), α-SMA (rabbit polyclonal, Abcam, USA, ab5694 for tissues; RRID: AB_2223021; 1:1000; rabbit polyclonal, ABclonal, USA, A17910 for cells; RRID: AB_2861755; 1:1000), tissue metalloproteinase inhibitor 1 (TIMP1, mouse monoclonal, Santa Cruz, USA, sc-21734; RRID: AB_628359; 1:1000), matrix metalloproteinase 2 (MMP2, rabbit monoclonal, Abcam, USA, ab92536; RRID: AB_10561597; 1:1000), vimentin (rabbit monoclonal, CST, USA, #5741; RRID: AB_10695459; 1:1000), Bcl-2-associated X protein (BAX, rabbit polyclonal, Abcam, USA, ab32503; RRID: AB_725631; 1:1000), cleaved caspase-3 (rabbit monoclonal, CST, USA, #9661; RRID: AB_2341188; 1:1000), caspase-3 (rabbit polyclonal, CST, USA, #9662; RRID: AB_331439; 1:1000), proliferating cell nuclear antigen (PCNA, rabbit monoclonal, CST, USA, #13110; RRID: AB_263697; 1:1000), monocyte chemoattractant protein 1 (MCP-1, rabbit polyclonal, CST, USA, #2029; RRID: AB_1264199; 1:1000), CD11b (rabbit monoclonal, Abcam, USA, ab133357; RRID: AB_2650514; 1:1000), interleukin-1β (IL-1β, mouse monoclonal, CST, USA, #12242; RRID: AB_2715503; 1:1000), lymphocyte antigen 6 complex, locus C (LY6C, rat monoclonal, Novus, USA, NBP2-00441; RRID: AB_2909793; 1:1000), tumor necrosis factor-α (TNF-α, mouse monoclonal, Santa Cruz, USA, sc-52746; RRID: AB_630341; 1:1000), p-IĸB kinase (p-IKK, rabbit monoclonal, CST, USA, #2697; RRID: AB_2079382; 1:1000), p-p65 (rabbit monoclonal, CST, USA, #3033; RRID: AB_331284; 1:1000), p-inhibitor of ĸB α (p-IĸBα, mouse monoclonal, CST, USA, #9246; RRID: AB_2267145; 1:1000), IKK (rabbit monoclonal, Abcam, USA, ab178870; RRID: AB_2924430; 1:1000), p65 (mouse monoclonal, Santa Cruz, USA, sc-8008; RRID: AB_62801; 1:200), IĸBα (mouse monoclonal, CST, USA, #4814; RRID: AB_390781; 1:1000), p-mixed lineage kinase 3 (p-MLK3, rabbit polyclonal, Affinity Biosciences, USA, # AF8079; RRID: AB_2840142; 1:1000), p-mitogen-activated protein kinase kinase 4 (p-MKK4, rabbit polyclonal, CST, USA, #9156; RRID: AB_2297420; 1:1000), p-MKK7 (rabbit recombinant, Proteintech, USA, #80357-1-RR; RRID: AB_2918891; 1:2000), p-JNK (rabbit monoclonal, CST, USA, #4668; RRID: AB_823588; 1:1000), p-p44/42 (rabbit monoclonal, CST, USA, #4370; RRID: AB_2315112; 1:1000), p-p38 (rabbit monoclonal, CST, USA, #4511; RRID: AB_2139682; 1:1000), JNK (rabbit polyclonal, CST, USA, #9252; RRID: AB_2250373; 1:1000), p44/42 (rabbit monoclonal, CST, USA, #4695; RRID: AB_390779; 1:1000), p38 (rabbit monoclonal, CST, USA, #8690; RRID: AB_10999090; 1:1000), p-glycogen synthase kinase-3β (p-GSK-3β, rabbit monoclonal, CST, USA, #5558; RRID: AB_10013750; 1:1000), p-protein kinase B (p-Akt, Ser473, rabbit monoclonal, CST, USA, #4060; RRID: AB_231504; 1:1000), p-pyruvate dehydrogenase kinase 1 (p-PDK1, Ser241, rabbit monoclonal, CST, USA, #3438; RRID: AB_2161134; 1:1000), p-phosphatase and tensin homologue (p-PTEN, Ser380, rabbit polyclonal, CST, USA, #9551; RRID: AB_331407; 1:1000), p-c-Raf (Ser259, rabbit polyclonal, CST, USA, #9421; RRID: AB_330759; 1:1000), Akt (pan) (rabbit monoclonal, CST, USA, #4691; RRID: AB_915783; 1:1000), GAPDH (mouse monoclonal, Abcam, USA, ab8245; RRID: AB_2107448; 1:8000). GAPDH was used as an internal control.

### Histology and immunohistochemistry (IHC)

Formalin-fixed paraffin-embedded slides were prepared for histopathological analysis and IHC as previously reported [17–21]. In brief, 5 μm sections were stained with Sirius red staining, HE staining and Masson’s trichrome staining. Sirius red staining was conducted using 0.1% Direct Red 80 (Sigma, USA, Cat# 365548) in 1.3% picric acid solution (Sigma, USA, Cat# 239801) for 45 min, before dehydrated with ethanol and xylene. For Masson’s trichrome staining, slides were dewaxed, rehydrated, stained with hematoxylin (Thermo Fisher, USA, Cat# SH26500D) for 5 min. Then stained with fuchsin solution for 10 s, phosphomolybdic acid solution for 2 min and aniline blue solution for 1 min followed by washing with weak acid working solution for 1 min. For IHC analysis, sections of three mice for each group were analyzed. Antibodies used for immunohistochemistry included GPR65 (Novus, USA, NBP2-24487 for human; 1:100; AVIVA Systems Biology, USA, for mouse, OABF01813; 1:200), collagen I (Novus, USA, NB600-408; 1:100), α-SMA (Abcam, USA, ab5694; 1:200), TGF-β (Abcam, USA, ab66043; 1:100), BAX (Abcam, USA, ab32503; 1:250), cleaved caspase-3 (CST, USA, #9661; 1:200), PCNA (CST, USA, #13110; 1:1000), Ki67(Abcam, USA, ab16667; 1:200), F4/80 (Invitrogen, USA, 14-4801-81; 1:50), IL-1β (CST, USA, #12242; 1:100), and LY6C (Novus, USA, NBP2-00441; 1:200). In addition, control IgG staining was included as a negative control.

### RNA-seq

Briefly, samples (12 liver tissues from WT, WT + BDL, GPR65-KO and GPR65-KO + BDL respectively, *n* = 3) were collected and lysed with TRIzol reagent. RNA was qualified by Agilent 2100 bioanalyzer (Thermo, USA) and subsequently screened on BGIseq500 platform (BGI-Shenzhen, China). The threshold was fold change > 2 and *P-*adj < 0.05. The sequencing data have been deposited in NCBI GEO database: GSE182235 (https://www.ncbi.nlm.nih.gov/geo/query/acc.cgi?acc=GSE182235).

### Statistical analysis

Data were expressed as means ± SEM with at least three independent experiments, except where noted. All statistical analyses were conducted using GraphPad 9. Statistical analysis was conducted using either one-way analysis of variance (more than two groups) or Student’s *t*-test (two group comparison), and *P* < 0.05 indicates a significant difference.

Details on other methods are shown in the Additional file 1.

## Results

### GPR65 is increased in human and mouse hepatic fibrosis

**Fig. 1:**
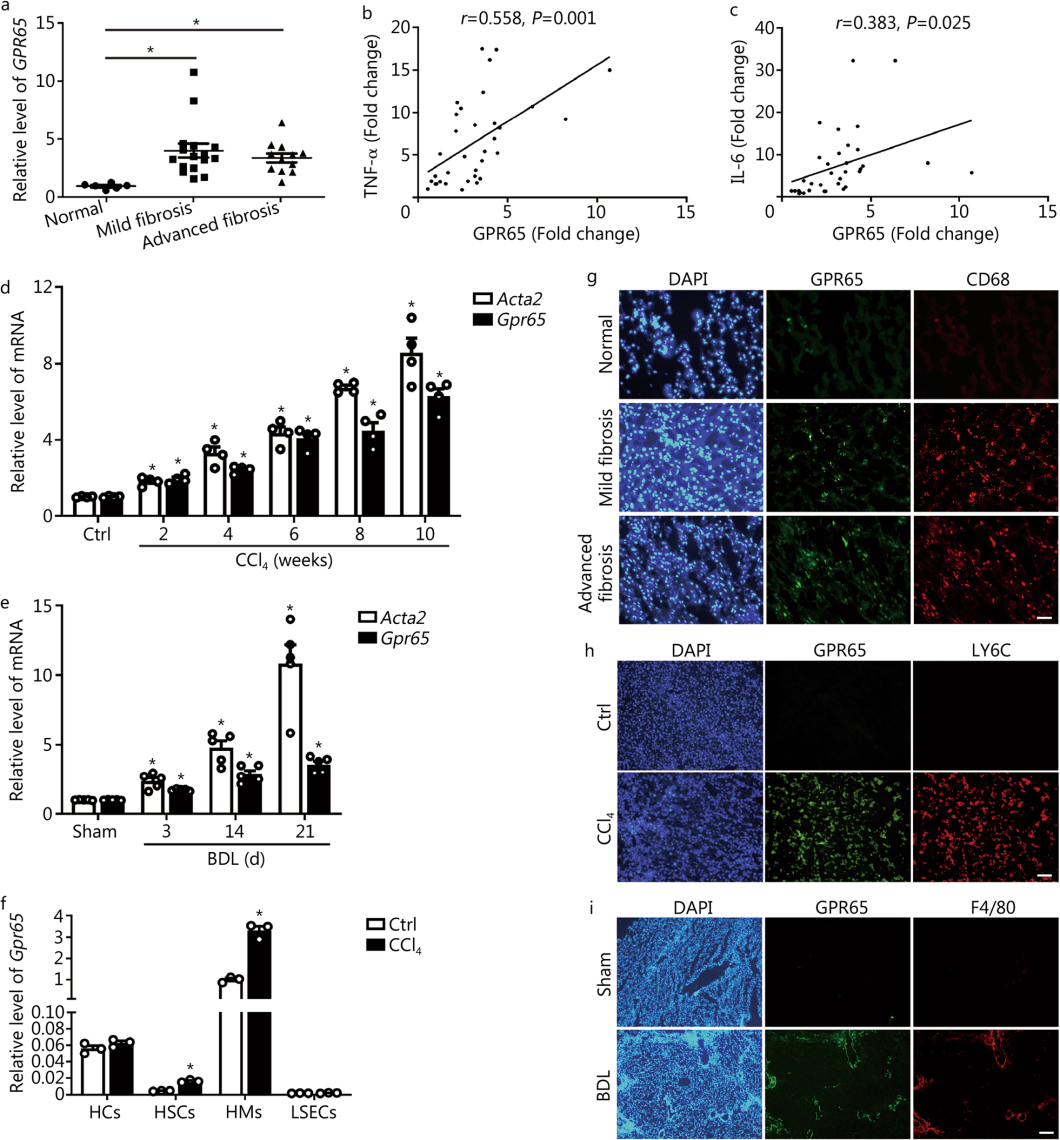
GPR65 is overexpressed in mouse and human hepatic fibrosis. **a** qRT-PCR was used to assess the mRNA level of *Gpr65* in liver tissues of human normal controls (*n* = 6), mild fibrosis (*n* = 16) and advanced fibrosis (*n* = 12). The correlations of GPR65, TNF-α (**b**) and IL-6 (**c**) were assessed using Pearson correlation analysis, *n* = 34. **d** qRT-PCR was used to assess the expression of *Acta2 (α-SMA)* and *Gpr65* in livers from mice with CCl_4_ treatment for different time points (*n* = 4). **e** qRT-PCR was used to assess the expression of *Acta2* and *Gpr65* in livers from mice treated with BDL for different time points (*n* = 5). **f** qRT-PCR was used to assess the expression of *Gpr65* in HCs, HSCs, HMs and LSECs that were isolated from livers of mice without or with CCl_4_ treatment for 8 weeks (*n* = 3). **g** Representative IHC-frozen for co-staining GPR65 with macrophage marker CD68 in the liver of human normal controls, mild fibrosis and advanced fibrosis. Scale bar = 50 μm. **h** Representative IHC-frozen for co-staining GPR65 with macrophage marker LY6C in the livers of mice without or with CCl_4_ treatment for 8 weeks. Scale bar = 50 μm. **i** Representative IHC-frozen for co-staining GPR65 with macrophage marker F4/80 in the livers of mice treated without or with BDL for 21 d. Scale bar = 125 μm. ^*^P < 0.05 vs. Normal or Ctrl or Sham. BDL bile duct ligation, CCl_4_ carbon tetrachloride, Ctrl control, HC hepatocyte, HM hepatic macrophage, HSC hepatic stellate cell, IHC immunohistochemistry, LSEC liver sinusoidal endothelial cell, qRT-PCR quantitative real-time reverse transcription-polymerase chain reaction, TNF-α tumor necrosis factor-α, IL-6 interleukin-6, LY6C lymphocyte antigen 6 complex

To clarify the key GPCRs involved in hepatic fibrosis, we intersected the GPCRs gene set with our previous microarray data which were conducted in mouse normal and fibrotic liver tissues (GSE80601 and GSE89147) (Additional file 1: Fig. S1a–d). Five GPCRs (*C3ar1*, *Ccr2*, *Gpr65*, *P2ry6* and *P2ry14*) which are increased in fibrotic livers more than twofold have been identified (Additional file 1: Fig. S1e). Of note, CCR2 and C3AR1 have been reported to be related with hepatic inflammation and fibrosis [7, 22]. In the present study, we focused our attention on the role and mechanism of proton-sensing receptor GPR65 in hepatic fibrosis. We first examined GPR65 expression in the livers of human subjects without fibrosis, with mild or advanced fibrosis as diagnosed clinically and pathologically (Additional file 1: Fig. S2a). The hepatic level of GPR65 was markedly upregulated in the livers of subjects with fibrosis than it was in individuals with normal livers (Fig. 1a; Additional file 1: Fig. S2a). Moreover, GPR65 was correlated with the expression of TNF-α, IL-6 rather than collagen type I alpha 1 (COL1α1) and actin alpha 2, smooth muscle (ACTA2/α-SMA) (Fig. 1b, c; Additional file 1: Fig. S2b, c). In line with our observation in human, GPR65 expression was considerably upregulated in the liver tissues of CCl_4_- and BDL-treated mice (Fig. 1d, e; Additional file 1: Fig. S2d). Moreover, HCs, HSCs, HMs and LSECs from olive oil-treated and CCl_4_-treated mice were isolated, and we found *Gpr65* was highly expressed in primary HMs, lowly expressed in HCs and minimally expressed in HSCs and LSECs (Fig. 1f). IHC-frozen for co-staining GPR65 with macrophage markers (CD68 for human, LY6C or F4/80 for mouse) and HSC marker α-SMA in normal and fibrotic livers revealed that GPR65 was mainly expressed in HMs (Fig. 1g–i; Additional file 1: Fig. S2e). Consistently, the expression of *Gpr65* in AML12, LX-2, RAW264.7, BMMs and HUVECs further confirmed that GPR65 is a macrophage-enriched GPCR (Additional file 1: Fig. S2f). In addition, the expression of *Gpr65* was increased in primary HMs isolated from mice treated with CCl_4_ for 1, 4 and 8 weeks, in line with the trend observed in *Il1β* and *Ly6c* (Additional file 1: Fig. S2g). However, our data revealed that TGF-β, but not LPS, IFN-γ, IL-4 and IL-10, promoted the expression of *Gpr65* in HMs (Additional file 1: Fig. S2h, i). Taken together, our results demonstrate that HMs-enriched GPR65 is upregulated in both human and mouse fibrotic livers, suggesting a role of GPR65 in the development of hepatic fibrosis.

### *Gpr65* deficiency alleviates BDL-induced hepatic fibrosis

**Fig. 2:**
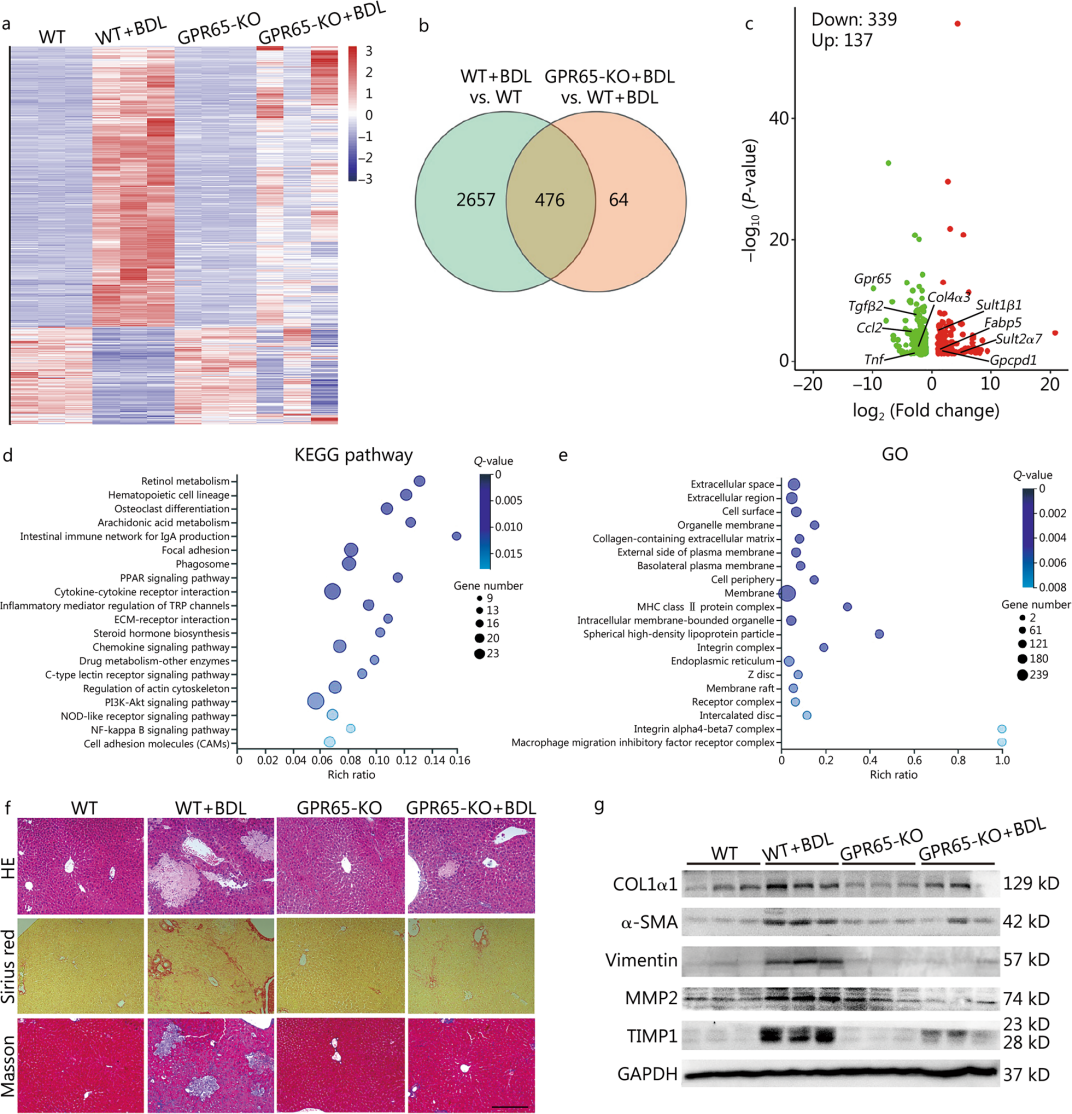
*Gpr65* deficiency alleviates hepatic fibrosis induced by BDL. **a** WT and GPR65-KO mice were divided into 4 groups: WT, WT + BDL, GPR65-KO and GPR65-KO + BDL. The significantly differentially expressed mRNAs were displayed by hierarchical cluster analysis: bright red, up-regulation; bright blue, down-regulation.* n* = 3/group. The Venn diagram (**b**), volcano map analysis (**c**), KEGG (**d**) and GO (**e**) analysis of differentially expressed mRNAs in WT mice treated with BDL, compared to WT mice, and GPR65-KO mice treated with BDL, compared to WT mice treated with BDL. **f** Hepatic fibrosis was evaluated by HE staining, Sirius red staining and Masson’s trichrome staining. Scale bar = 400 μm. **g** Western blotting was used to determine the protein level of COL1α1, α-SMA, vimentin, MMP2 and TIMP1. BDL bile duct ligation, GO Gene Ontology, KEGG Kyoto Encyclopedia of Genes and Genomes, KO knockout, PPAR peroxisome proliferator activated receptor, TRP transient receptor potential, ECM extracellular matrix, NOD nucleotide oligomerization domain, MHC major histocompatibility complex, COL1α1 collagen type I alpha 1, α-SMA α-smooth muscle actin, MMP2 matrix metalloproteinase 2, TIMP1 tissue inhibitor of metalloproteinases 1

To investigate the role of GPR65 in hepatic fibrosis in vivo, we generated GPR65-KO mice and subjected them to sham operation or BDL. *Gpr65* was successfully knocked out in livers of GPR65-KO mice compared to those of WT mice demonstrated by qRT-PCR and DNA sequencing, and BDL did not change the expression of *Gpr65* in the livers of GPR65-KO mice (Additional file 1: Fig. S3a). After 21 d of BDL, we performed RNA-seq to explore the effect of *Gpr65* deficiency on BDL-induced hepatic fibrosis (Fig. 2a). Firstly, our data confirmed that loss of *Gpr65* did not alter liver homeostasis, as demonstrated by changed expression of only four genes (*Gpr65*, *Rnf186*, *Acnat2* and *Creld2*) in the livers of GPR65-KO mice (Fig. 2a). Secondly, 2321 mRNAs were increased and 812 mRNAs were decreased in the BDL-treated WT mice, and the Kyoto Encyclopedia of Genes and Genomes (KEGG) pathway and Gene Ontology (GO) analysis demonstrated that BDL affected a list of genes related with cytokine-cytokine receptor interaction, ECM, ECM-receptor interaction, drug metabolism, fatty acid degradation, retinol metabolism, cell adhesion molecules, PI3K-Akt signaling and arachidonic acid metabolism (Additional file 1: Fig. S3b–d). Thirdly, only 592 mRNAs were increased and 104 mRNAs were decreased in the BDL-treated GPR65-KO mice, compared with GPR65-KO mice. Moreover, 182 mRNAs were increased and 358 mRNAs were decreased in the BDL-treated GPR65-KO mice, compared with the BDL-treated WT mice. Among these, 476 (137 mRNAs were increased and 339 mRNAs were decreased) out of 540 genes were also dysregulated in the BDL-treated WT mice (Fig. 2b, c). In addition, the KEGG pathway and GO analysis demonstrated that loss of *Gpr65* affected a series of genes related with retinol metabolism, arachidonic acid metabolism, cytokine-cytokine receptor interaction, ECM-receptor interaction, drug metabolism, PI3K-Akt signaling, NF-κB signaling and MAPK signaling, suggesting that *Gpr65* deficiency regulated BDL-induced hepatic metabolic dysfunction, inflammation and fibrosis (Fig. 2d, e). To confirm this, we performed HE staining, Sirius red staining, Masson’s trichrome staining and IHC for COL1α1 and α-SMA, and the results revealed that loss of *Gpr65* significantly alleviated BDL-induced hepatic fibrosis (Fig. 2f; Additional file 1: Fig. S3e). Western blotting demonstrated that *Gpr65* deficiency obviously decreased BDL-induced up-regulation of COL1α1, α-SMA, vimentin, MMP2 and TIMP1 (Fig. 2g). Consistently, qRT-PCR analysis showed that deficiency of *Gpr65* noticeably alleviated BDL-induced up-regulation of pro-fibrotic genes including *Acta2*, *Col1α1*, *Col3α1*, *Col12α1*, *Mmp2*, *Tgfβ1* and *Pdgfβ* (Additional file 1: Fig. S3f). The hepatic hydroxyproline content in BDL-treated GPR65-KO mice was also markedly decreased compared to BDL-treated WT mice (Additional file 1: Fig. S3g), suggesting that loss of *Gpr65* significantly alleviated BDL-induced hepatic fibrosis. Furthermore, BDL-treated GPR65-KO mice exhibited significantly reduced hepatic injury and improved liver function than BDL-treated WT mice, as evidenced by HE staining, serum AST and ALT level, IHC and Western blotting for TGF-β1, BAX and cleaved caspase-3, and the mRNA level of genes involved in apoptosis (*Bad* and *Bax*) and drug/lipid/retinol metabolism (*Acnat2*, *Dhrs9*, *Nox2*, *Gpcpd1*, *Gsta2*, *Ugt2β1* and *Sult1β1*) in the livers (Fig. 2f; Additional file 1: Fig. S4a–f). However, the expression of proliferation-related genes, *Ki67* and *Pcna*, was not changed in GPR65-KO mice treated with BDL, compared with WT mice treated with BDL, suggesting that GPR65 may not affect proliferation during liver fibrogenesis (Additional file 1: Fig. S4c, d, f). Additionally, IHC, Western blotting and qRT-PCR results revealed that GPR65-KO mice treated with BDL also exhibited suppressed expression of pro-inflammatory genes (*Ly6c*, *Adgre1*, *Il1β*, *Il6*, *Tnfα*, *Ccl2* and *Ccr2*) (Additional file 1: Fig. S4c, f, g). All these results demonstrate that *Gpr65* depletion ameliorates BDL-induced hepatic inflammation, injury and fibrosis in vivo.

### ***Gpr65*** deficiency alleviates CCl_4_-induced hepatic fibrosis

**Fig. 3:**
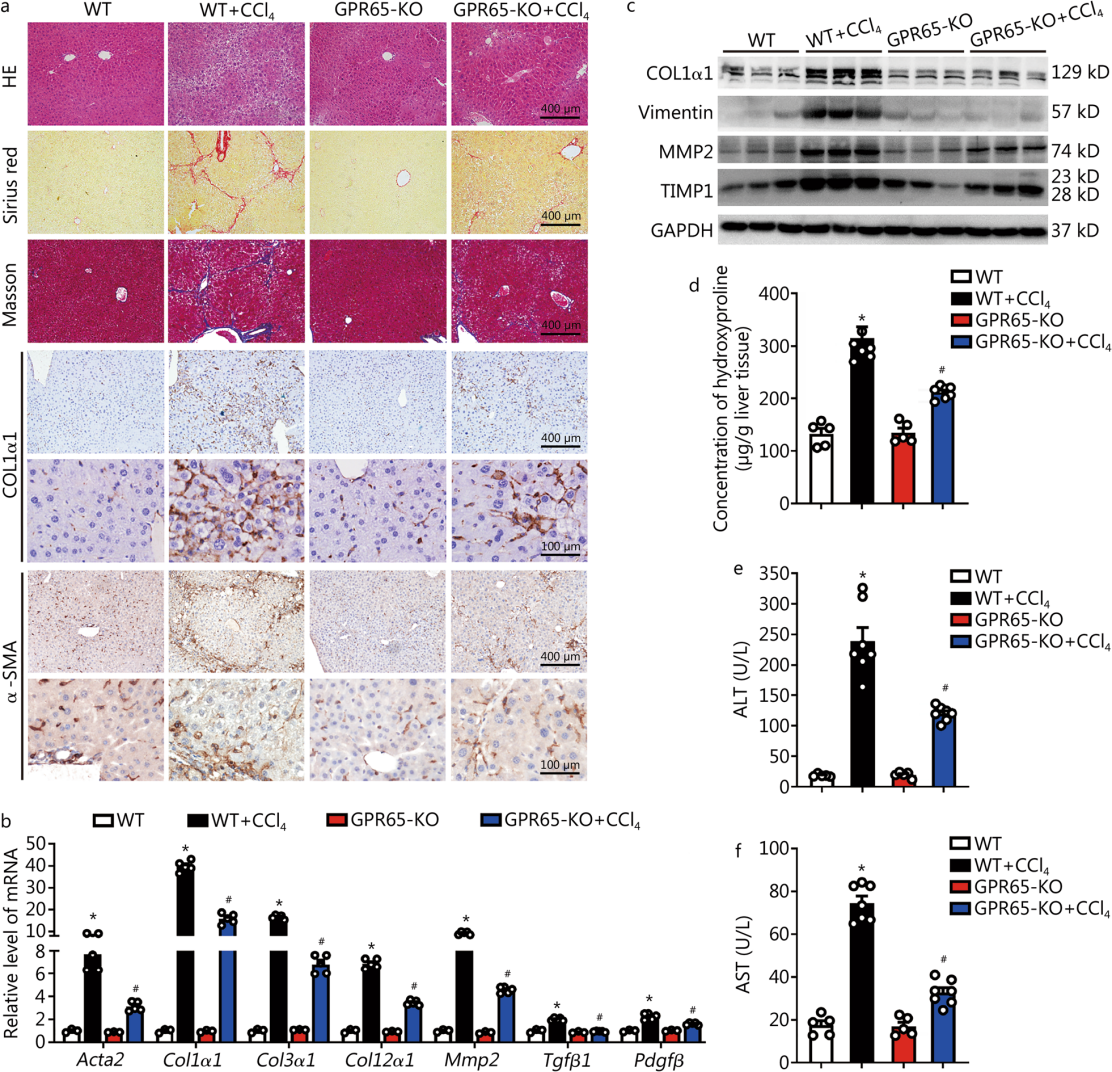
*Gpr65* deficiency alleviates hepatic fibrosis induced by CCl_4_. **a** WT and GPR65-KO mice were divided into 4 groups: WT, WT + CCl_4_, GPR65-KO and GPR65-KO + CCl_4_. Hepatic fibrosis was evaluated by HE staining, Sirius red staining, Masson’s trichrome staining and IHC for COL1α1 and α-SMA. Scale bar = 100 μm for 40 × and 400 μm for 10 × . **b** qRT-PCR was used to assess the mRNA level of *Acta2*, *Col1α1*, *Col3α1*, *Col12α1*, *Mmp2*, *Tgfβ1* and *Pdgfβ* (*n* = 3, 5, 3, 5). **c** Western blotting was used to determine the protein level of COL1α1, vimentin, MMP2 and TIMP1. **d** The content of hepatic hydroxyproline was quantified in livers of each group (*n* = 5, 7, 5, 7). Serum ALT (**e**) and AST (**f**) level were examined (*n* = 5, 7, 5, 7). ^*^P < 0.05 vs. WT; ^#^*P* < 0.05 vs. WT + CCl_4_. CCl_4_ carbon tetrachloride, IHC immunohistochemistry, KO knockout, qRT-PCR quantitative real-time reverse transcription-polymerase chain reaction, COL1α1 collagen type I alpha 1, α-SMA α-smooth muscle actin, MMP2 matrix metalloproteinase 2, TIMP1 tissue inhibitor of metalloproteinases 1, ALT alanine aminotransferase, AST aspartate aminotransferase

Considering the heterogeneity of hepatic fibrosis, we further examined GPR65 function with a CCl_4_-induced hepatic fibrosis model. GPR65-KO mice or WT mice were treated with or without CCl_4_ for 8 weeks, and CCl_4_ did not change the level of *Gpr65* in the livers of GPR65-KO mice (Additional file 1: Fig. S5a, b). Similar to the results of the BDL model, compared to the WT mice, the GPR65-KO mice exhibited markedly decreased hepatic fibrosis following CCl_4_ treatment, as measured by HE staining, Sirius red staining, Masson’s trichrome staining, IHC, Western blotting and qRT-PCR for pro-fibrotic genes, as well as liver hydroxyproline content (Fig. 3a–d). Furthermore, the degree of CCl_4_-induced hepatic injury demonstrated by HE staining, serum ALT and AST level, and the level of genes related with apoptosis and metabolism in the livers were alleviated in GPR65-KO mice compared to WT controls (Fig. 3a, e, f; Additional file 1: Fig. S5c–f). Additionally, IHC, qRT-PCR and Western blotting results revealed that the expression of pro-inflammatory genes was also markedly decreased in CCl_4_-treated GPR65-KO mice compared to CCl_4_-treated WT controls (Additional file 1: Fig. S5c, f, g).

As HMs are heterogeneous cell populations, consisting of liver-resident macrophage KCs and BMMs that are recruited from the circulation [16, 17], to further distinguish the roles of BMMs or KCs GPR65 in fibrosis development, we used irradiation and BMT to create chimeric mice with GPR65 inactivation only in bone marrow cells. Following treatment with CCl_4_ thrice a week for 6 weeks, mice containing BMM *Gpr65* knockout exhibited attenuated hepatic fibrosis, inflammation and injury than WT/BMT-WT controls, as demonstrated by HE staining, Sirius red staining, Masson’s trichrome staining, qRT-PCR, Western blotting, liver hydroxyproline content as well as serum ALT and AST level (Additional file 1: Fig. S6a-f). All these results demonstrate that *Gpr65* depletion alleviates CCl_4_-induced hepatic inflammation, injury and fibrosis in vivo*,* and the protective effect of *Gpr65* knockout is primarily mediated by BMMs.

### GPR65 promotes macrophage M1 polarization

**Fig. 4:**
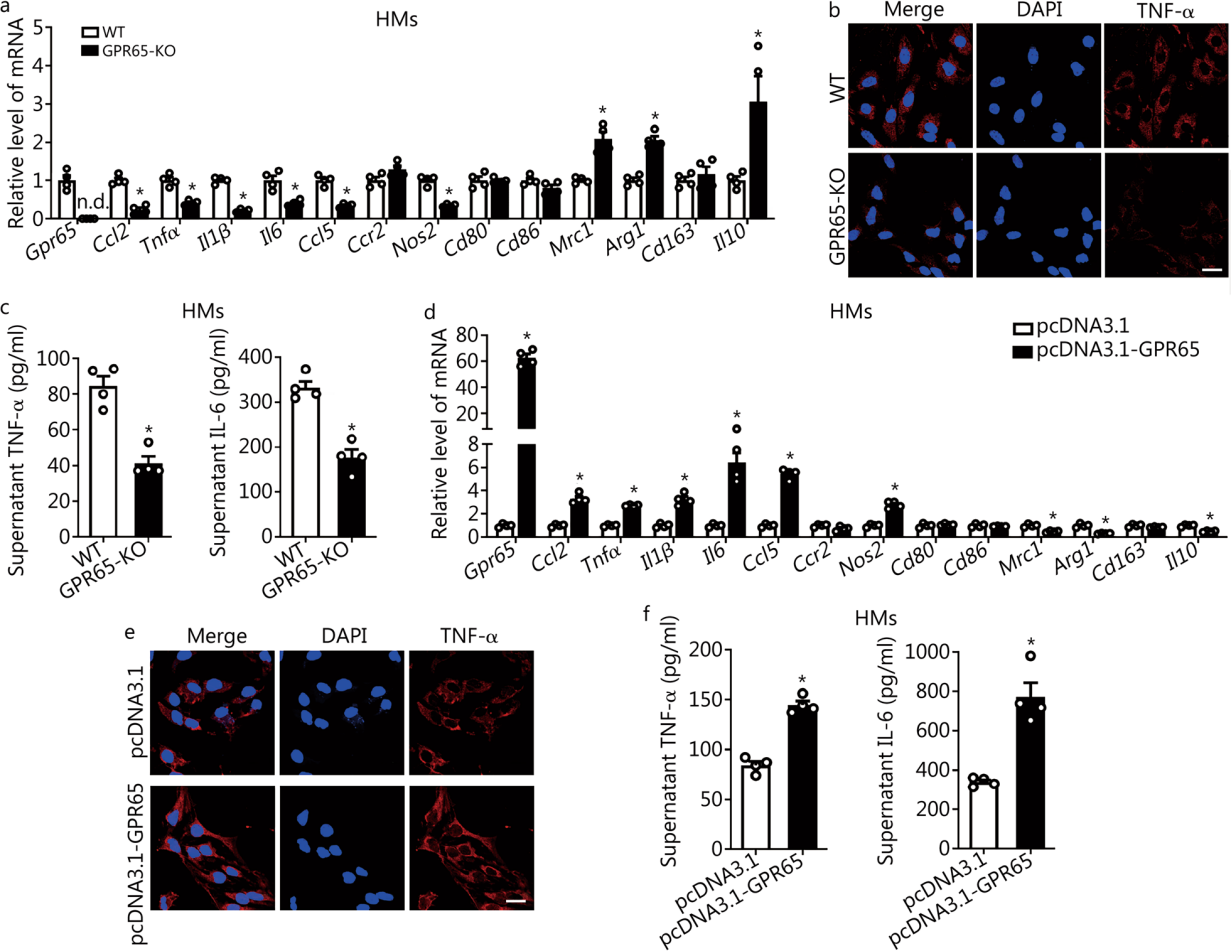
GPR65 promotes M1 macrophage polarization in vitro*.* **a** qRT-PCR was used to assess the mRNA level of *Gpr65*, *Ccl2*, *Tnfα*, *Il1β*, *Il6*, *Ccl5*, *Ccr2*, *Nos2*, *Cd80*, *Cd86*, *Mrc1*, *Arg1*, *Cd163* and *Il10* in HMs isolated from WT and GPR65-KO mice (*n* = 4). **b** The expression and location of TNF-α in HMs isolated from WT and GPR65-KO mice were assessed by confocal microscopy. Scale bar = 20 μm. **c** TNF-α and IL-6 level in the supernatant of HMs isolated from WT and GPR65-KO mice were detected by ELISA (*n* = 4). HMs were transfected with pcDNA3.1 or pcDNA3.1-GPR65 for 48 h, qRT-PCR was used to assess the mRNA level of *Gpr65*, *Ccl2*, *Tnfα*, *Il1β*, *Il6*, *Ccl5*, *Ccr2*, *Nos2*, *Cd80*, *Cd86*, *Mrc1*, *Arg1*, *Cd163* and *Il10* (*n* = 4) (**d**); the expression and location of TNF-α was assessed by confocal microscopy (**e**). Scale bar = 20 μm. TNF-α and IL-6 level in the supernatant were detected by ELISA (*n* = 4) (**f**). ^*^P < 0.05 vs. WT or pcDNA3.1. ELISA enzyme-linked immunosorbent assay, HM hepatic macrophage, KO knockout, qRT-PCR quantitative real-time reverse transcription-polymerase chain reaction, TNF-α tumor necrosis factor-α, IL-6 interleukin-6, n.d. not detected

Given the evidences that macrophage-enriched GPR65 was increased in HMs from the fibrotic livers and *Gpr65* depletion ameliorated CCl_4_- and BDL-induced hepatic inflammation in vivo, we investigated the role of GPR65 on the polarization of macrophage in vitro. Thus, the expression of inflammation-related genes *Ccl2*, *Tnfα*, *Il1β*, *Il6*, *Ccl5*, *Ccr2*, *Nos2*, *Cd80*, *Cd86*, *Mrc1*, *Arg1*, *Cd163* and *Il10* was detected in HMs, which were isolated from GPR65-KO mice or WT mice. The results revealed that the level of pro-inflammatory genes including *Ccl2*, *Tnfα*, *Il1β*, *Il6*, *Ccl5* and *Nos2* was significantly decreased, whereas the level of anti-inflammatory gene *Mrc1*, *Arg1* and *Il10* was markedly increased in GPR65-KO HMs compared to WT controls (Fig. 4a). Similarly, confocal microscopy revealed that the level of TNF-α was decreased in GPR65-KO HMs (Fig. 4b). Moreover, mature TNF-α and IL-6 levels in the supernatant were assessed by enzyme-linked immunosorbent assay (ELISA), and the data indicated that the levels of TNF-α and IL-6 were downregulated in GPR65-KO HMs compared to WT controls (Fig. 4c). On the other hand, forced expression of GPR65 remarkably promoted the expression of the pro-inflammatory genes and the release of TNF-α and IL-6 in HMs as assessed by qRT-PCR, confocal microscopy and ELISA (Fig. 4d–f). Consequently, similar results were also obtained in BMMs and RAW264.7 cells (Additional file 1: Fig. S7, S8a–f). In addition, the expression of proliferation-related genes, *Pcna*, *Ki67*, *Ccnd1* and *Ccne1*, in GPR65-KO and GPR65-overexpressed HMs was also detected, and the data revealed that either knockdown or overexpression of *Gpr65* did not regulate the level of proliferation-related genes (Additional file 1: Fig. S8g, h). CCK-8 assay further confirmed that GPR65 did not affect macrophage proliferation (Additional file 1: Fig. S8i). All these results indicate that GPR65 promotes macrophage M1 polarization in vitro*.*

### Extracellular acidification differentially regulates macrophage polarization partly via GPR65

As GPR65 has been accepted as a proton-sensing GPCR and both anti-inflammatory and pro-inflammatory role of extracellular acidosis have been reported [11, 12], we explored the effect of extracellular acidosis on macrophage and, whether or not, it was dependent on GPR65. The expression of inflammation-related genes was examined in macrophage treated with various pH, including the physiological pH 7.4, the acidic pH 6.6 and the alkalic pH 8.2. Extracellular acidification promoted the level of the pro-inflammatory genes and the release of TNF-α and IL-6, while extracellular alkalization inhibited the level of these inflammation-related genes in HMs as assessed by qRT-PCR, confocal microscopy and ELISA (Additional file 1: Fig. S9a–c). Moreover, the upregulated levels of TNF-α, IL-6 and IL-1β in response to extracellular acidification were ablated in GPR65-KO HMs, suggesting that acidosis promoted M1 macrophage polarization of HMs partly via GPR65 (Additional file 1: Fig. S9d–f). However, both extracellular acidification and alkalization inhibited the level of the pro-inflammatory factors in BMMs, and the down-regulated expression of *Ccl2*, *Tnfα* and *Il6* in response to extracellular alkalization was not further decreased in GPR65-KO BMMs (Additional file 1: Fig. S10a–d), suggesting that acidosis had distinct effects on macrophage by various mechanisms and alkalosis inhibited M1 macrophage polarization of BMMs partly via GPR65. Subsequently, we also examined the role of extracellular acidosis on RAW264.7 cells, and the results revealed that extracellular acidification promoted the expression and release of the pro-inflammatory genes *Tnfα*, *Nos2*, *Ccl5*, *Cxcl10* rather than *Ccl2*, *Il1β* and *Il6*, while extracellular alkalization inhibited the expression and release of the pro-inflammatory factors as assessed by qRT-PCR, confocal microscopy and ELISA (Additional file 1: Fig. S11a–c). In addition, knockdown of *Gpr65* by two different specific siRNAs significantly abrogated overexpression of *Tnfα* and *Ccl5* in response to extracellular acidification (Additional file 1: Fig. S11c–e). All these results reveal that acidosis differentially regulates macrophage polarization and alkalosis inhibits M1 macrophage polarization partly via GPR65.

### Effect of GPR65 exogenous agonist and inhibitor on macrophage polarization

It has been reported that BTB09089 is an allosteric agonist of GPR65 and ZINC62678696 functions as an allosteric inhibitor of GPR65 [23]. To further study the function of GPR65 on macrophage polarization, the GPR65 exogenous agonist and inhibitor were used to treat HMs from GPR65-KO or WT mice. GPR65 inhibitor ZINC62678696 remarkably reduced the expression and release of the pro-inflammatory genes in HMs from WT mice and GPR65-overexpressed HMs, rather than HMs from GPR65-KO mice, suggesting that ZINC62678696 inhibited M1 macrophage polarization of HMs via GPR65 (Additional file 1: Fig. S12a–i). Unexpectedly, GPR65 agonist BTB09089 did not affect the expression of most inflammation-related genes at pH 7.4 and pH 6.6 (Additional file 1: Fig. S12a–c, j). Moreover, BTB09089 slightly enhanced the expression of *Il6* in HMs at pH 8.2 (Additional file 1: Fig. S12k). However, BTB09089 obviously promoted the expression and release of the pro-inflammatory factors in BMMs at both physiological, acidic and alkalic pH, and *Gpr65* depletion ablated this overexpression in response to GPR65 agonist (Additional file 1: Fig. S13a–e). On the other hand, ZINC62678696 remarkably reduced the expression and release of the pro-inflammatory factors in BMMs (Additional file 1: Fig. S13a–c). The data were further confirmed in RAW264.7 cells shown in Additional file 1: Fig. S14a–e. Additionally, BTB09089 remarkably enhanced the level and release of the pro-inflammatory factors except *Il1β* and *Ccl5* in RAW264.7 cells at both physiological and alkalic pH, and knockdown of *Gpr65* significantly abrogated overexpression of TNF-α and IL-6 in response to BTB09089 (Additional file 1: Fig. S14a, c, f, g). All these data demonstrate that ZINC62678696 delays, while BTB09089 promotes macrophage M1 polarization via GPR65.

### GPR65 promotes HSCs activation indirectly by paracrine secretion of TGF-β from macrophage

**Fig. 5:**
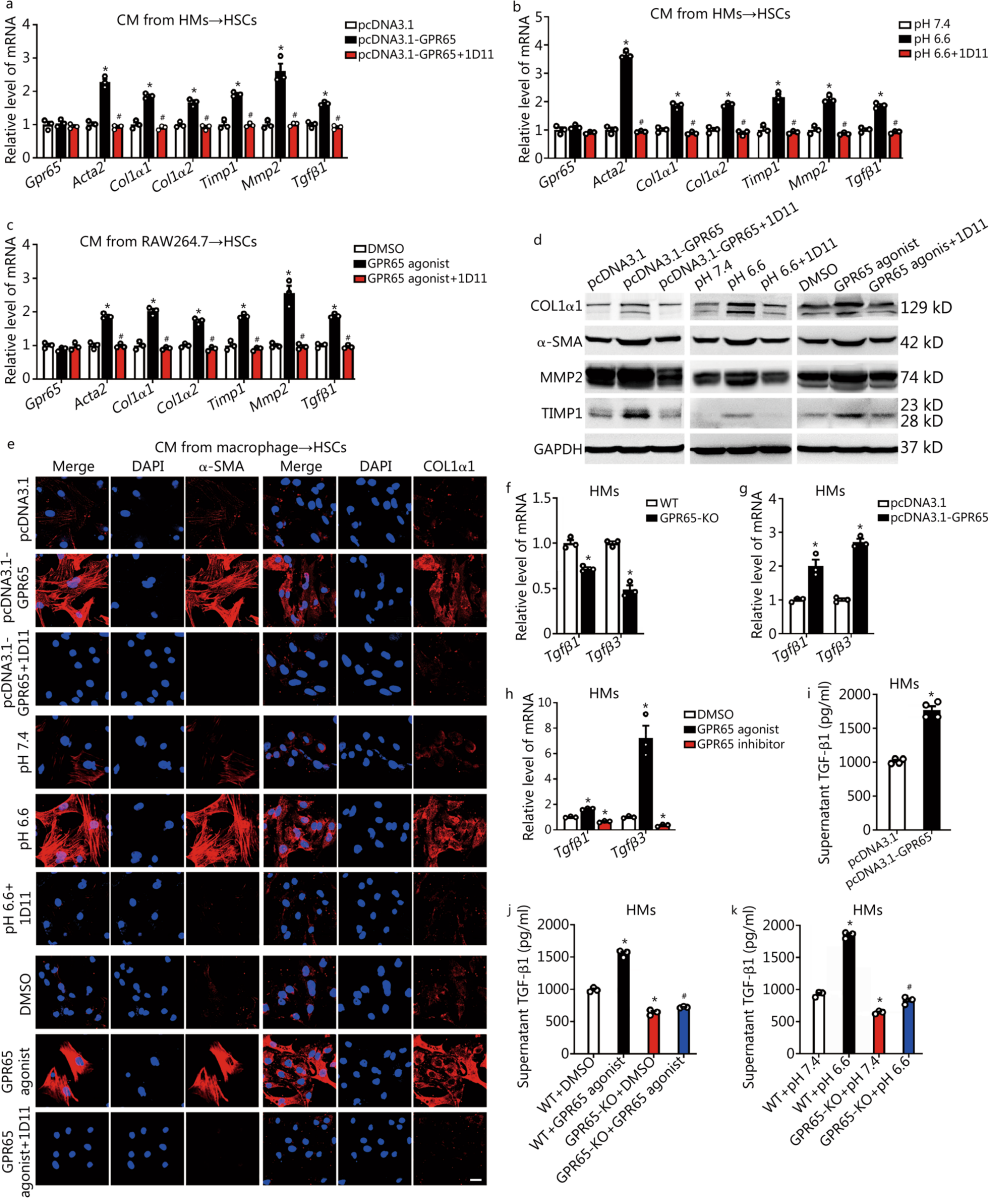
GPR65 promotes HSCs activation indirectly by paracrine secretion of TGF-β from macrophage. The CM from control, GPR65-overexpressed, pH 6.6-treated or GPR65 agonist-treated macrophage cells, with 20 μg/ml TGF-β neutralization antibody 1D11 or mouse IgG, were used to treat primary HSCs for 24 h. qRT-PCR was used to assess the mRNA level of *Gpr65*, *Acta2*, *Col1α1*, *Col1α2*, *Timp1*, *Mmp2* and *Tgfβ1* (*n* = 3) (**a-c**); Western blotting was used to determine the protein level of COL1α1, α-SMA, MMP2 and TIMP1 (**d**); the expression and location of α-SMA and COL1α1 was assessed by confocal microscopy (**e**). Scale bar = 20 μm. qRT-PCR was used to assess the expression of *Tgfβ1* and *Tgfβ3* in GPR65-KO (**f**), *Gpr65*-overexpressed (**g**), or GPR65 agonist/inhibitor (**h**) treated HMs (*n* = 3). **i** TGF-β1 level in the supernatant of HMs transfected with pcDNA3.1 or pcDNA3.1-GPR65 was detected by ELISA (*n* = 4). HMs isolated from WT and GPR65-KO mice were treated with or without GPR65 agonist (**j**) or pH 6.6 (**k**) for 24 h, TGF-β1 level in the supernatant was detected by ELISA (*n* = 3). ^*^P < 0.05 vs. pcDNA3.1, pH 7.4, DMSO, WT, or WT + DMSO/pH 7.4; ^#^*P* < 0.05 vs. pcDNA3.1-GPR65, pH 6.6, GPR65 agonist, or WT + GPR65 agonist/pH 6.6. CM conditioned medium, ELISA enzyme-linked immunosorbent assay, HM hepatic macrophage, HSC hepatic stellate cell, KO knockout, qRT-PCR quantitative real-time reverse transcription-polymerase chain reaction, COL1α1 collagen type I alpha 1, α-SMA α-smooth muscle actin, MMP2 matrix metalloproteinase 2, TIMP1 tissue inhibitor of metalloproteinases 1, TGF-β1 transforming growth factor-β1

Activated HSCs are the central cellular player that promote ECM deposition in response to accumulated inflammatory signals [17, 19]. Thus, we explored the role of GPR65 on HSCs activation. We overexpressed GPR65 in mouse primary HSCs and LX-2 cells as its expression was quite low in these cells. Subsequently, the expression of the pro-fibrotic genes including *ACTA2*, *COL1α1*, *COL1α2*, *TIMP1*, *MMP2* and *TGFβ1* was detected. The results revealed that only *Col1α2* and *Tgfβ1* were upregulated by forced expression of GPR65 in HSCs, but not LX-2 cells (Additional file 1: Fig. S15a, b). Furthermore, both the endogenous and exogenous ligands of GPR65 did not regulate the level of these fibrotic genes in HSCs and LX-2 cells, suggesting that GPR65 was not involved in regulation of HSCs activation directly (Additional file 1: Fig. S15c–f). We, therefore, hypothesized that upregulated GPR65 in macrophage during hepatic fibrogenesis resulted in HMs M1 polarization, which subsequently induced HSCs activation. Hence, CM from control, GPR65-overexpressed, pH 6.6-treated or BTB09089-treated macrophage cells were used to incubate mouse primary HSCs. The results revealed that treatment with the CM from GPR65-overexpressed, pH 6.6- or BTB09089-treated macrophage cells markedly promoted the level of the pro-fibrotic genes, which was abrogated by TGF-β neutralizing antibody 1D11 (Fig. 5a–e). Moreover, forced expression of GPR65, pH 6.6 and BTB09089 promoted, while knockdown of *Gpr65*, pH 8.2 and GPR65 antagonist ZINC62678696 remarkably reduced the expression of *Tgfβ1* and *Tgfβ3* in HMs and RAW264.7 cells (Fig. 5f–h; Additional file 1: Fig. S16a–h). As expected, supernatant TGF-β1 level in GPR65-overexpressed, pH 6.6-treated or BTB09089-treated HMs, BMMs and RAW264.7 cells was obviously increased (Fig. 5i-k; Additional file 1: Fig. S16i–s), suggesting that TGF-β1 is one of the factors derived from macrophage that induces HSCs activation. Altogether, our data demonstrate that GPR65 promotes the activation of HSCs via the signals derived from macrophage.

### GPR65 aggravates HCs damage through the signals derived from macrophage

HCs damage or apoptosis has been widely recognized as a key initiator of fibrosis in ongoing hepatic injury [20, 21]. Given the evidences that *Gpr65* deletion ameliorates CCl_4_- and BDL-induced hepatic damage in vivo, we explored the effect of GPR65 on HCs. We overexpressed GPR65 in HCs and AML12 cells as GPR65 was low expressed in HCs and minimally expressed in AML12 cells. Subsequently, the expression of apoptosis-related genes (*Bax*, *Bad*), inflammation-related genes (*Il1β*, *Ccl2*) and metabolism-related genes (*Sult1β1*, *Ugt2β1*, *Cyp2α5*, *Cyp4α12*, *Gsta2*, *Nox2*, *Slc25α1*) was detected. The data demonstrated that forced expression of GPR65, the endogenous and exogenous ligands only slightly regulated the level of *Il1β* rather than apoptosis-, inflammation- and metabolism-related genes (Additional file 1: Fig. S17a–f), suggesting that GPR65 regulated HCs damage and metabolism mainly in an indirect manner. Considering that GPR65 promotes the level and release of pro-inflammatory genes and TGF-β, which may subsequently act on HCs to induce damage, we also used CM from control, GPR65-overexpressed, pH 6.6-treated or BTB09089-treated macrophage cells to incubate mouse primary HCs. The results revealed that treatment with the CM from GPR65-overexpressed, pH 6.6-treated or BTB09089-treated macrophage cells obviously promoted HC damage as characterized by the overexpression of pro-apoptotic genes (*Bad* and *Bax*), the apoptosis-related morphologic changes, the increased level of cleaved caspase-3 and BAX (Additional file 1: Fig. S17g–k). All these data demonstrate that GPR65 aggravates HCs damage and metabolism dysfunction through the signals derived from macrophage.

### Targeting GPR65 alleviates HMs inflammation and fibrosis by suppressing the Gαq-Ca^2+^-JNK/NF-κB pathways

To explore the downstream signaling of GPR65, we first examined the level of various Gα subunits including *Gα11*, *Gαq*, *Gαi1*, *Gαi2*, *Gαi3*, *Gαs* and *Gα13* in HMs, RAW264.7 cells and BMMs. The data revealed that all types of macrophages expressed *Gα11*, *Gαq*, *Gαi2*, *Gαi3* and *Gα13*, but not Gαs. Moreover, *Gαi1* was expressed in HMs rather than RAW264.7 cells and BMMs (Additional file 1: Fig. S18a), reflecting the distinct responses to GPR65 of these cells. Next, specific siRNAs targeting *Gαi2*, *Gαi3*, *Gα11*, *Gα13* and *Gαq* were used to knockdown these Gα subunits in RAW264.7 cells treated with BTB09089, and subsequently the level of *Tnfα*, *Il6* and *Tgfβ3* was detected. The data revealed that knockdown of *Gαq* but not *Gαi2*, *Gαi3*, *Gα11* and *Gα13* attenuated BTB09089-induced up-regulation of *Tnfα*, *Il6* and *Tgfβ3* (Additional file 1: Fig. S18b–f). Moreover, Gαq silencing or Gαq inhibitor YM254890 blocked GPR65 overexpression-, GPR65 agonist-, or pH6.6-induced up-regulation of IL-6, TNF-α and TGF-β in RAW264.7 cells and HMs (Additional file 1: Figs. S18g–k, S19). As the Ca^2+^-AMPK signal is an important component of Gαq signaling [24], BAPTA (Ca^2+^ chelator, 10 μmol/L) and Compound C (AMPK inhibitor, 10 μmol/L) were also applied in GPR65-overexpressed HMs, and the data revealed that BAPTA rather than Compound C rescinded GPR65 overexpression induced up-regulation of TGF-β, TNF-α and IL-6 (Additional file 1: Fig. S20), suggesting that Gαq-Ca^2+^ mediates GPR65-induced up-regulation of TNF-α, IL-6 and TGF-β.

**Fig. 6:**
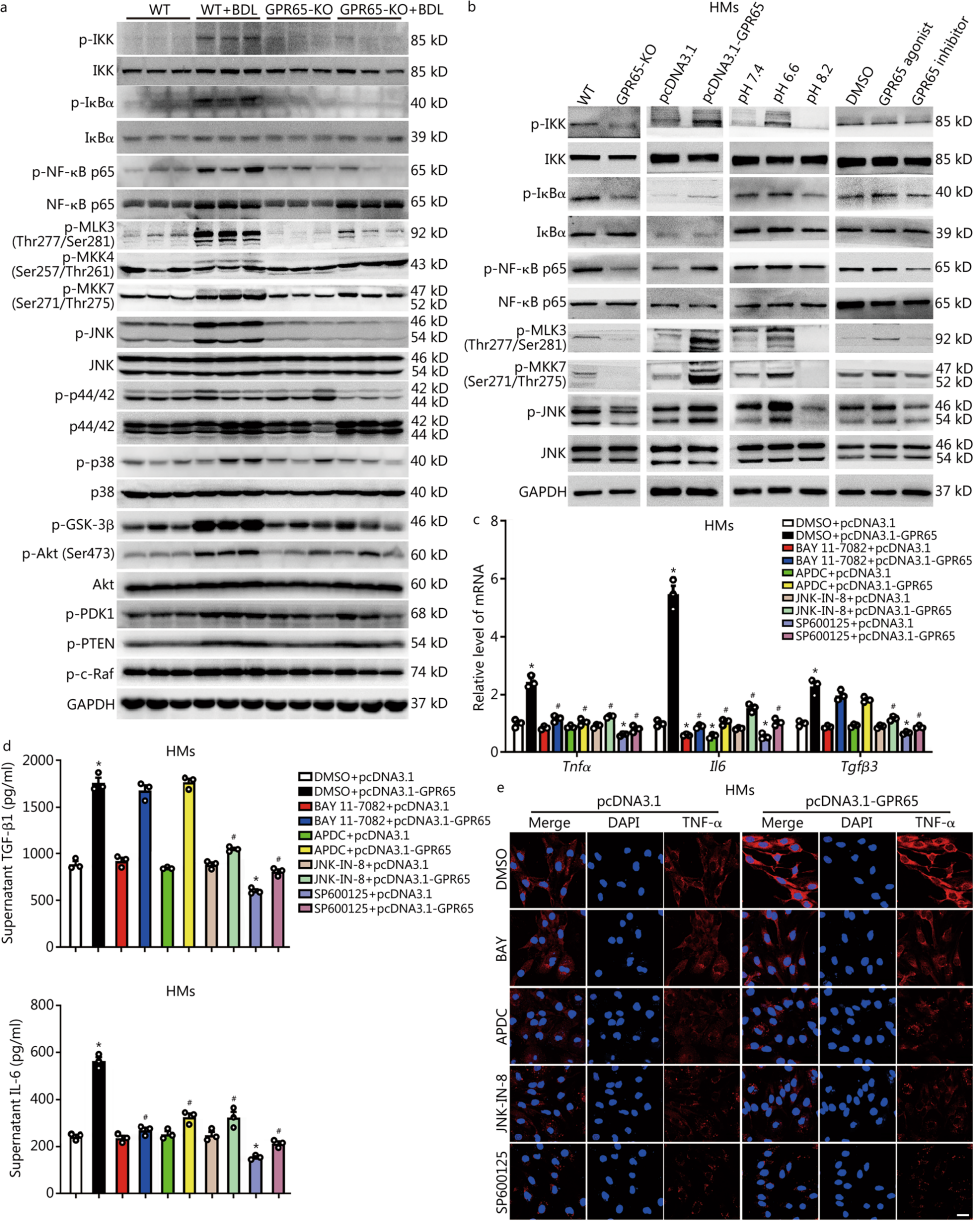
Targeting GPR65 alleviates hepatic macrophage inflammation and fibrosis by suppressing the JNK/NF-κB pathways. **a** Western blotting was used to determine the level of p-IKK, p-IĸBα, p-NF-κB p65, IKK, IĸBα, NF-κB p65, p-MLK3, p-MKK4, p-MKK7, p-JNK, JNK, p-p44/42, p44/42, p-p38, p38, p-GSK-3β, p-Akt, Akt, p-PDK1, p-PTEN and p–c-Raf in liver tissues from WT, WT + BDL, GPR65-KO and GPR65-KO + BDL mice. **b** Western blotting was used to determine the level of p-IKK, IKK, p-IĸBα, IĸBα, p-p65, p65, p-MLK3, p-MKK7, p-JNK and JNK in GPR65-KO, GPR65-overexpressed, various pH-treated and GPR65 agonist/inhibitor-treated HMs. The specific inhibitors of JNK, SP600125 (10 μmol/L) and JNK-IN-8 (5 μmol/L), as well as the specific inhibitors of NF-κB, BAY 11–7082 (5 μmol/L) and APDC (20 μmol/L), were used to treat GPR65-overexpressed HMs for 24 h, and qRT-PCR was used to assess the expression of *Tnfα*, *Il6* and *Tgfβ3* (*n* = 3) (**c**); TGF-β1 and IL-6 level in the supernatant were detected by ELISA (*n* = 3) (**d**); the expression and location of TNF-α was assessed by confocal microscopy (**e**). Scale bar = 20 μm. ^*^P < 0.05 vs. DMSO + pcDNA3.1; ^#^*P* < 0.05 vs. DMSO + pcDNA3.1-GPR65. CCl_4_ carbon tetrachloride, BDL bile duct ligation, ELISA enzyme-linked immunosorbent assay, HM hepatic macrophage, KO knockout, qRT-PCR quantitative real-time reverse transcription-polymerase chain reaction, IKK inhibitor of ĸB kinase, IĸBα inhibitor of ĸB α, NF-κB p65 nuclear factor κB p65 subunit, MLK3 mixed lineage kinase 3, MKK4 mitogen-activated protein kinase kinase 4, MKK7 mitogen-activated protein kinase kinase 7, JNK c-Jun N-terminal kinase, GSK-3β glycogen synthase kinase 3β, Akt protein kinase B, PDK1 pyruvate dehydrogenase kinase 1, PTEN phosphatase and tensin homolog, c-Raf c-rapidly accelerated fibrosarcoma, TGF-β1 transforming growth factor-β1, IL-6 interleukin-6, TNF-α tumor necrosis factor-α

Since the KEGG pathway and GO analysis indicated that *Gpr65* depletion affected a serial of genes related with PI3K-Akt, MAPK and NF-ĸB pathways (Fig. 2c–e), we firstly measured the level of p-IKK, p-IĸBα, p-NF-ĸB p65, IKK, IĸBα, NF-ĸB p65, p-MLK3 (Thr277/Ser281), p-MKK4 (Ser257/Thr261), p-MKK7 (Ser271/Thr275), p-JNK, p-p44/42, p-p38, JNK, p44/42, p38, p-GSK-3β, p-Akt (Ser473), p-PDK1, p-PTEN, p-c-Raf and Akt in liver tissues from BDL-/CCl_4_-induced WT and GPR65-KO mice. Western blotting analysis revealed that *Gpr65* depletion obviously ameliorated BDL- and CCl_4_-induced phosphorylation of IKK, IĸBα, NF-ĸB p65, MLK3, MKK7 and JNK, while slightly inhibited phosphorylation of p38, Akt (Ser473) and c-Raf (Fig. 6a; Additional file 1: Fig. S21a). Unexpectedly, *Gpr65* depletion did not regulate BDL- and CCl_4_-induced phosphorylation of MKK4, p44/42, PDK1 and PTEN (Fig. 6a; Additional file 1: Fig. S21a). Moreover, forced expression of GPR65, pH 6.6 and BTB09089 significantly promoted, while knockout of *Gpr65*, pH 8.2 and ZINC62678696 reduced the phosphorylation of IKK, IĸBα, NF-ĸB p65, MLK3, MKK7 and JNK in HMs and RAW264.7 cells (Fig. 6b; Additional file 1: Fig. S21b), suggesting that silencing GPR65 alleviated hepatic fibrosis and M1 macrophage polarization mainly through the JNK and NF-κB pathways. Subsequently, SP600125 and JNK-IN-8, the specific inhibitors of JNK, as well as BAY 11-7082 and APDC, the specific inhibitors of NF-κB, were applied in GPR65-overexpressed, pH 6.6-treated or BTB09089-treated HMs and RAW264.7 cells, and the data revealed that SP600125 and JNK-IN-8 rather than BAY 11-7082 and APDC rescinded GPR65 overexpression, pH 6.6 and BTB09089-induced up-regulation of TGF-β, while both JNK inhibitors and NF-κB inhibitors abrogated GPR65 overexpression, pH 6.6 and BTB09089-induced up-regulation of TNF-α and IL-6 (Fig. 6c–e; Additional file 1: Figs. S21c–e, S22). These data demonstrate that GPR65 overexpression, pH 6.6 and BTB09089 induce up-regulation of TNF-α and IL-6 via the Gαq-Ca^2+^-JNK/NF-κB pathways, while promoting the expression of TGF-β through the Gαq-Ca^2+^-MLK3-MKK7-JNK pathway.

### Pharmacological GPR65 inhibition alleviates CCl_4_-induced hepatic fibrosis

**Fig. 7:**
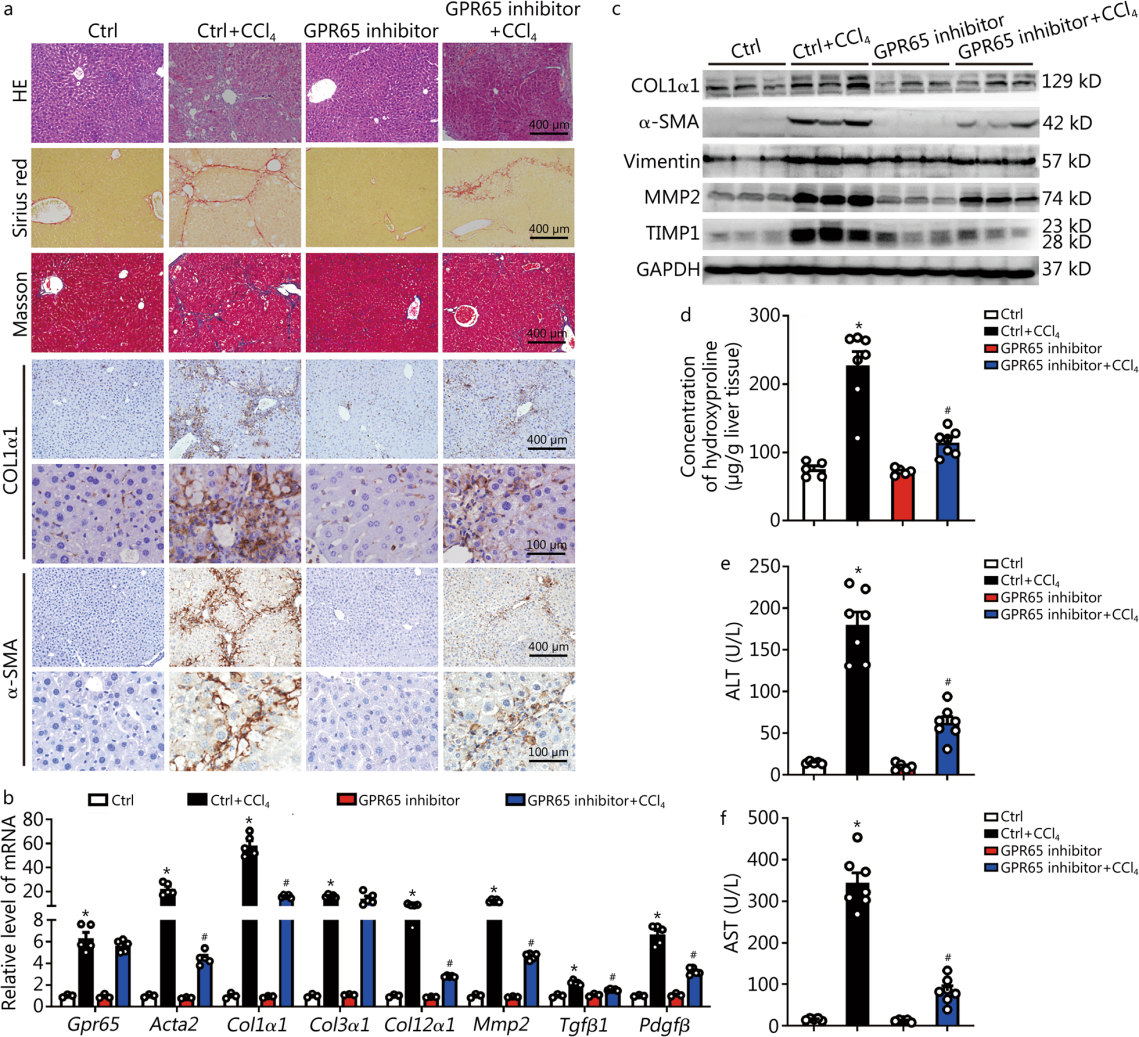
Pharmacological GPR65 inhibition alleviates hepatic fibrosis induced by CCl_4_. **a** BALB/c mice were divided into 4 groups randomly: Ctrl, Ctrl + CCl_4_, GPR65 inhibitor and GPR65 inhibitor + CCl_4_. The GPR65 inhibitor (10 mg/kg) was administered 4 weeks after the first CCl_4_ injection. Mice were administered CCl_4_ for 8 weeks. Hepatic fibrosis was evaluated by HE staining, Sirius red staining, Masson’s trichrome staining and IHC for COL1α1 and α-SMA. Scale bar = 100 μm for 40 × and 400 μm for 10 × . **b** qRT-PCR was used to assess the mRNA level of *Gpr65*, *Acta2*, *Col1α1*, *Col3α1*, *Col12α1*, *Mmp2*, *Tgfβ1* and *Pdgfβ* (*n* = 3, 5, 3, 5). **c** Western blotting was used to determine the protein level of COL1α1, α-SMA, vimentin, MMP2 and TIMP1. **d** The content of hepatic hydroxyproline was quantified in livers of each group (*n* = 5, 7, 5, 7). Serum ALT (**e**) and AST (**f**) were examined (*n* = 5, 7, 5, 7). ^*^P < 0.05 vs. Ctrl; ^#^*P* < 0.05 for vs. Ctrl + CCl_4_. CCl_4_ carbon tetrachloride, Ctrl control, IHC immunohistochemistry, qRT-PCR quantitative real-time reverse transcription-polymerase chain reaction, COL1α1 collagen type I alpha 1, α-SMA α-smooth muscle actin, MMP2 matrix metalloproteinase 2, TIMP1 tissue inhibitor of metalloproteinases 1, ALT alanine aminotransferase, AST aspartate aminotransferase

We next explored the therapeutic potential of targeting GPR65 in the development of hepatic fibrosis. ZINC62678696 (10 μg/g) was administered intraperitoneally every two days in the CCl_4_-treated or oil-treated mice 4 weeks after the first injection of CCl_4_. After 8 weeks of CCl_4_ treatment, we tested whether the inhibition of GPR65 alleviated CCl_4_-induced hepatic fibrosis in vivo. As shown in Fig. 7a–d, GPR65 inhibition significantly attenuated CCl_4_-induced hepatic fibrosis, as evidenced by HE staining, Sirius red staining, Masson’s trichrome staining, IHC, Western blotting and qRT-PCR for pro-fibrogenic genes, as well as liver hydroxyproline content. Moreover, the degree of CCl_4_-induced hepatic injury, demonstrated by serum ALT and AST level, HE staining, IHC, and the level of genes related with apoptosis and metabolism in the livers, was alleviated by GPR65 inhibitor in the CCl_4_-treated mice (Fig. 7a, e, f; Additional file 1: Fig. S23a-d). Additionally, the CCl_4_-induced hepatic expression of pro-inflammatory genes, as demonstrated by IHC, Western blotting and qRT-PCR, was also significantly reduced by GPR65 inhibitor in vivo (Additional file 1: Fig. S23b, d, e), indicating that GPR65 inhibitor has the potential for clinical transformation in the treatment of hepatic fibrosis. Finally, Western blotting results showed that GPR65 inhibitor significantly blocked CCl_4_-induced phosphorylation of IKK, IκBα, NF-κB p65, MLK3, MKK7 and JNK rather than MKK4, p44/42, p38, GSK-3β and PDK1, suggesting that GPR65 inhibition attenuates the CCl_4_-induced activation of JNK/NF-κB signaling (Additional file 1: Fig. S24a). Taken together, the results reveal that pharmacological GPR65 inhibition alleviates CCl_4_-induced hepatic inflammation, injury and fibrosis in vivo.

## Discussion

**Fig. 8:**
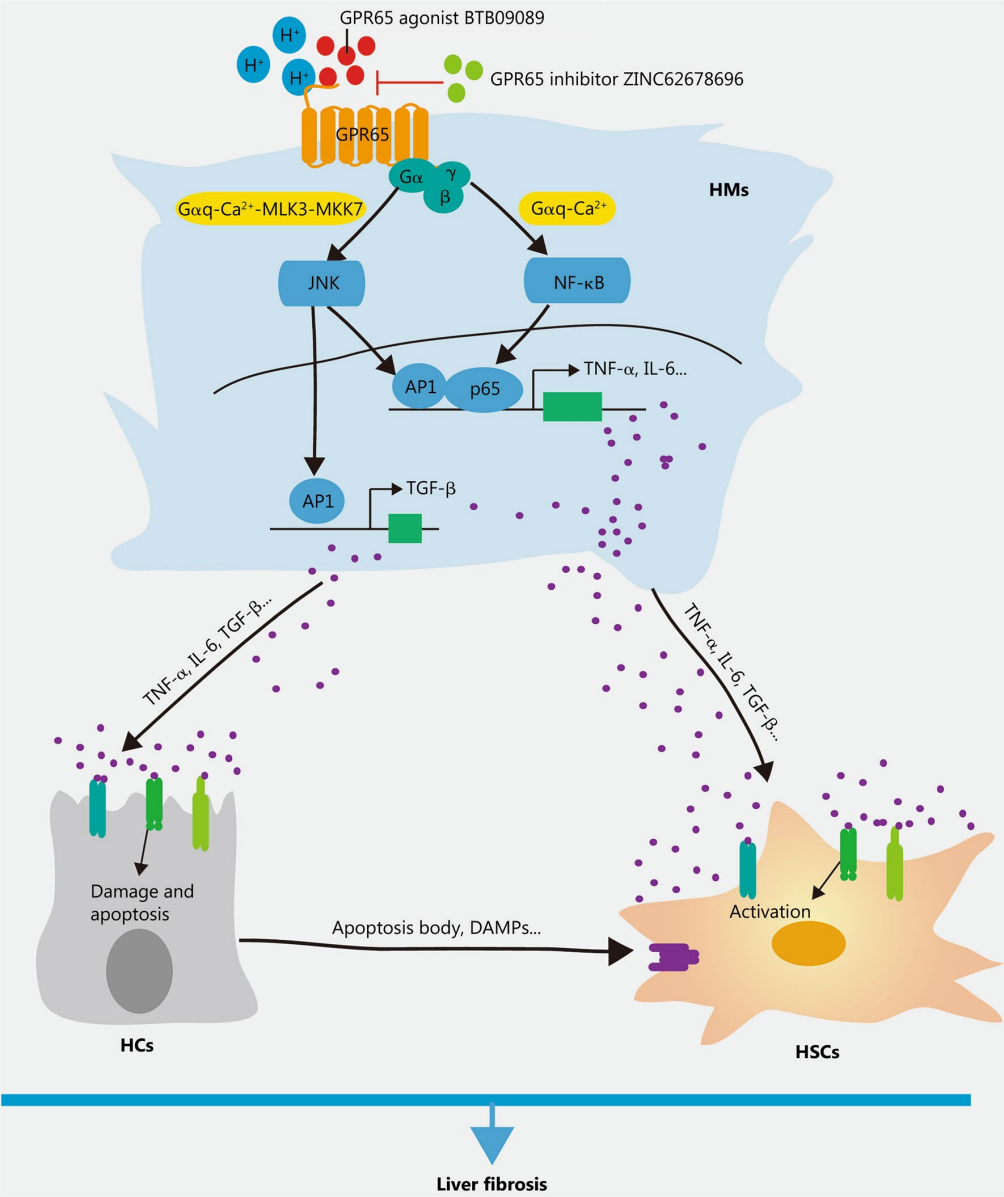
Schematic diagram illustrating the function and mechanism of GPR65 in the progression of liver inflammation and fibrosis. Upon liver injury, increased or activated HMs-enriched GPR65 induces up-regulation of TNF-α and IL-6 via the Gαq-Ca^2+^-JNK/NF-κB pathways, while promoting the expression of TGF-β through the Gαq-Ca^2+^-MLK3-MKK7-JNK pathway in HMs, thus resulting in the damage of HCs and the activation of HSCs, and subsequently aggravate BDL- and CCl_4_-induced liver inflammation, injury and fibrosis. In addition, pharmacological inhibition of GPR65 can block this process. BDL bile duct ligation, CCl_4_ carbon tetrachloride, HC hepatocyte, HM hepatic macrophage, HSC hepatic stellate cell, MLK3 mixed lineage kinase 3, MKK7 mitogen-activated protein kinase kinase 7, NF-κB nuclear factor κB, JNK c-Jun N-terminal kinase, AP1 activator protein 1, TNF-α tumor necrosis factor-α, IL-6 interleukin-6, TGF-β transforming growth factor-β, DAMPs damage-associated molecular patterns

Cirrhosis is the end stage of progressive hepatic fibrosis resulted from a sustained injury repair in response to chronic hepatic injury [1]. Despite continuous progress that has been made during the last thirty years in exploring the cellular and molecular mechanisms of hepatic fibrosis and cirrhosis. However, the prevalence of hepatic cirrhosis is still surging and no specific drugs have been approved. Therefore, the need to develop promising drug targets and corresponding therapeutic molecules remains urgent. GPCRs, the largest family of transmembrane receptors mediating the majority of cellular responses, play a vital role in various pathophysiological processes including cardiovascular diseases, cancer and inflammation-related diseases [6, 25, 26]. Due to their cell membrane localization, diversified expression and clear binding pockets, GPCRs have become attractive drug targets and the sales of GPCR targeted drugs account for more than 27% of the global market share [6]. As GPCRs are widely expressed in various cells, and the functions of certain GPCRs in HCs, HSCs and HMs during hepatic fibrosis remain controversial [7], suggesting that developing therapeutics drugs targeting GPCRs for hepatic fibrosis without compromising other physiological functions is a great challenge. In this study, we have identified that the proton-sensing receptor GPR65, which is a macrophage-enriched GPCR, is upregulated in both human and mouse fibrotic livers as well as primary HMs from fibrotic livers of mice. Both in vivo and in vitro data provided convincing evidences that silencing or the antagonist of GPR65 inhibited, while overexpression or the application of the endogenous and exogenous agonist of this receptor enhanced the expression and release of TNF-α, IL-6 and TGF-β, all of which promoted the activation of HSCs and the damage of HCs, and subsequently aggravate BDL- and CCl_4_-induced hepatic inflammation, injury and fibrosis. Mechanistically, our results demonstrated that GPR65 overexpression, the acidic pH and exogenous GPR65 agonist induced up-regulation of TNF-α and IL-6 via the Gαq-Ca^2+^-JNK/NF-κB pathways, while promoting the expression of TGF-β through the Gαq-Ca^2+^-MLK3-MKK7-JNK pathway. These results suggest that pharmacological inhibition of GPR65 could protect against hepatic fibrosis, thus providing new cues for harnessing hepatic fibrosis by developing innovative therapeutic approaches based on the GPR65-Gαq-Ca^2+^-JNK/NF-κB pathways (Fig. 8).

Although the expression of human GPR65 was not correlated with the degree of fibrosis as demonstrated by the expression of GPR65 was not further increased with the progression of fibrosis and no correlations were found with COL1α1 and ACTA2 (Fig. 1a; Additional file 1: Fig. S2b, c). However, the hepatic level of human GPR65 was correlated with TNF-α, IL-6, both of which reflect the degree of inflammation (Fig. 1b, c). The reason for this is, on one hand, that HMs-enriched GPR65 was not directly involved in the regulation of the activation of HSCs as well as the injury of HCs, but acted on HSCs and HCs through the signals derived from HMs. On the other hand, human hepatic fibrosis is a complex condition influenced by a multitude of factors, including genetic variations, environmental factors, and co-existing comorbidities. These factors could contribute to the heterogeneity and variability observed in fibrosis progression and GPR65 expression patterns among human patients. Interestingly, based on the fact that GPR65-KO mice were not abnormal in size, appearance and mating at the age of 12 months, together with the finding that both GPR65-KO mice and the application of GPR65 inhibitor in vivo did not exhibit any functional and morphological abnormalities (data not shown), it suggests that GPR65 is a promising and specific drug target for hepatic fibrosis, cirrhosis, or even inflammation-related diseases.

GPCRs are integral membrane proteins composed of seven transmembrane α-helices. Upon binding of activating ligands or agonists, a conformational change occurs in the GPCR, allowing it to interact with a heterotrimeric G protein and promote the exchange of GTP for GDP on the Gα subunit [27, 28]. In a simplified model, agonist binding stabilizes the receptor in an active conformation, enabling its interaction with intracellular partners [5]. In our study, we observed that overexpression of GPR65 alone, in the absence of agonist or acid, leads to the up-regulation of pro-inflammatory cytokines (Fig. 4d-f). The underlying mechanism that overexpression of GPR65 induces cytokine production is that there is a certain level of proton concentration in the normal cell culture medium, which is enough or partially to contribute to the activity of overexpressed GPR65 with the exception of baseline activity of the receptor. These findings are in line with previous reports that many GPCRs exhibit a basal level of GTP exchange activity in the absence of an exogenous ligand and often display a degree of basal or constitutive signaling when overexpressed [5, 29].

Hepatic fibrosis is a reversible pathological process, and its reversibility is determined by the microenvironment created by the interaction among HMs, HCs and HSCs as well as pro-fibrotic signals [1, 30]. Emerging evidences demonstrated that inflammation is an important driver of fibrosis [1, 17, 19, 21, 31]. Interestingly, our data revealed that GPR65 does not affect HM number by the following evidences. Firstly, the results of RNA-seq analysis revealed that loss of *Gpr65* results in changed expression of only four genes in the livers of GPR65-KO mice (Fig. 2a), indicating that GPR65-KO did not alter the number of HMs in the normal liver condition. Secondly, the expression of proliferation-related genes *Pcna* and *Ki67* did not significantly change in GPR65-KO mice compared to WT controls, as well as in CCl_4_- and BDL-treated GPR65-KO mice compared to CCl_4_- and BDL-treated WT controls (Additional file 1: Figs. S4c, d, f and S5c, d, f). Thirdly, gain- and loss-of-function experiments in vitro confirmed that overexpression and knockout of *Gpr65* did not regulate the expression of proliferation-related genes (Additional file 1: Fig. S8g-i). However, we found that F4/80 and LY6C positive cells are decreased in GPR65-KO mice after BDL and CCl_4_ treatment (Additional file 1: Figs. S4c, d, f and S5c, d, f). As the increased number of HMs during liver fibrosis was caused by recruitment from blood and self-proliferation. Therefore, we believe that F4/80 and LY6C positive cells are decreased in GPR65-KO mice after BDL and CCl_4_ treatment may be a result of reduced recruited macrophage or the consequence of decreased liver fibrosis.

It is well-known that local acidosis, which is attributed partly to increased lactate production by the inflammatory cells, HCs and gut cells, is the hallmark of many chronic inflammatory diseases including fibrosis and tumor microenvironment [9, 32, 33]. Cells sensing extracellular acidosis have several mechanisms. Ion channels including transient receptor potential V1 and acid-sensing ion channel, mainly expressed on sensory neurons, represent one sensing mechanism [34]. GPR65 together with GPR4, GPR68, and GPR132, all of which identified as proton-sensing GPCRs, represents another type of proton sensing mechanisms [9]. In the present study, we detected these proton-sensing GPCRs in normal and fibrotic liver tissues, and the results revealed that *Gpr4* and *Gpr65* were expressed in livers and only *Gpr65* was overexpressed in fibrotic livers, indicating a crucial role of this receptor in fibrogenesis (Additional file 1: Fig. S24b). To date, both anti-inflammatory and pro-inflammatory activities of GPR65 in response to acidosis have been reported, for instance, loss of *Gpr65* promotes the expression of pro-inflammatory factors, as well as colonic macrophage and neutrophil infiltration in the acute and chronic inflammatory bowel disease [11, 14, 35]. It has been also reported that GPR65 partly mediates the extracellular acidification-induced up-regulation of pro-inflammatory cytokine production in mouse macrophages. Furthermore, GPR65 can also stimulate the inflammatory response and is a member of the module that co-varies with pro-inflammatory genes [36]. Several studies revealed that blocking GPR65 expression and function was related with decreased level of pro-inflammatory cytokines including TNF-α, IL-17 and IFN-γ, reduced synovial inflammation, eosinophil viability and microglial activation [12, 14, 37]. However, the precise impact of GPR65 in response to local acidosis on HMs inflammation, HCs damage, HSCs activation, and hepatic fibrogenesis remains unclear. Our study revealed that extracellular alkalization inhibited the level of the pro-inflammatory genes in HMs, BMMs and RAW264.7 cells, while extracellular acidification promoted the expression and release of the pro-inflammatory genes via GPR65 in HMs and RAW264.7 cells rather than BMMs. Moreover, the pro-inflammatory genes upregulated in HMs and RAW264.7 cells are not absolutely identical. To explain the discrepancy, we detected the level of proton-sensing GPCRs and the downstream various Gα subunits in macrophages, and the results revealed that *Gpr4* and *Gpr65* were highly expressed in HMs, while both BMMs and RAW264.7 cells mainly expressed *Gpr65* and *Gpr132*, followed by moderate expression of *Gpr4* in RAW264.7 cells (Additional file 1: Fig. S24c). In addition, our data showed that all types of macrophages expressed *Gαi2, Gαi3, Gαq, Gα11* and *Gα13*, but not *Gαs,* and *Gαi1* was expressed in HMs rather than BMMs and RAW264.7 cells (Additional file 1: Fig. S18a). The different expression patterns of GPCRs and Gα subunits may partially reflect the distinct responses to acidosis in HMs, BMMs and RAW264.7 cells. Surprisingly, extracellular acidification does not regulate the level of the fibrotic-related genes in HSCs and the expression of apoptosis-, inflammation- and metabolism-related genes, except for *Nox2* and *Slc25α1*, in HCs, suggesting that acidosis does not regulate HSCs activation and HCs damage and metabolism directly. Further investigation on the effect of acid–base microenvironment during liver fibrogenesis is needed.

## Conclusions

In summary, our results provide convincing evidences that the HMs-enriched proton-sensing GPR65 is involved in hepatic fibrosis in both mice and human. Given the role of GPR65 as a physiologic integrator of the microenvironment (especially inflammation condition), these data provide insight into the molecular mechanisms by which the acidosis-activated GPR65 may contribute to the progression of fibrosis. It also brings some understanding of the anti-inflammatory effects of the GPR65 inhibitor, which may open novel avenues of research for drug development in the treatment of hepatic fibrosis and inflammation-related diseases.

### Supplementary Information

**Additional file 1:** Materials and methods. **Table S1** Clinical characteristics of patients. **Table S2** Quantitative real-time reverse transcription-polymerase chain reaction (qRT-PCR) primers for analysis of transcript levels. **Table S3** Cloning primers for GPR65. **Table S4** siRNA sequences. **Fig. S1** Identification of GPR65 during hepatic fibrosis. **Fig. S2** GPR65 is enriched in hepatic macrophage. **Fig. S3** *Gpr65* deficiency alleviates BDL-induced hepatic fibrosis. **Fig. S4** *Gpr65* deficiency alleviates BDL-induced hepatic injury and inflammation. **Fig. S5** *Gpr65* deficiency ameliorates CCl_4_-induced hepatic injury and inflammation. **Fig. S6** *Gpr65* deficiency in BMMs ameliorates CCl_4_-induced hepatic fibrosis, injury and inflammation. **Fig. S7** GPR65 promotes BMMs M1 polarization. **Fig. S8** GPR65 promotes RAW264.7 cell M1 polarization. **Fig. S9** Extracellular pH differentially regulates HMs polarization partly via GPR65. **Fig. S10** Extracellular pH differentially regulates BMMs polarization partly via GPR65. **Fig. S11** Extracellular pH differentially regulates RAW264.7 cells polarization partly via GPR65. **Fig. S12** Effect of GPR65 exogenous agonist and inhibitor on HMs polarization. **Fig. S13** Effect of GPR65 exogenous agonist and inhibitor on BMMs polarization. **Fig. S14** Effect of GPR65 exogenous agonist and inhibitor on RAW264.7 cells polarization. **Fig. S15** GPR65 is not involved in regulation of HSCs activation directly. **Fig. S16** GPR65 promotes the expression and release of TGF-β. **Fig. S17** GPR65 aggravates HCs damage through the signals derived from macrophage. **Fig. S18** Targeting GPR65 alleviates inflammation via Gαq. **Fig. S19** Targeting GPR65 alleviates hepatic macrophage inflammation via Gαq. **Fig. S20** Targeting GPR65 alleviates hepatic macrophage inflammation via Ca^2+^ rather than AMPK. **Fig. S21** Targeting GPR65 alleviates hepatic macrophage inflammation by suppressing the JNK/NF-κB pathways. **Fig. S22** Targeting GPR65 alleviates inflammation by suppressing the JNK/NF-κB pathways. **Fig. S23** Pharmacological GPR65 inhibition alleviates CCl_4_-induced hepatic injury and inflammation. **Fig. S24** Targeting GPR65 alleviates hepatic fibrosis by suppressing the JNK/NF-κB pathways.

**Additional file 2:** The GPCRs gene set.

## Data Availability

All the data supporting the findings of this study are available within the article and its Supplementary Information files or from the corresponding author upon reasonable request.

## Abbreviations

BDL: Bile duct ligation
BMM: Bone marrow-derived macrophage
BMT: Bone marrow transplantation
cAMP: Cyclic AMP
CCl_4_: Carbon tetrachloride
CM: Conditioned medium
CREB: CAMP-responsive-element-binding protein
ECM: Extracellular matrix
ELISA: Enzyme-linked immunosorbent assay
FBS: Fetal bovine serum
GO: Gene Ontology
GPCR: G-protein coupled receptor
HC: Hepatocyte
HEPES: 4-(2-Hydroxyethyl)-1-piperazineethanesulfonic acid
HM: Hepatic macrophage
HSC: Hepatic stellate cell
IHC: Immunohistochemistry
KC: Kupffer cells
KEGG: Kyoto Encyclopedia of Genes and Genomes
KO: Knockout
LSEC: Liver sinusoidal endothelial cell
PKA: Protein kinase A
PKC: Protein kinase C
PLC-β: Phospholipase C-β
qRT-PCR: Quantitative real-time reverse transcription-polymerase chain reaction
