# Supplementary material for: Targeting GPR65 alleviates hepatic inflammation and fibrosis by suppressing the JNK and NF-κB pathways
Source: Mil Med Res. 2023 Nov 25;10:56. doi: 10.1186/s40779-023-00494-4 (PMC10675918; doi:10.1186/s40779-023-00494-4)
Supplement: Supplementary file 1 — Additional file 1: Materials and methods. Table S1 Clinical characteristics of patients. Table S2 Quantitative real-time reverse transcription-polymerase chain reaction (qRT-PCR) primers for analysis of transcript levels. Table S3 Cloning primers for GPR65. Table S4 siRNA sequences. Fig. S1 Identification of GPR65 during hepatic fibrosis. Fig. S2 GPR65 is enriched in hepatic macrophage. Fig. S3 Gpr65 deficiency alleviates BDL-induced hepatic fibrosis. Fig. S4 Gpr65 deficiency alleviates BDL-induced hepatic injury and inflammation. Fig. S5 Gpr65 deficiency ameliorates CCl4-induced hepatic injury and inflammation. Fig. S6 Gpr65 deficiency in BMMs ameliorates CCl4-induced hepatic fibrosis, injury and inflammation. Fig. S7 GPR65 promotes BMMs M1 polarization. Fig. S8 GPR65 promotes RAW264.7 cell M1 polarization. Fig. S9 Extracellular pH differentially regulates HMs polarization partly via GPR65. Fig. S10 Extracellular pH differentially regulates BMMs polarization partly via GPR65. Fig. S11 Extracellular pH differentially regulates RAW264.7 cells polarization partly via GPR65. Fig. S12 Effect of GPR65 exogenous agonist and inhibitor on HMs polarization. Fig. S13 Effect of GPR65 exogenous agonist and inhibitor on BMMs polarization. Fig. S14 Effect of GPR65 exogenous agonist and inhibitor on RAW264.7 cells polarization. Fig. S15 GPR65 is not involved in regulation of HSCs activation directly. Fig. S16 GPR65 promotes the expression and release of TGF-β. Fig. S17 GPR65 aggravates HCs damage through the signals derived from macrophage. Fig. S18 Targeting GPR65 alleviates inflammation via Gαq. Fig. S19 Targeting GPR65 alleviates hepatic macrophage inflammation via Gαq. Fig. S20 Targeting GPR65 alleviates hepatic macrophage inflammation via Ca2+ rather than AMPK. Fig. S21 Targeting GPR65 alleviates hepatic macrophage inflammation by suppressing the JNK/NF-κB pathways. Fig. S22 Targeting GPR65 alleviates inflammation by suppressing the JNK/NF-κB pathways. Fig. S23 Pharmacologica [file 40779_2023_494_MOESM1_ESM.pdf]

Materials and methods

Immunohistochemistry-frozen (IHC-frozen)

Livers were embedded in O.C.T. compound (SAKURA Tissue-Tek®, Japan, Cat# 4583). Frozen sections (7 μm) were fixed in 4% paraformaldehyde (Solarbio, China, Cat# P1110), permeabilized with 0.1% Triton X-100, blocked using normal goat serum (ZSGB-BIO, China, Cat# ZLI-9021), and then incubated with GPR65 (NBP2-24487 for human; rabbit polyclonal; 1:100; AVIVA Systems Biology, USA, for mouse, OABF01813; rabbit polyclonal; 1:100) and LY6C (Novus, USA, NBP2-00441; rat monoclonal; 1:100) or F4/80 (Abcam, USA, ab16911; rat monoclonal; 1:50) or CD68 (Abcam, ab955; mouse monoclonal; 1:50) or α-SMA (Abcam, USA, ab7817; Mouse monoclonal; 1:50) overnight at 4° C. Sections were incubated with fluorescently conjugated secondary antibodies depending on host species (Alexa Fluor 594 goat anti-rat, Abcam, USA, ab150160; Alexa Fluor 594 goat anti-mouse, Abcam, USA, ab150116; Alexa Fluor 488 goat anti-rabbit, Abcam, USA, ab150077; 1:200). Co-stains were completed sequentially. Slides were stained with 10 μg/ml DAPI (Sigma, USA, Cat# B2883) and antifade solution was applied. Images were taken on an Olympus BX51 fluorescence microscope.

Liver enzyme measurement

Blood samples were collected and processed to serum. ALT and AST levels in serum were assessed using commercial assay kits (Nanjing Jian Cheng Biochemical Institute, Nanjing, China) according to the manufacturer's protocols.

Confocal microscopy

HSCs were plated on poly-lysine-pre-coated glass coverslips or macrophages were seeded in glass coverslips directly, then treated according to experimental conditions. Antibodies used for confocal microscopy included α-SMA (1:100, rabbit monoclonal, ABclonal, China, A17910), COL1α1 (1:100, rabbit polyclonal, Novus, USA, NB600-408), TNF-α (1:50, mouse monoclonal, Abcam, USA, ab1793) and an irrelevant isotype rabbit IgG. Cells were incubated with the proper fluorescent secondary antibody (Alexa Fluor 488/555/647, Invitrogen, USA) at 37 °C for 1 h away from light. DAPI (10 μg/ml, Sigma, USA, Cat# B2883) was used to stain the nuclei.

Plasmid construction and lentivirus production

The full-length human and mouse GPR65 cDNA were amplified and ligated into pcDNA3.1(+) and

pCCL.PPT.hPGK.IRES.eGFP/pre to obtain the overexpression plasmids (Lv-GPR65 or pcDNA3.1-GPR65). The empty plasmid was used as control. These plasmids together with the packaging plasmids pRSV-REV, pMDLg/pRRE and pMD2.BSBG were used to produce lentivirus in HEK-293T cells. The viruses were harvested at 36 h and 60 h post-transfection and filtered via 0.45 μm PVDF filters. The primers are shown in **Additional file 1: Table S3**.

Cell transfection

Negative control vectors and pcDNA3.1-GPR65 were transfected into cells using Lipofectamine 2000 (Invitrogen, USA, Cat# 11668019). Non-targeting siRNA (NC) and siRNAs targeting GPR65, Gαi2, Gαi3, Gαq, Gα11, Gα13 were obtained from GenePharma Biological Technology (Shanghai, China). Cells were transfected with siRNAs at 40% confluence using lipofectamine MAX (Invitrogen, USA, Cat# 13778150). After 24 – 48 h of culture, the knockdown and overexpression efficiency were analyzed by real-time PCR. The siRNAs sequences are shown in **Additional file 1: Table S4**.

ELISA

Levels of TNF-α (Thermo Fisher, USA, #BMS607-3), TGF-β1 (Abcam, USA, ab119557) and IL-6 (Thermo Fisher, USA, #BMS603-2) in cell culture supernatant were assessed according to the manufacturer's protocols.

Cell viability (Cell Counting Kit-8 assay)

2×10^3 RAW264.7 cells were incubated in 96-well plates and transfected with NC, GPR65-siRNA-1#, GPR65-siRNA-2#, pcDNA3.1 or pcDNA3.1-GPR65 for 48 and 72 h. WST-8 (10 μl) were added to each well. Absorbance of the formazan product was measured at 450 nm.

Hoechst/PI double staining

Apoptosis was determined by Hoechst 33342/PI double staining using commercial kits (Solarbio, China, Cat# CA1120) according to the manufacturer's instructions.

Table S1 Clinical characteristics of patients

METAVIR score	Normal (F0, *n* = 6)	Fibrosis (*n* = 28)			
		Mild fibrosis		Advanced fibrosis	
		F1	F2	F3	F4
Cases (*n*)	6	7	9	6	6
Age (years, means ± SD)	57.7 ± 15.7	52.4 ± 9.9			
Sex [male, *n*(%)]	4 (66.7)	14 (50)			
ALT (U/L, means ± SD)	22.0 ± 12.3	33.87 ± 25.8			
AST (U/L, means ± SD)	26.5 ± 12.6	51.3 ± 64.5			
GGT (U/L, means ± SD)	47.3 ± 25.0	94.1 ± 72.6			
Etiology [*n*(%)]					
Biliary obstruction	0	2 (7.1)			
Hepatitis B virus	0	25 (89.3)			
Hepatitis C virus	0	1 (3.6)			

ALT alanine aminotransferase, *AST* aspartate aminotransferase, *GGT* γ-glutamyl transpeptidase

Table S2 Quantitative real-time reverse transcription-polymerase chain reaction (qRT-PCR) primers for analysis of transcript levels

Source	Gene symbol	Forward (5' – 3')	Reverse (5' – 3')
Mouse	*Gapdh*	GGCATGGACTGTGGTCATGAG	TGCACCACCAACTGCTTAGC
	Gpr65	CTGGACCGCTATTTAGCAGTC	GCTGGTAATAAACGCGAATCTTC
	Acta2	TCGGATACTTCAGCGTCAGGA	GTCCCAGACATCAGGGAGTAA
	Col1α1	ATCGGTCATGCTCTCTCCAAACA	ACTGCAACATGGAGACAGGTCAGA
	Col3α1	GAGAAAGGTGAAGGAGGCCC	CAGCTATTCCAGGGTTGCCA
	Col1α2	TGGCAGAGCTGGTGTAATGG	TAGGACCTCGGATTCCAGCA
	Col12α1	CAGGAGTCACAGGGCCAAG	GCGATGTCTCCCCTATCACC
	Timp1	TCCGTCCACAAACAGTGAGTGTCA	GGTGTGCACAGTGTTTCCCTGTTT
	Mmp2	GTGTTCTTCGCAGGGAATGAG	GATGCTTCCAAACTTCACGCT
	Tgfβ1	GGACTCTCCACCTGCAAGAC	CATAGATGGCGTTGTTGCGG
	Tgfβ2	TTTTGCTCCAGACAGTCCCA	TCCAGTCTGTAGGAGGGCAA
	Tgfβ3	GGACTTCGGCCACATCAAGAA	TAGGGGACGTGGGTCATCAC
	Pdgfβ	CTGCCACAGCATGATGAGGAT	GCCAGGATGGCTGAGATCACCAC
	Bad	TAGAACTGGAGGGAGGAGGC	CCGTCCCTGCTGATGAATGT
	Bax	CTCAAGGCCCTGTGCACTAA	CACGGAGGAAGTCCAGTGTC
	Pcna	TTTGAGGCACGCCTGATCC	GGAGACGTGAGACGAGTCCAT
	Ki67	CATCCATCAGCCGGAGTCA	TGTTTCGCAACTTTCGTTTGTG
	Ctgf	AGAACTGTGTACGGAGCGTG	GTGCACCATCTTTGGCAGTG
	Acnat2	GACCTGCCTGAGAAACCACAA	GTGATGGTGGTGGGCCCATTTA
	Dhrs9	TGACTGGTTGACAGTGGACG	GAGTATAGCCCCCTCCACCA
	Nox2	CGGAGGGGCTATTCAATGCT	CACTGGCTGTACCAAAGGGT
	Gpcpd1	CTGTGATGCCCTGGGAAACT	TGACTTGACATGGACCACCG
	Gsta2	GCCTGCCTTTGAAAAGGTGT	ATAGAGAAGAAGTTCCAGCAGGTG
	Ugt2β1	TGGCTCGTTCGAACCTTCTG	TCCTTAGGCAGTGGTTTGGC
	Sult1β1	TGTGGCCTATGGTTCATGGT	GGTGTGATGGACGATCCTGT
	Cyp2α5	CCCACCTTCTACCTTAGCCG	CTTCCCAGCATCATTCGAAGC
	Cyp4α12	AGTGTCCTCTAATGGCTGCTT	TGTCCAGGAAATCCAATCGCC
	Slc25α1	CCTCAGCTCCTTGCTCTACG	CCATAGGGCACACGACTACC

Source	Gene symbol	Forward (5’ – 3’)	Reverse (5’ – 3’)
	Il1β	GTCGCTCAGGGTCACAAGAA	GTG-CTGCCTAATGTCCCCTT
	Ly6c	GCAGTGCTACGAGTGCTATGG	ACTGACGGGTCTTTAGTTTCCTT
	Adgre1	TGACTCACCTTGTGGTCCTAA	CTTCCCAGAATCCAGTCTTTCC
	Il6	AGTTGCCTTCTTGGGACTGA	TCCACGATTTCCCAGAGAAC
	Tnfα	CATCTTCTCAAAATTCGAGTGACAA	TGGGAGTAGACAAGGTACAACCC
	Ccl2	GTTAACGCCCCACTCACCTG	GGGCCGGGGTATGTAACTCA
	Ccr2	ATGCAAGTTCAGCTGCCTGC	ATGCCGTGGATGAACTGAGG
	Ccl5	CCACTTCTTCTCTGGGTTGG	GTGCCCACGTCAAGGAGTAT
	Nos2	CAGGGCCACCTCTACATTTG	TGCCCCATAGGAAAAGACTG
	Cxcl10	GATGACGGGCCAGTGAGAAT	CTCAACACGTGGGCAGGATA
	Cd80	ACCCCCAACATAACTGAGTCT	TTCCAACCAAGAGAAGCGAGG
	Cd86	CTGGACTCTACGACTTCACAATG	AGTTGGCGATCACTGACAGTT
	Mrc1	GTGGAGTGATGGAACCCCAG	CTGTCCGCCCAGTATCCATC
	Arg1	ACATTGGCTTGCGAGACGTA	ATCACCTTGCCAATCCCCAG
	Cd163	TGTGCAGTAACGGCTGGAG	ATCATGTTTGCAGTCCCAAAGA
	Il10	GCTCTTGCACTACCAAAGCC	CTGCTGATCCTCATGCCAGT
	Tlr4	TTTGCTGGGGCTCATTCACT	GACTCGGCACTTAGCACTGT
	Gαi1	GAAATGAACCGTATGCACGAGA	TGGACGTGTCTGTAAACCACT
	Gαi2	AATAAGCGCAAAGACACCAAGG	AGTGACGGCATCGAACACAAA
	Gαi3	TGAGGACGGCTATTCAGAGGA	CGTTTAATCACGCCTGCTAGTTC
	Gαq	GGTCGGGCTACTCTGACGA	ACTTGTATGGGATCTTGAGCGT
	Gα11	CAACGCGGAGATCGAGAAACA	GCCTGCATGGCGGTAAAGAT
	Gα13	CGGAAACGCTGGTTTGAATGC	AGATTCTGTAAGGCGATTGGTCT
	Gαs	CAGAGCCTCCATTGGGGTC	GCTTCTCGCTCAACTGGGG
	Gpr4	GCTGGGCGTCTACCTGATG	AGGCGATGCTGATATAGATGTTG
	Gpr68	TATCTTGCCCCATCGACCACA	AGTACCCGAAGTAGAGGGACA
	Gpr132	CCTGCAAGGTGACTGCTTACA	GAAGAATCACACACGCAGAAATG
Human	*GAPDH*	ACCCAGAAGACTGTGGATGG	TTCAGCTCAGGGATGACCTT
	GPR65	GCCGTTGATCGGTATTTGGC	AGGCTGACCATGAGTGCAAA
	ACTA2	GCCATGTTCTATCGGGTACTTC	CAGGGCTGTTTTCCCATCCAT

Source	Gene symbol	Forward (5' – 3')	Reverse (5' – 3')
	COL1α1	AACCAAGGCTGCAACCTGGA	GGCTGAGTAGGGTACACGCAGG
	COL1α2	GCAGGAGGTTTCGGCTAAGT	GCAACAAAGTCCGCGTATCC
	TIMP1	GGGGCTTCACCAAGACCTAC	GGAAGCCCTTTTCAGAGCCT
	MMP2	GTGTTCTTCGCAGGGAATGAG	GATGCTTCCAAACTTCACGCT
	TGFβ1	TGTTGGACAGCTGCTCCACCT	GGCAGTGGTTGAGCCGTGGA

Table S3 Cloning primers for GPR65

**Name**	**Sequence (5' – 3')**
Mouse *Gpr65* BamH1 F	cgcggatccATGGCGATGAACAGCATGTG
Mouse *Gpr65* BamH1 R	cgcggatccTGCCTCCACCTCTTAGTCTAT
Mouse *Gpr65* Xho1 R	ccgctcgagTGCCTCCACCTCTTAGTCTAT
Human *GPR65* BamH1 F	cgcggatccAGACTTCTCTGTTTACTTTCT
Human *GPR65* BamH1 R	cgcggatccCTTCCCTTCAAAACATCCTTG

Table S4 siRNA sequences

**Name**	**Forward (5' – 3')**	**Reverse (5' – 3')**
Gpr65-siRNA-1	GCGCCACCUCGAACACUAUTT	AUAGUGUUCGAGGUGGCGCTT
Gpr65-siRNA-2	CCUGUCAGACCUGCUGUAUTT	AUACAGCAGGUCUGACAGGTT
Gαi2	CCUUGAGCGCUUAUGACUUTT	AAGUCAUAAGCGCUCAAGGTT
Gαi3	GGGACGGUUGAAGAUUGAUTT	AUCAAUCUUCAACCGUCCCTT
Gαq	CCACAGAUACCGAGAACAUTT	AUGUUCUCGGUAUCUGUGGTT
Gα11	GGUGGCACUAAGCGAGUAUTT	AUACUCGCUUAGUGCCACCTT
Gα13	GCCAGAGAUCAGAACGGAATT	UUCCGUUCUGAUCUCUGGCTT
Negative control	UUCUCCGAACGUGUCACGUTT	ACGUGACACGUUCGGAGAATT

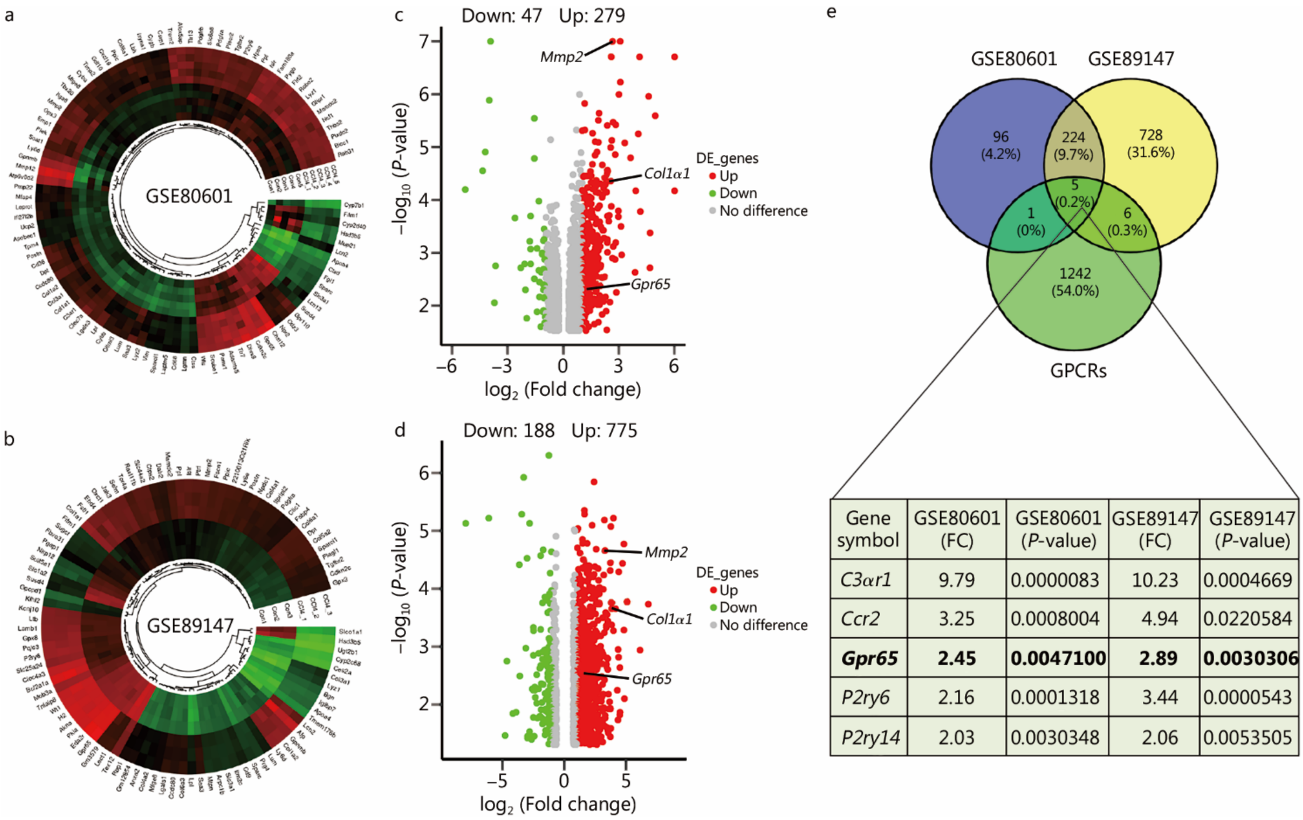

Gene symbol	GSE80601 (FC)	GSE80601 (*P*-value)	GSE89147 (FC)	GSE89147 (*P*-value)
C3αr1	9.79	0.0000083	10.23	0.0004669
Ccr2	3.25	0.0008004	4.94	0.0220584
Gpr65	**2.45**	**0.0047100**	**2.89**	**0.0030306**
P2ry6	2.16	0.0001318	3.44	0.0000543
P2ry14	2.03	0.0030348	2.06	0.0053505

Fig. S1 Identification of GPR65 during hepatic fibrosis, related to **Fig. 1**. Hierarchical cluster (**a** and **b**) and volcano map (**c** and **d)** analysis of differentially expressed mRNAs during hepatic fibrosis, acquired from our previous data: GSE80601 (normal liver tissues and CCl_4-induced hepatic fibrotic tissues, $n = 5$) and GSE89147 (normal liver tissues and CCl_4-induced hepatic fibrotic tissues, $n = 3$). Bright red, up-regulation; bright green, down-regulation. **e** The Venn diagram of differentially expressed mRNAs in GSE80601 and GSE89147, as well as the GPCRs gene set (**Additional file 2**). CCl_4 carbon tetrachloride, GPCR G-protein coupled receptor, FC fold change

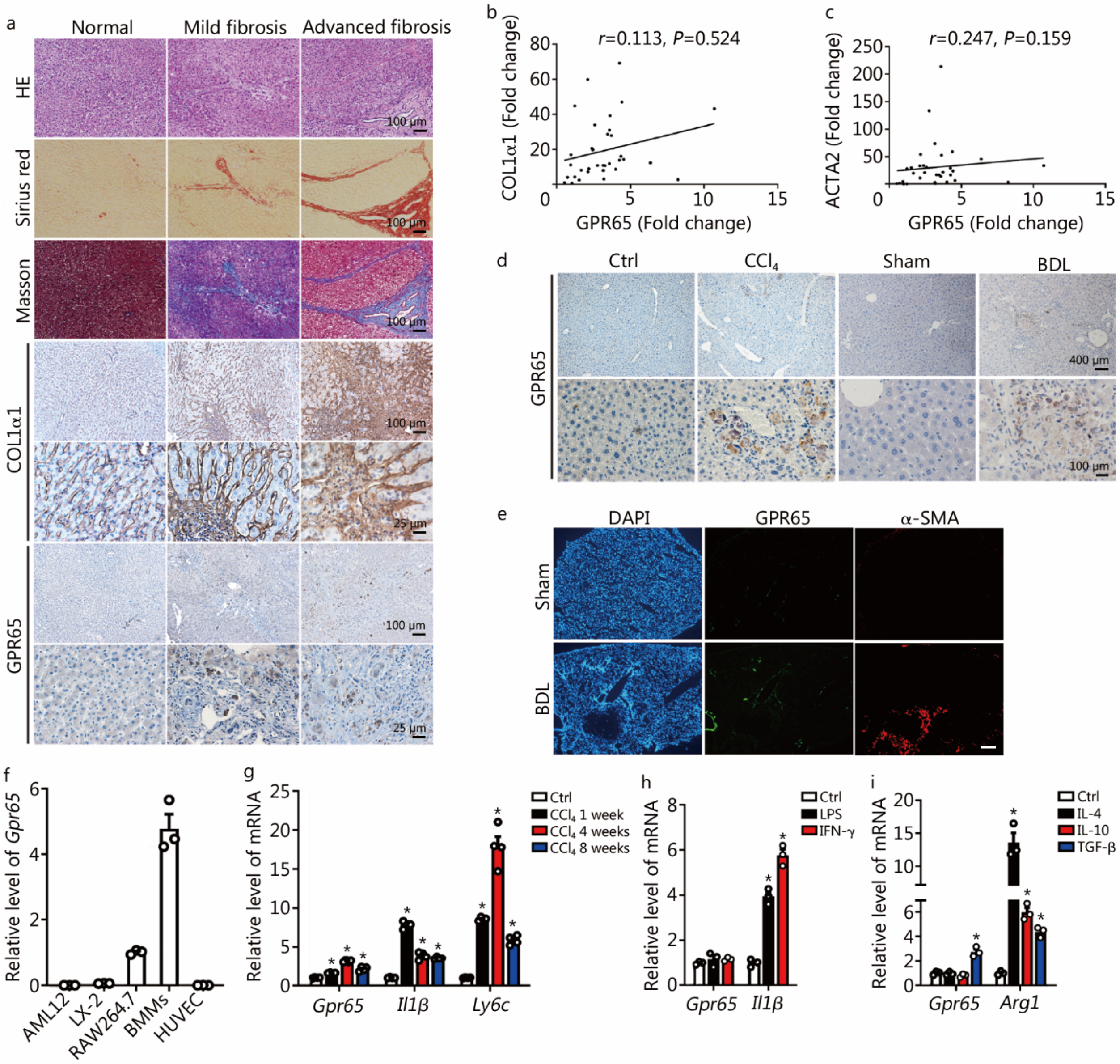

Fig. S2 GPR65 is enriched in hepatic macrophage, related to **Fig. 1**. **a** HE staining, Sirius red staining, Masson's trichrome staining, COL1α1 and GPR65 IHC staining in the livers of human normal controls, mild fibrosis and advanced fibrosis. Scale bar = 25 μm for 40× and 100 μm for 10×. The correlations of GPR65 with COL1α1 **(b)** and ACTA2 **(c)** were assessed using Pearson correlation analysis, *n* = 34. **d** IHC for GPR65 in the livers of mice treated with or without CCl_4 for 8 weeks or BDL for 21 d. Scale bar = 100 μm for 40× and 400 μm for 10×. **e** Representative IHC-frozen for co-staining GPR65 with α-SMA in the livers of mice with or without BDL for 21 d. Scale bar = 125 μm. **f** qRT-PCR was used to assess the expression of *Gpr65* in AML12, LX-2, RAW264.7, BMMs and HUVEC cells (*n* = 3). **g** qRT-PCR was used to assess the expression of *Gpr65*, *Il1β* and *Ly6c* in HMs isolated from mice treated with CCl_4 for 1, 4 or 8 weeks (*n* = 4). **h** qRT-PCR was used to assess the expression of *Gpr65* and *Il1β* (positive control gene) in HMs treated with 100 ng/ml LPS or 20 ng/ml IFN-γ for 24 h (*n* = 3). **i** qRT-PCR was used to assess the expression of *Gpr65* and *Arg1* (positive control gene) in HMs treated with 20 ng/ml IL-4 or 20 ng/ml IL-10 or 10 ng/ml TGF-β for 24 h (*n* = 3). $^*P < 0.05$ vs. Ctrl. BDL bile duct ligation, BMM bone marrow-derived macrophage, CCl_4 carbon tetrachloride, HM hepatic macrophage, IHC immunohistochemistry, qRT-PCR quantitative real-time reverse transcription-polymerase chain reaction, COL1α1 collagen type I alpha 1, ACTA2 actin alpha 2, smooth muscle, LPS lipopolysaccharide, IFN-γ interferon-γ, IL-4 interleukin-4, TGF-β transforming growth factor-β

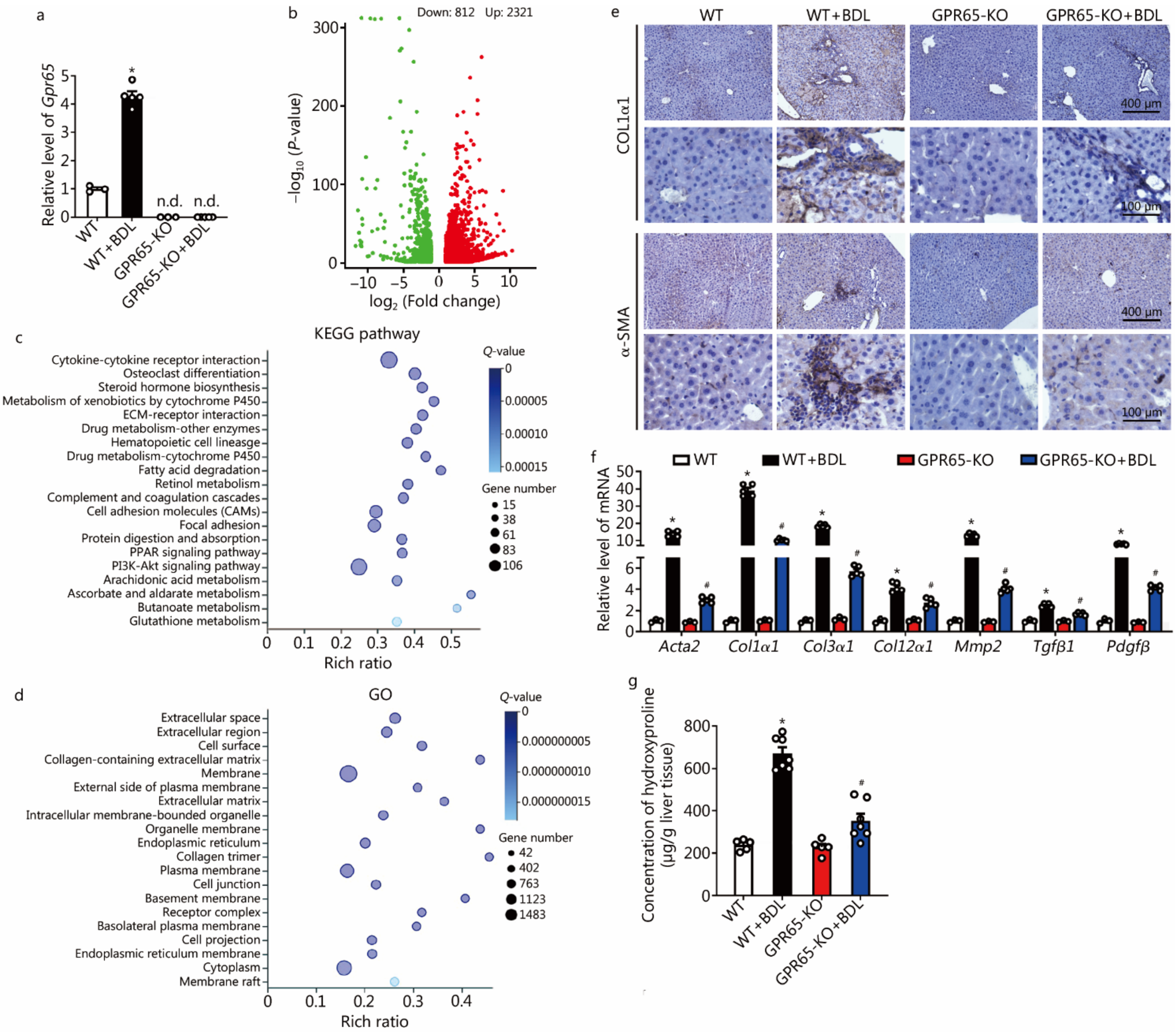

Fig. S3 *Gpr65* deficiency alleviates BDL-induced hepatic fibrosis, related to **Fig. 2**. **a** qRT-PCR was used to assess the expression of *Gpr65* in liver tissues from WT, WT + BDL, GPR65-KO and GPR65-KO + BDL mice ($n = 3, 5, 3, 5$). The volcano map analysis (**b**), KEGG (**c**) and GO (**d**) analysis of differentially expressed mRNAs in WT mice treated with BDL, compared to WT mice. **e** IHC for COL1α1 and α-SMA. Scale bar = 100 μm for 40× and 400 μm for 10×. **f** qRT-PCR was used to assess the mRNA level of *Acta2*, *Col1α1*, *Col3α1*, *Col12α1*, *Mmp2*, *Tgfβ1* and *Pdgfβ* ($n = 3, 5, 3, 5$). **g** The content of hepatic hydroxyproline was quantified in livers ($n = 5, 7, 5, 7$). $^{*}P < 0.05$ vs. WT; $^{\#}P < 0.05$ vs. WT + BDL. BDL bile duct ligation, GO Gene Ontology, IHC immunohistochemistry, KO knockout, KEGG Kyoto Encyclopedia of Genes and Genomes, qRT-PCR quantitative real-time reverse transcription-polymerase chain reaction, COL1α1 collagen type I alpha 1, α-SMA alpha-smooth muscle actin

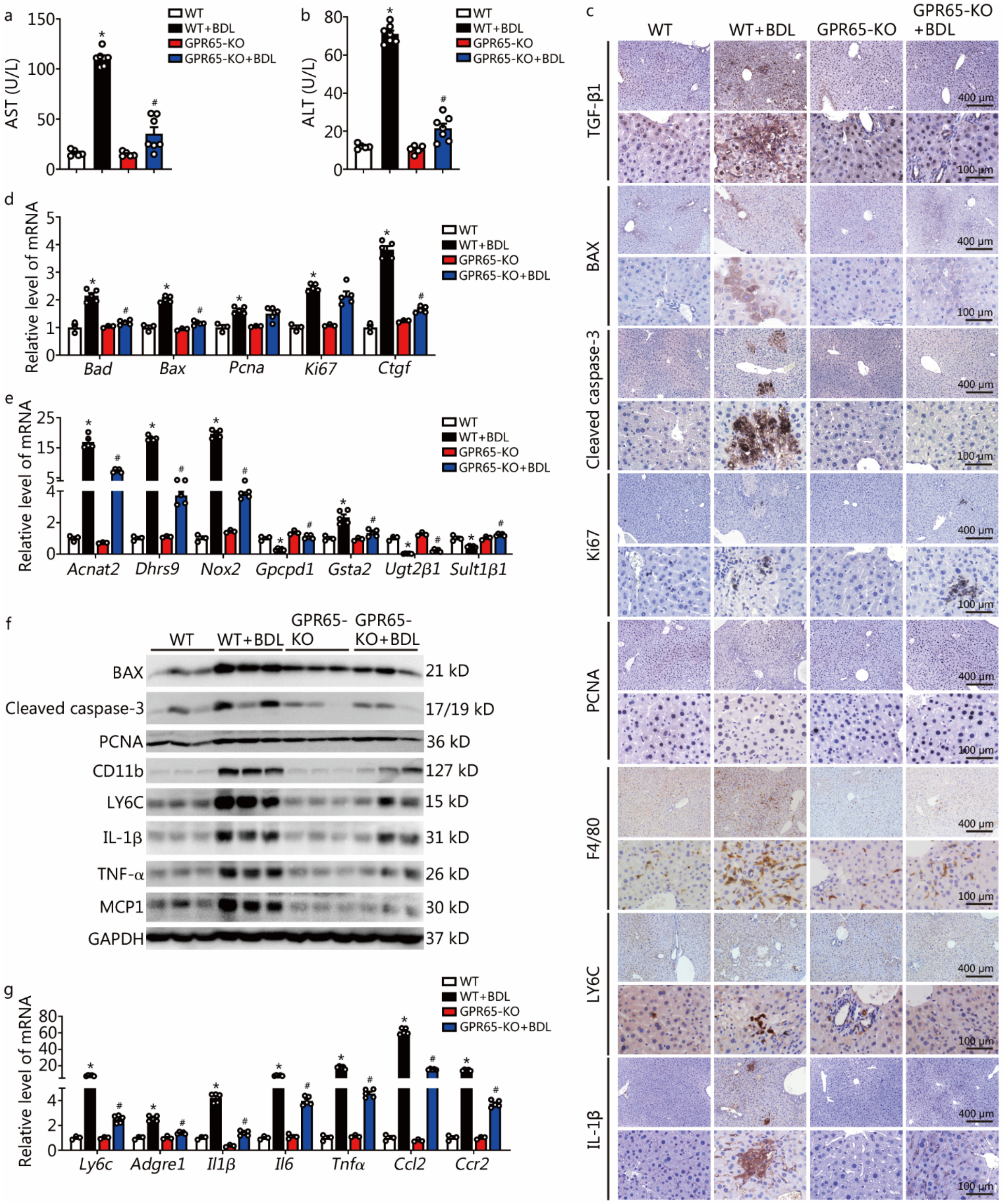

Fig. S4 *Gpr65* deficiency alleviates BDL-induced hepatic injury and inflammation, related to **Fig. 2**. WT and GPR65-KO mice were divided into 4 groups: WT, WT + BDL, GPR65-KO and GPR65-KO + BDL. Serum AST (**a**) and ALT (**b**) were examined (*n* = 5, 7, 5, 7). **c** IHC for TGF-β1, BAX, cleaved caspase-3, Ki67, PCNA, F4/80, LY6C and IL-1β. Scale bar = 100 μm for 40× and 400 μm for 10×. **d** qRT-PCR was used to assess the mRNA level of *Bad, Bax, Pcna, Ki67* and *Ctgf* (*n* = 3, 5, 3, 5). **e** qRT-PCR was used to assess the mRNA level of *Acnat2, Dhrs9, Nox2, Gpcpd1, Gsta2, Ugt2β1* and *Sult1β1* (*n* = 3, 5, 3, 5). **f** Western blotting was used to determine the protein level of BAX, cleaved caspase-3, PCNA, CD11b, LY6C, IL-1β, TNF-α and MCP1. **g** qRT-PCR was used to assess the mRNA level of *Ly6c*, *Adgre1*, *Il1β*, *Il6*, *Tnfα*, *Ccl2* and *Ccr2* (*n* = 3, 5, 3, 5). $^{*}P < 0.05$ vs. WT; $^{\#}P < 0.05$ vs. WT + BDL. BDL bile duct

ligation, IHC immunohistochemistry, KO knockout, qRT-PCR quantitative real-time reverse transcription-polymerase chain reaction, AST aspartate transaminase, ALT alanine transaminase, TGF-β1 transforming growth factor-β1, BAX bcl2-associated X protein, PCNA proliferating cell nuclear antigen, LY6C lymphocyte antigen 6 complex, IL-1β interleukin-1β, TNF-α tumor necrosis factor-α, MCP1 monocyte chemoattractant protein 1

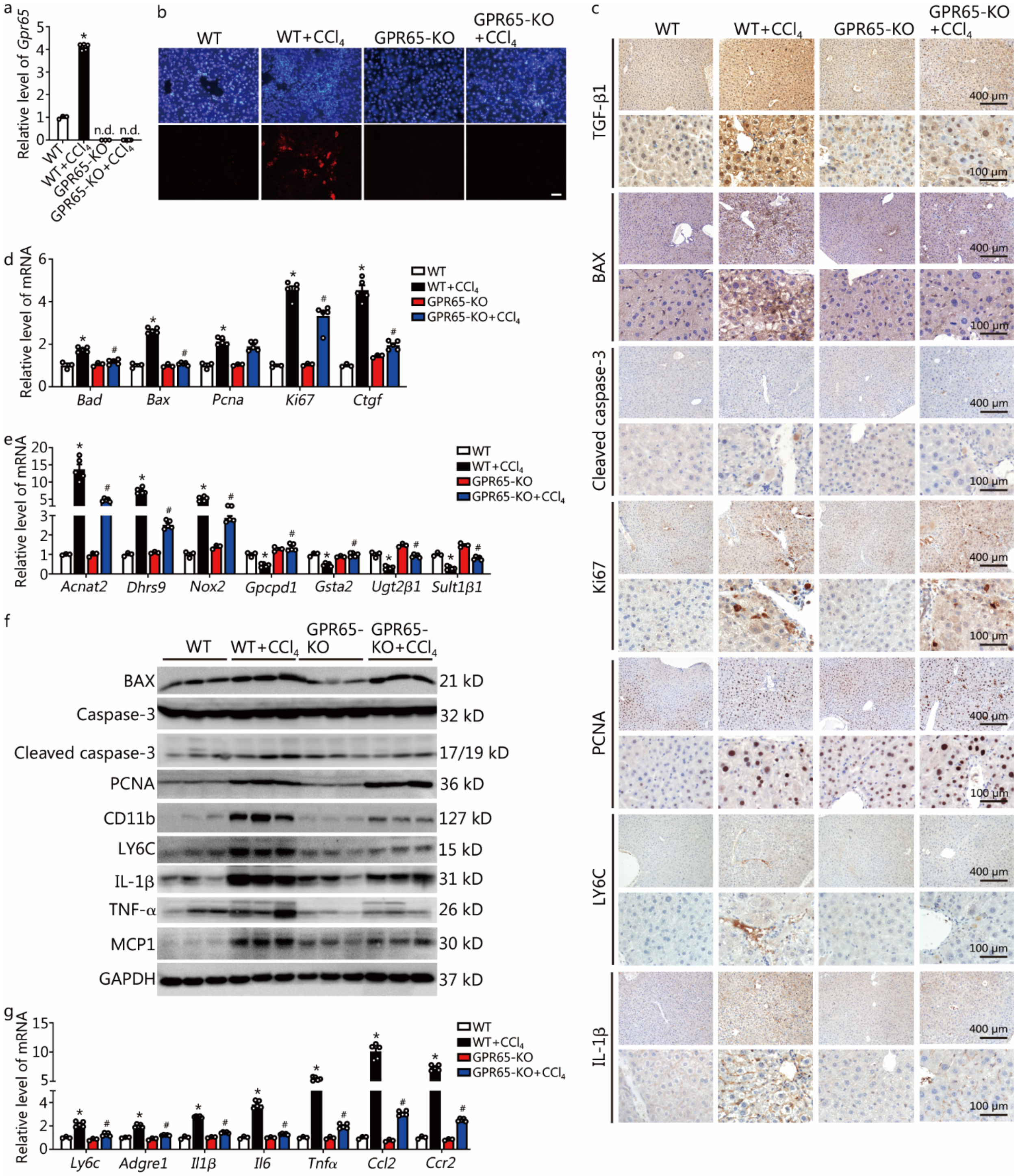

Fig. S5 *Gpr65* deficiency ameliorates CCl_4-induced hepatic injury and inflammation, related to **Fig. 3**. **a** WT and GPR65-KO mice were divided into 4 groups: WT, WT + CCl_4, GPR65-KO and GPR65-KO + CCl_4. qRT-PCR was used to assess the expression of *Gpr65* in liver tissues (n = 3, 5, 3, 5). **b** IHC-frozen staining for GPR65. Scale bar = 50 μm. **c** IHC for TGF-β1, BAX, cleaved caspase-3, Ki67, PCNA, LY6C and IL-1β. Scale bar = 100 μm for 40× and 400 μm for 10×. **d** qRT-PCR was used to assess the mRNA level of *Bad, Bax, Pcna, Ki67* and *Ctgf* (n = 3, 5, 3, 5). **e** qRT-PCR was used to assess the mRNA level of *Acnat2*, *Dhrs9*, *Nox2*, *Gpcpd1*, *Gsta2*, *Ugt2β1* and *Sult1β1* (n = 3, 5, 3, 5). **f** Western blotting was used to determine the protein level of BAX, cleaved caspase-3, PCNA, CD11b, LY6C, IL-1β, TNF-α and MCP1. **g** qRT-PCR was used to assess the mRNA level of *Ly6c*, *Adgre1*, *Il1β*, *Il6*, *Tnfα*, *Ccl2* and *Ccr2* (n = 3, 5, 3, 5). $^{*}P < 0.05$ vs. WT; $^{\#}P < 0.05$ vs. WT + CCl_4. CCl_4 carbon tetrachloride, IHC

immunohistochemistry, KO knockout, qRT-PCR quantitative real-time reverse transcription-polymerase chain reaction, TGF-β1 transforming growth factor-β1, BAX bcl2-associated X protein, PCNA proliferating cell nuclear antigen, LY6C lymphocyte antigen 6 complex, IL-1β interleukin-1β, TNF-α tumor necrosis factor-α, MCP1 monocyte chemoattractant protein 1

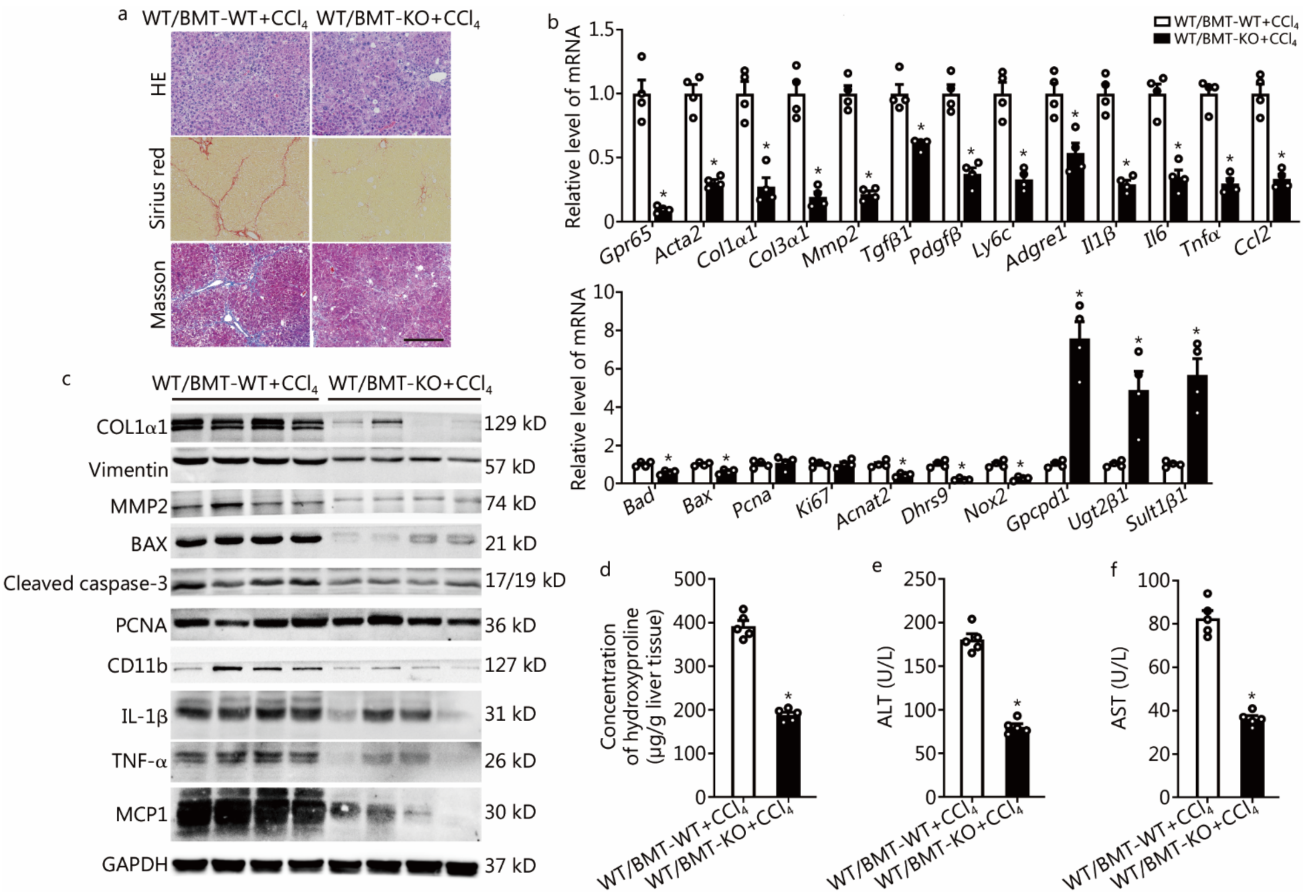

Fig. S6 *Gpr65* deficiency in BMMs ameliorates CCl_4-induced hepatic fibrosis, injury and inflammation, related to **Fig. 3**. **a** Male WT recipient mice were lethal irradiation and then intravenous injection of 1×10^7 bone marrow cells harvested from WT or GPR65-KO donor mice. Four weeks after BMT, the chimeric mice were treated with CCl_4 thrice a week for 6 weeks. Hepatic fibrosis was evaluated by HE staining, Sirius red staining and Masson's trichrome staining. Scale bar = 400 μm. **b** qRT-PCR was used to assess the mRNA level of *Gpr65*, *Acta2*, *Col1α1*, *Col3α1*, *Mmp2*, *Tgfβ1*, *Pdgfβ*, *Ly6c*, *Adgre1*, *Il1β*, *Il6*, *Tnfα*, *Ccl2*, *Bad*, *Bax*, *Pcna*, *Ki67*, *Acnat2*, *Dhrs9*, *Nox2*, *Gpcpd1*, *Ugt2β1* and *Sult1β1* ($n = 4$). **c** Western blotting was used to determine the protein level of COL1α1, vimentin, MMP2, BAX, cleaved caspase-3, PCNA, CD11b, IL-1β, TNF-α and MCP1. **d** The content of hepatic hydroxyproline was quantified in livers of each group ($n = 4$). Serum ALT (**e**) and AST (**f**) were examined ($n = 4$). $^*P < 0.05$ vs. WT/BMT-WT + CCl_4. BMT bone marrow transplantation, CCl_4 carbon tetrachloride, IHC immunohistochemistry, KO knockout, qRT-PCR quantitative real-time reverse transcription-polymerase chain reaction, BMM bone marrow-derived macrophages, COL1α1 collagen type I alpha 1, MMP2 matrix metallopeptidase 2, BAX bcl2-associated X protein, PCNA proliferating cell nuclear antigen, IL-1β interleukin-1β, TNF-α tumor necrosis factor-α, MCP1 monocyte chemoattractant protein 1, ALT alanine transaminase, AST aspartate transaminase

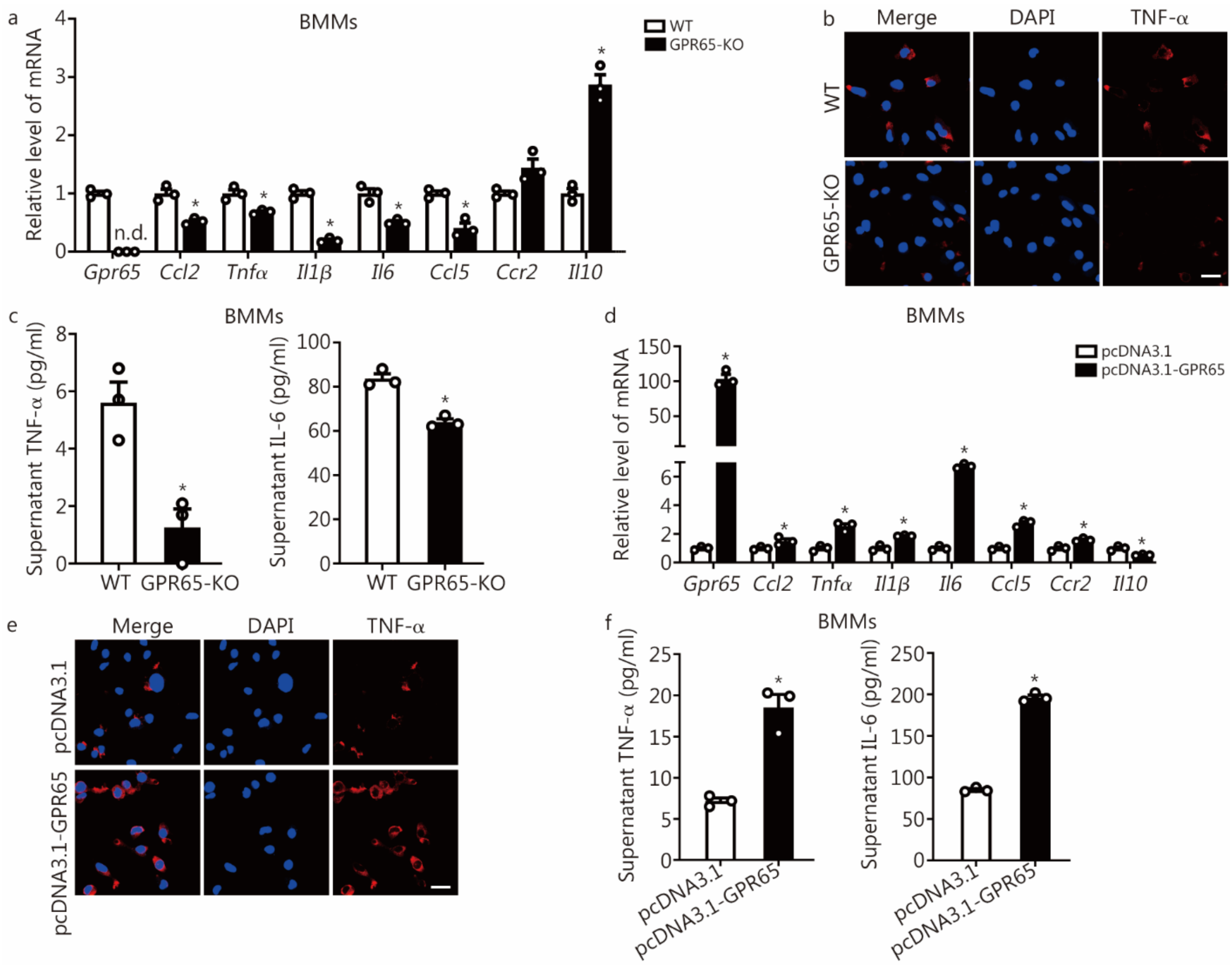

Fig. S7 GPR65 promotes BMMs M1 polarization, related to **Fig. 4**. **a** qRT-PCR was used to assess the mRNA level of *Gpr65*, *Ccl2*, *Tnfα*, *Il1β*, *Il6*, *Ccl5*, *Ccr2* and *Il10* in BMMs isolated from WT and GPR65-KO mice (n = 3). **b** The expression and location of TNF-α in BMMs isolated from WT and GPR65-KO mice were assessed by confocal microscopy. Scale bar = 20 μm. **c** TNF-α and IL-6 level in the supernatant of BMMs isolated from WT and GPR65-KO mice were detected by ELISA (n = 3). BMMs were transfected with pcDNA3.1 or pcDNA3.1-GPR65 for 48 h, qRT-PCR was used to assess the mRNA level of *Gpr65*, *Ccl2*, *Tnfα*, *Il1β*, *Il6*, *Ccl5*, *Ccr2* and *Il10* (n = 3, **d**); the expression and location of TNF-α was assessed by confocal microscopy (**e**). Scale bar = 20 μm. TNF-α and IL-6 level in the supernatant were detected by ELISA (n = 3, **f**). $^{*}P < 0.05$ vs. WT or pcDNA3.1. BMM bone marrow-derived macrophage, ELISA enzyme-linked immunosorbent assay, KO knockout, qRT-PCR quantitative real-time reverse transcription-polymerase chain reaction, TNF-α tumor necrosis factor-α, IL-6 interleukin-6

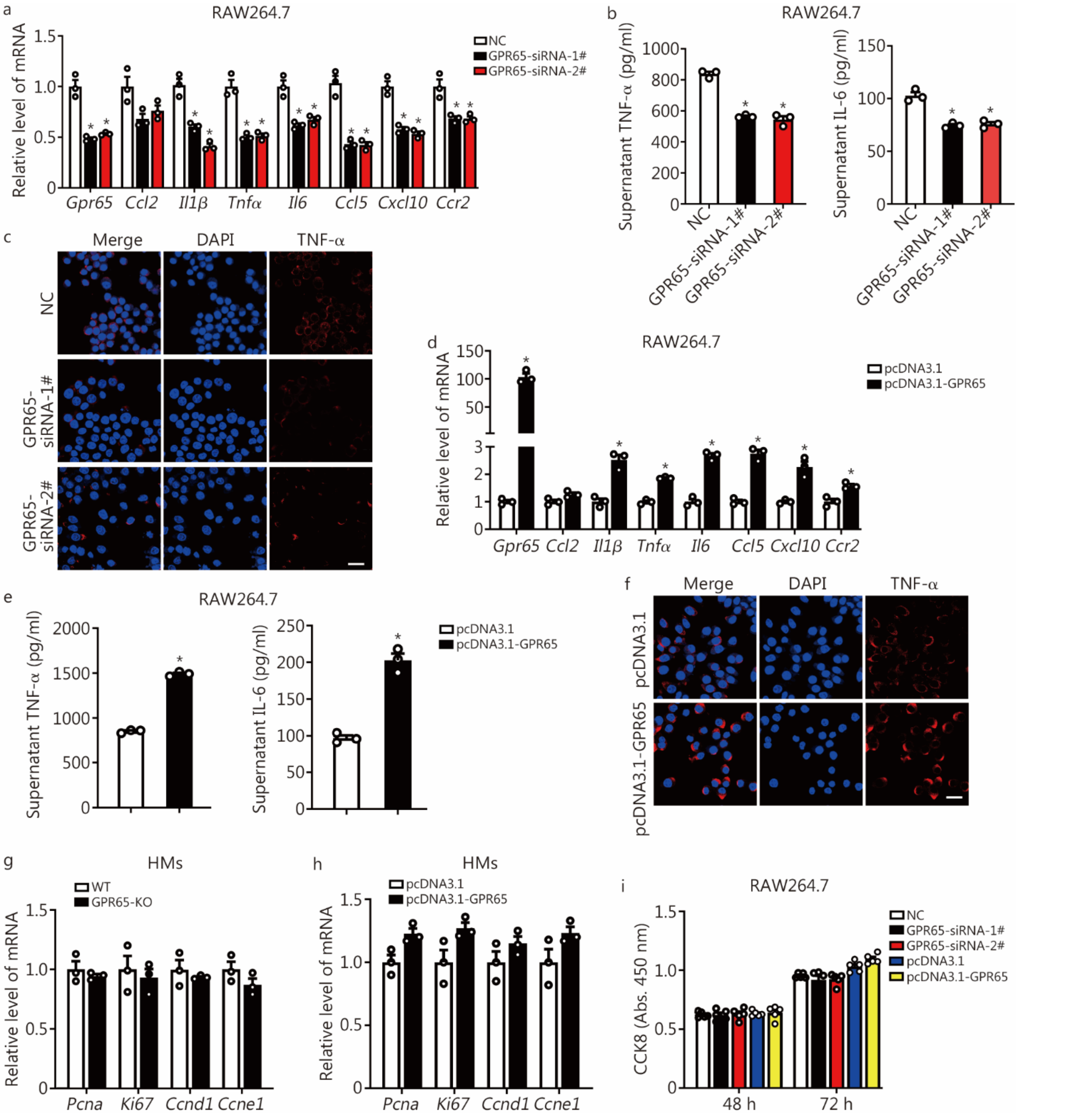

Fig. S8 GPR65 promotes RAW264.7 cell M1 polarization, related to **Fig. 4**. RAW264.7 cells were transfected with NC, GPR65-siRNA-1# or GPR65-siRNA-2# for 36 h, qRT-PCR was used to assess the mRNA level of *Gpr65*, *Ccl2*, *Il1β*, *Tnfα*, *Il6*, *Ccl5*, *Cxcl10* and *Ccr2* (n = 3, **a**); TNF-α and IL-6 level in the supernatant were detected by ELISA (n = 3, **b**); the expression and location of TNF-α was assessed by confocal microscopy (**c**). Scale bar = 20 μm. RAW264.7 cells were transfected with pcDNA3.1 or pcDNA3.1-GPR65 for 48 h, qRT-PCR was used to assess the mRNA level of *Gpr65*, *Ccl2*, *Il1β*, *Tnfα*, *Il6*, *Ccl5*, *Cxcl10* and *Ccr2* (n = 3, **d**); TNF-α and IL-6 level in the supernatant were detected by ELISA (n = 3, **e**); the expression and location of TNF-α was assessed by confocal microscopy (**f**). Scale bar = 20 μm. **g** qRT-PCR was used to assess the mRNA level of *Pcna*, *Ki67*, *Ccnd1* and *Ccne1* in HMs isolated from WT and GPR65-KO mice (n = 3). **h** HMs were transfected with pcDNA3.1 or pcDNA3.1-GPR65 for 48 h, qRT-PCR was used to assess the mRNA level of *Pcna, Ki67, Ccnd1* and *Ccne1* (n = 3). **i** RAW264.7 cells were transfected with NC, GPR65-siRNA-1#, GPR65-siRNA-2#, pcDNA3.1 or pcDNA3.1-GPR65 for 48 and 72 h, cell proliferation

was determined by CCK8 ($n = 3$). $^{*}P < 0.05$ vs. NC or pcDNA3.1. ELISA enzyme-linked immunosorbent assay, qRT-PCR quantitative real-time reverse transcription-polymerase chain reaction, Abs. absorbance, TNF-α tumor necrosis factor-α, IL-6 interleukin-6

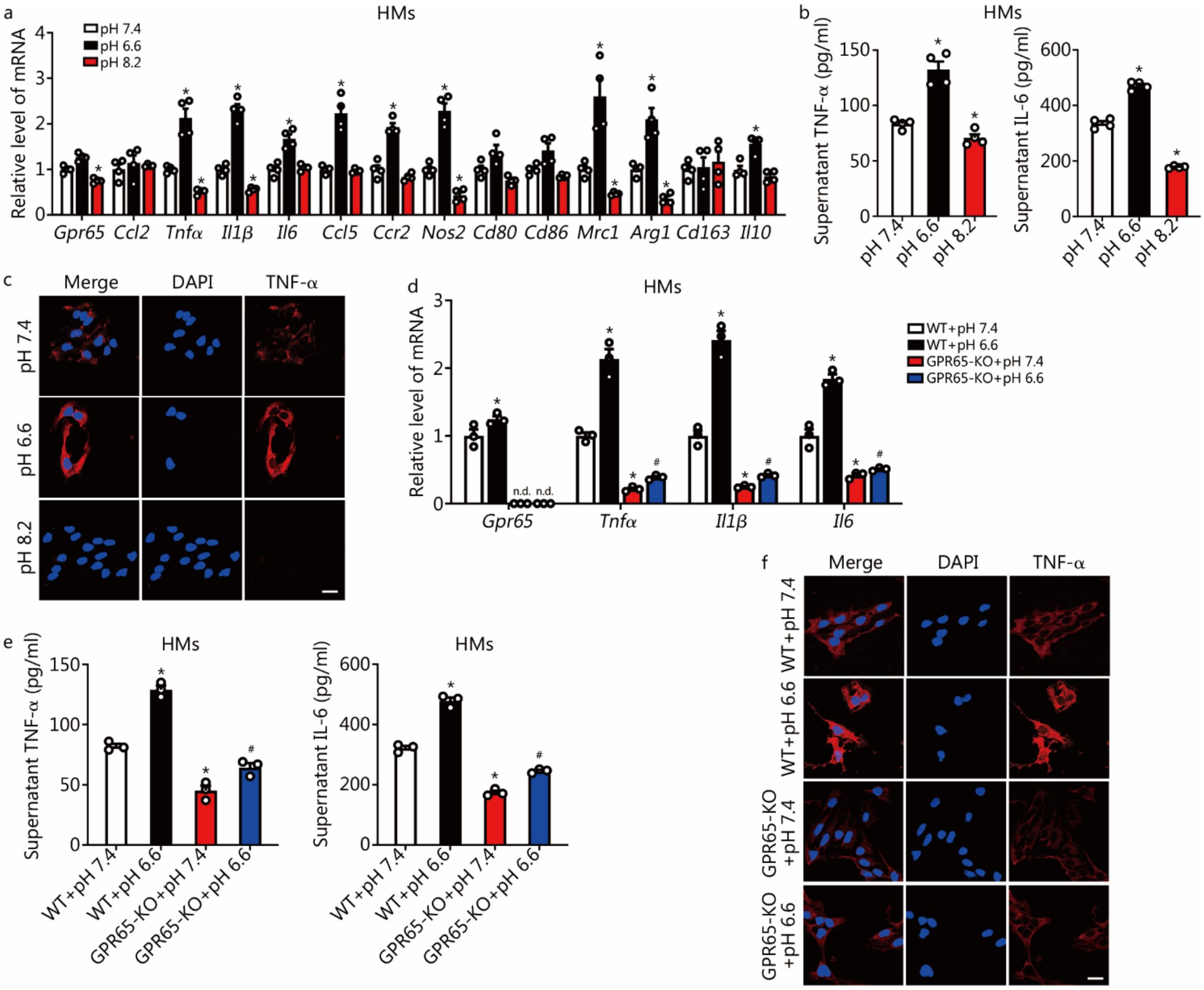

Fig. S9 Extracellular pH differentially regulates HMs polarization partly via GPR65. HMs were incubated in the physiological pH 7.4, the acidic pH 6.6 or the alkalic pH 8.2 for 24 h, qRT-PCR was used to assess the mRNA level of *Gpr65*, *Ccl2*, *Tnfa*, *Il1β*, *Il6*, *Ccl5*, *Ccr2*, *Nos2*, *Cd80*, *Cd86*, *Mrc1*, *Arg1*, *Cd163* and *Il10* ($n = 4$, **a**); TNF-α and IL-6 level in the supernatant were detected by ELISA ($n = 4$, **b**); the expression and location of TNF-α were assessed by confocal microscopy (**c**). Scale bar = 20 μm. HMs isolated from WT and GPR65-KO mice were incubated in pH 7.4 and pH 6.6 for 24 h, qRT-PCR was used to assess the mRNA level of *Gpr65*, *Tnfa*, *Il1β* and *Il6* ($n = 3$, **d**); TNF-α and IL-6 level in the supernatant were detected by ELISA ($n = 3$, **e**); the expression and location of TNF-α was assessed by confocal microscopy (**f**). Scale bar = 20 μm. $^{*}P < 0.05$ vs. pH 7.4 or WT + pH 7.4; $^{\#}P < 0.05$ vs. WT + pH 6.6. HM hepatic macrophage, ELISA enzyme-linked immunosorbent assay, KO knockout, qRT-PCR quantitative real-time reverse transcription-polymerase chain reaction, TNF-α tumor necrosis factor-α, IL-6 interleukin-6

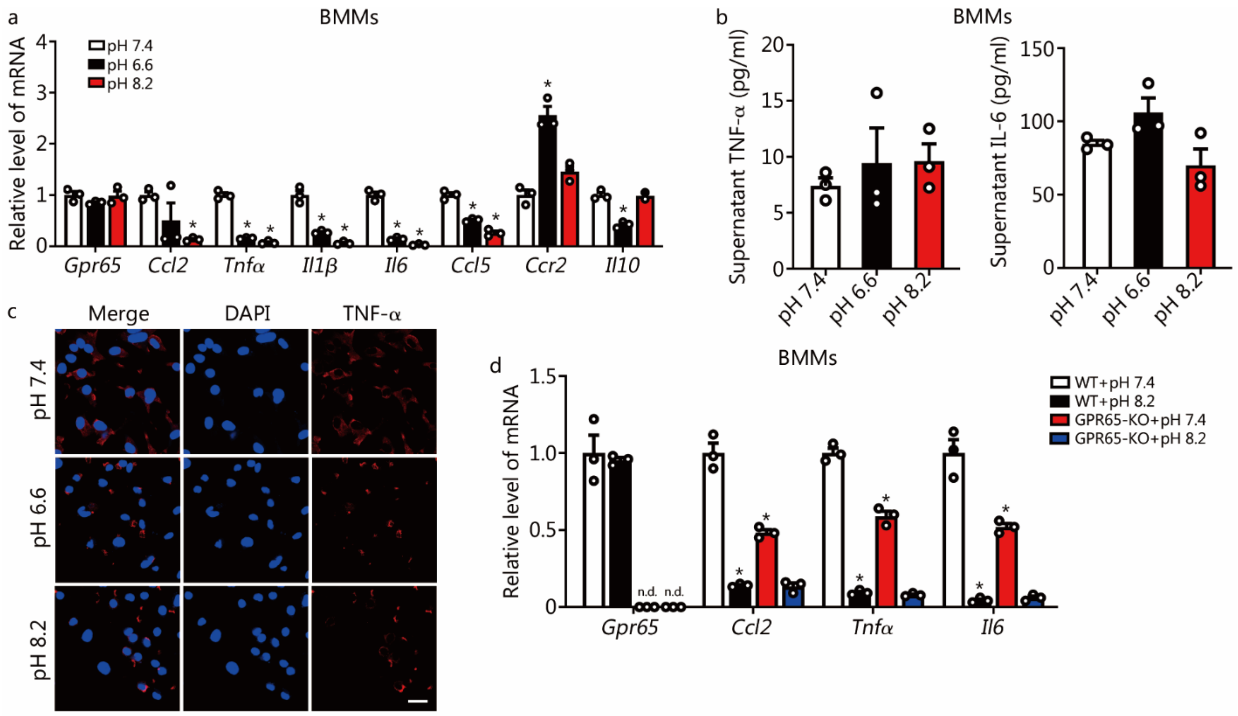

Fig. S10 Extracellular pH differentially regulates BMMs polarization partly via GPR65, related to **Fig. S9**. BMMs were incubated in pH 7.4, pH 6.6 or pH 8.2 for 24 h, qRT-PCR was used to assess the mRNA level of *Gpr65*, *Ccl2*, *Tnfα*, *Il1β*, *Il6*, *Ccl5*, *Ccr2* and *Il10* (n = 3, **a**); TNF-α and IL-6 level in the supernatant were detected by ELISA (n = 3, **b**); the expression and location of TNF-α was assessed by confocal microscopy (**c**). Scale bar = 20 μm. **d** BMMs isolated from WT and GPR65-KO mice were incubated in pH 7.4 and pH 8.2 for 24 h, qRT-PCR was used to assess the mRNA level of *Gpr65*, *Ccl2*, *Tnfα* and *Il6* (n = 3). $^{*}P < 0.05$ vs. pH 7.4 or WT + pH 7.4. BMM bone marrow-derived macrophage, ELISA enzyme-linked immunosorbent assay, KO knockout, qRT-PCR quantitative real-time reverse transcription-polymerase chain reaction, TNF-α tumor necrosis factor-α, IL-6 interleukin-6

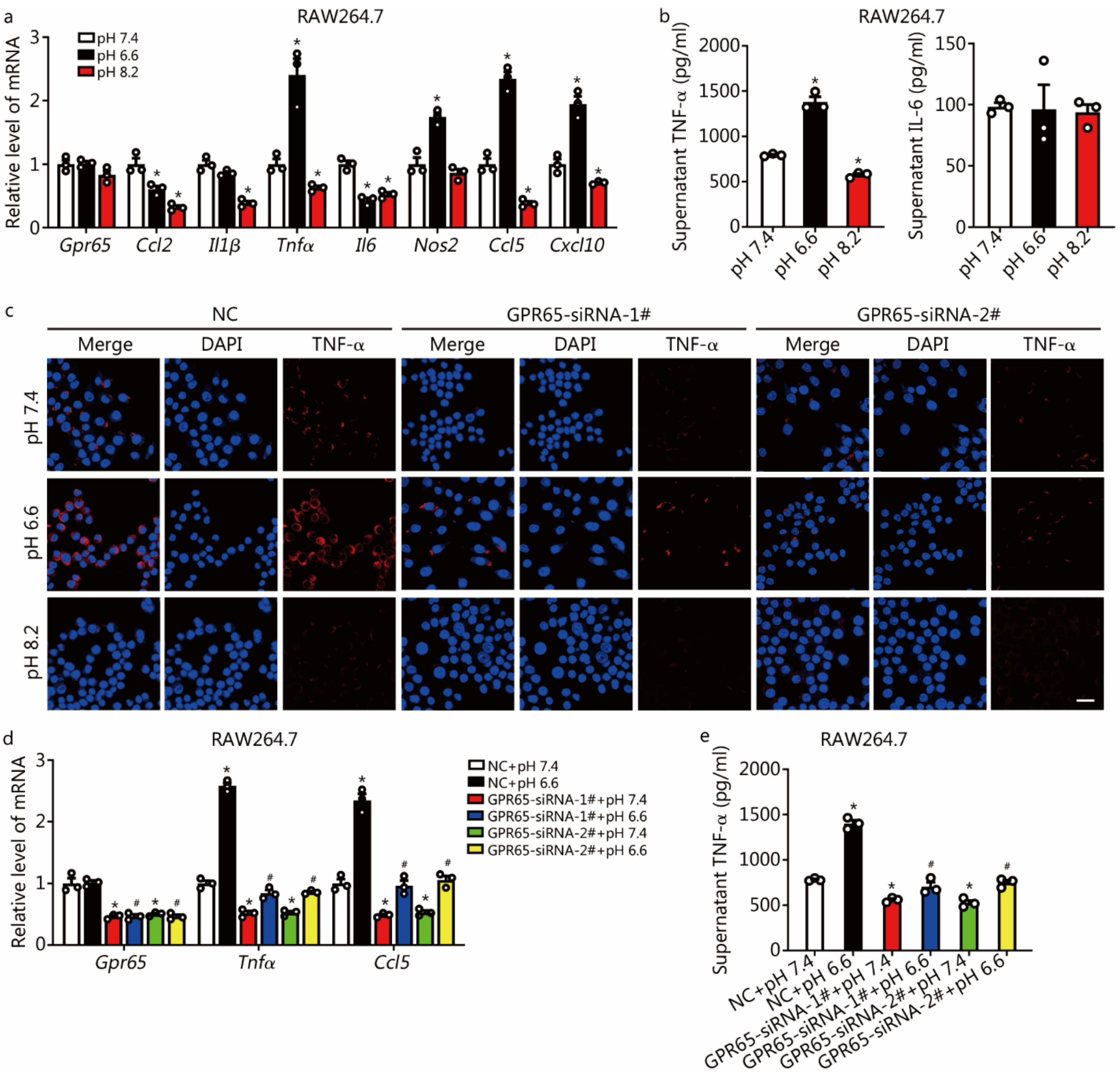

Fig. S11 Extracellular pH differentially regulates RAW264.7 cells polarization partly via GPR65, related to **Fig. S9**. RAW264.7 cells were incubated in pH 7.4, pH 6.6 or pH 8.2 for 24 h, qRT-PCR was used to assess the mRNA level of *Gpr65*, *Ccl2*, *Il1β*, *Tnfα*, *Il6*, *Nos2*, *Ccl5* and *Cxcl10* (n = 3, **a**); TNF-α and IL-6 level in the supernatant were detected by ELISA (n = 3, **b**). **c** RAW264.7 cells were transfected with NC, GPR65-siRNA-1# or GPR65-siRNA-2# for 24 h, followed by incubated in pH 7.4, pH 6.6 and pH 8.2 for 24 h, the expression and location of TNF-α was assessed by confocal microscopy. Scale bar = 20 µm. RAW264.7 cells were transfected with NC, GPR65-siRNA-1# or GPR65-siRNA-2# for 24 h, followed by incubated in pH 7.4 and pH 6.6 for 24 h, qRT-PCR was used to assess the mRNA level of *Gpr65*, *Tnfα* and *Ccl5* (n = 3, **d**); TNF-α level in the supernatant was detected by ELISA (n = 3, **e**). $^{*}P < 0.05$ vs. pH 7.4 or NC + pH 7.4; $^{\#}P < 0.05$ vs. NC + pH 6.6. ELISA enzyme-linked immunosorbent assay, qRT-PCR quantitative real-time reverse transcription-polymerase chain reaction, TNF-α tumor necrosis factor-α, IL-6 interleukin-6

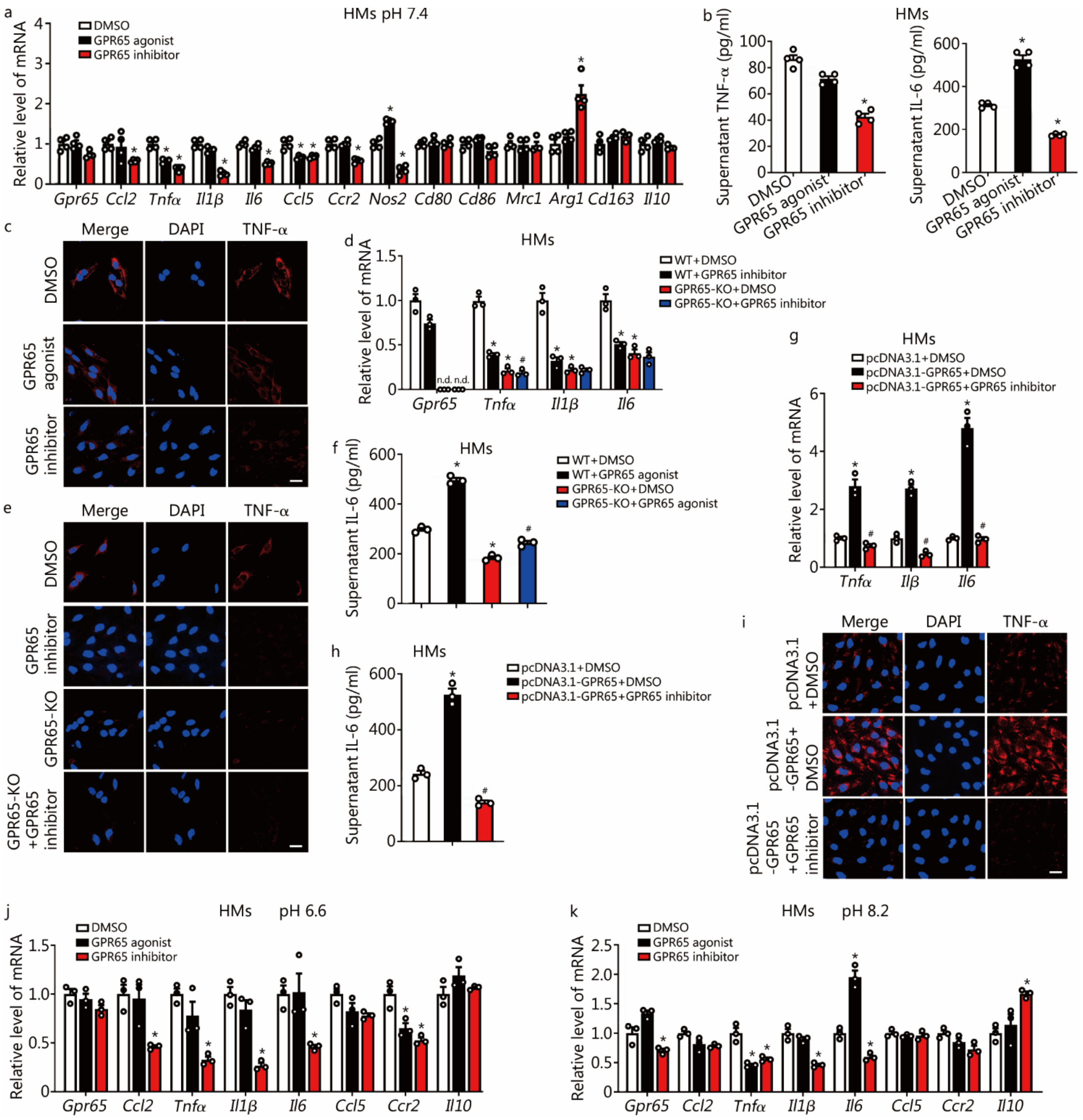

Fig. S12 Effect of GPR65 exogenous agonist and inhibitor on HMs polarization. HMs were treated with DMSO, 30 μmol/L GPR65 exogenous agonist BTB09089 or GPR65 exogenous inhibitor ZINC62678696 for 24 h, qRT-PCR was used to assess the mRNA level of *Gpr65*, *Ccl2*, *Tnfα*, *Il1β*, *Il6*, *Ccl5*, *Ccr2*, *Nos2*, *Cd80*, *Cd86*, *Mrc1*, *Arg1*, *Cd163* and *Il10* ($n = 4$, **a**); TNF-α and IL-6 level in the supernatant were detected by ELISA ($n = 4$, **b**); the expression and location of TNF-α was assessed by confocal microscopy (**c**). Scale bar = 20 μm. HMs isolated from WT and GPR65-KO mice were treated with DMSO or GPR65 agonist/inhibitor for 24 h, qRT-PCR was used to assess the mRNA level of *Gpr65*, *Tnfα*, *Il1β* and *Il6* ($n = 3$, **d**); the expression and location of TNF-α were assessed by confocal microscopy (**e**). Scale bar = 20 μm; IL-6 level in the supernatant was detected by ELISA ($n = 3$, **f**). HMs were transfected with pcDNA3.1 or pcDNA3.1-GPR65 for 48 h, followed by treated with DMSO or GPR65 inhibitor for 24 h, qRT-PCR was used to assess the mRNA level of *Tnfα*, *Il1β* and *Il6* ($n = 3$, **g**); IL-6 level in the supernatant was detected by ELISA ($n = 3$, **h**); the expression and location of TNF-α was assessed by confocal microscopy (**i**). Scale

bar = 20 μm. HMs were treated with DMSO, 30 μmol/L GPR65 agonist or GPR65 inhibitor at pH 6.6 (**j**) or pH 8.2 (**k**) for 24 h, qRT-PCR was used to assess the mRNA level of *Gpr65*, *Ccl2*, *Tnfα*, *Il1β*, *Il6*, *Ccl5*, *Ccr2* and *Il10* (n = 3). $^{*}P < 0.05$ vs. DMSO, WT + DMSO or pcDNA3.1 + DMSO; $^{\#}P < 0.05$ vs. WT + GPR65 agonist/inhibitor or pcDNA3.1-GPR65 + DMSO. HM hepatic macrophage, ELISA enzyme-linked immunosorbent assay, KO knockout, qRT-PCR quantitative real-time reverse transcription-polymerase chain reaction, TNF-α tumor necrosis factor-α, IL-6 interleukin-6

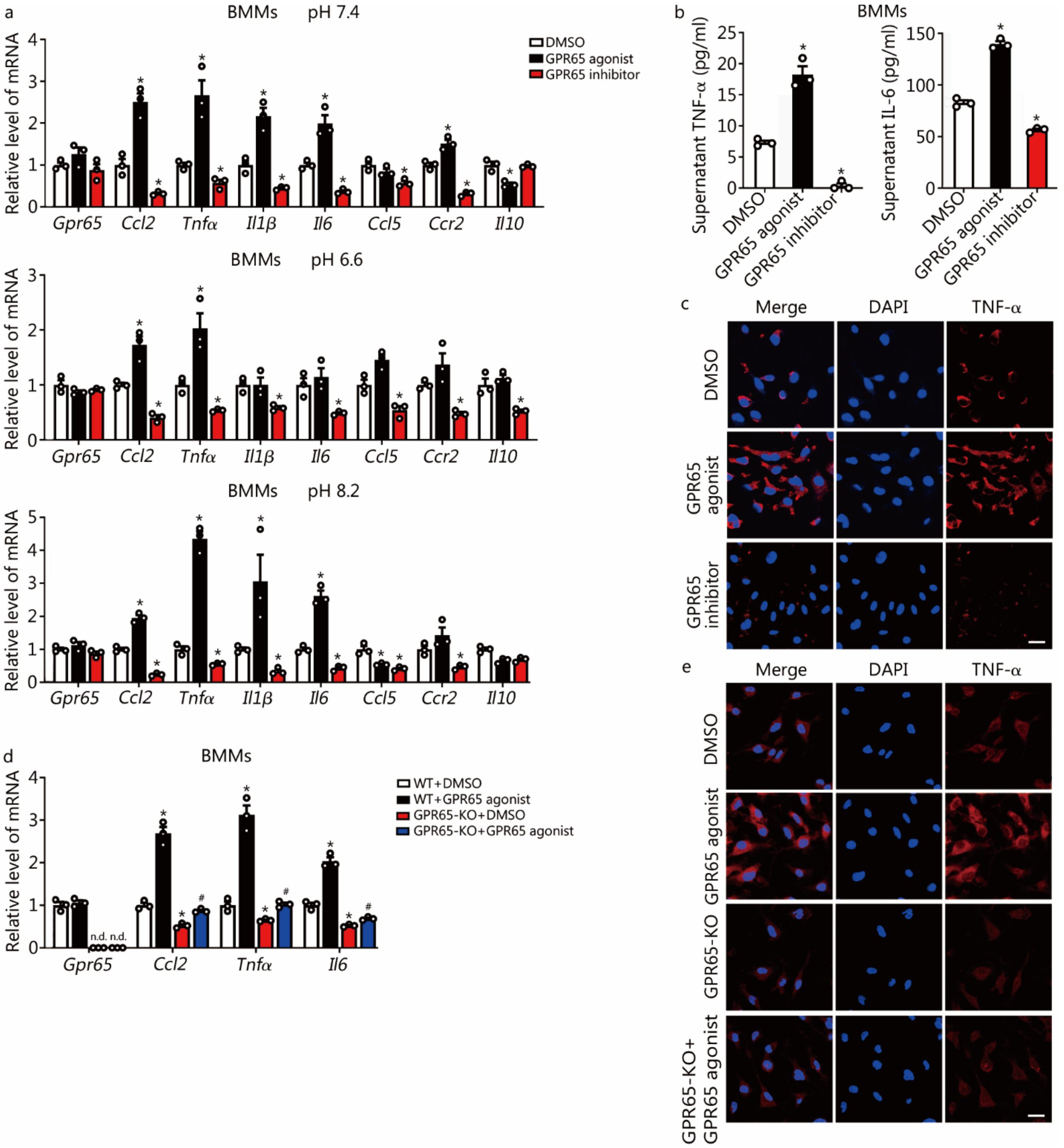

Fig. S13 Effect of GPR65 exogenous agonist and inhibitor on BMMs polarization, related to **Fig. S12**. **a** BMMs were treated with DMSO, 30 μmol/L GPR65 agonist or GPR65 inhibitor at pH 7.4, pH 6.6 or pH 8.2 for 24 h, qRT-PCR was used to assess the mRNA level of *Gpr65*, *Ccl2*, *Tnfα*, *Il1β*, *Il6*, *Ccl5*, *Ccr2* and *Il10* (n = 3). BMMs were treated with DMSO, 30 μmol/L GPR65 agonist or GPR65 inhibitor at pH 7.4 for 24 h, TNF-α and IL-6 level in the supernatant were detected by ELISA (n = 3, **b**); the expression and location of TNF-α was assessed by confocal microscopy (**c**). Scale bar = 20 μm. BMMs isolated from WT and GPR65-KO mice were treated with DMSO or GPR65 agonist for 24 h, qRT-PCR was used to assess the mRNA level of *Gpr65*, *Ccl2*, *Tnfα* and *Il6* (n = 3, **d**); the expression and location of TNF-α was assessed by confocal microscopy (**e**). Scale bar = 20 μm. $^{*}P < 0.05$ vs. DMSO or WT + DMSO; $^{\#}P < 0.05$ vs. WT + GPR65 agonist. BMM bone marrow-derived macrophage, ELISA enzyme-linked immunosorbent assay, KO knockout, qRT-PCR quantitative real-time reverse transcription-polymerase chain reaction, TNF-α tumor necrosis factor-α, IL-6 interleukin-6

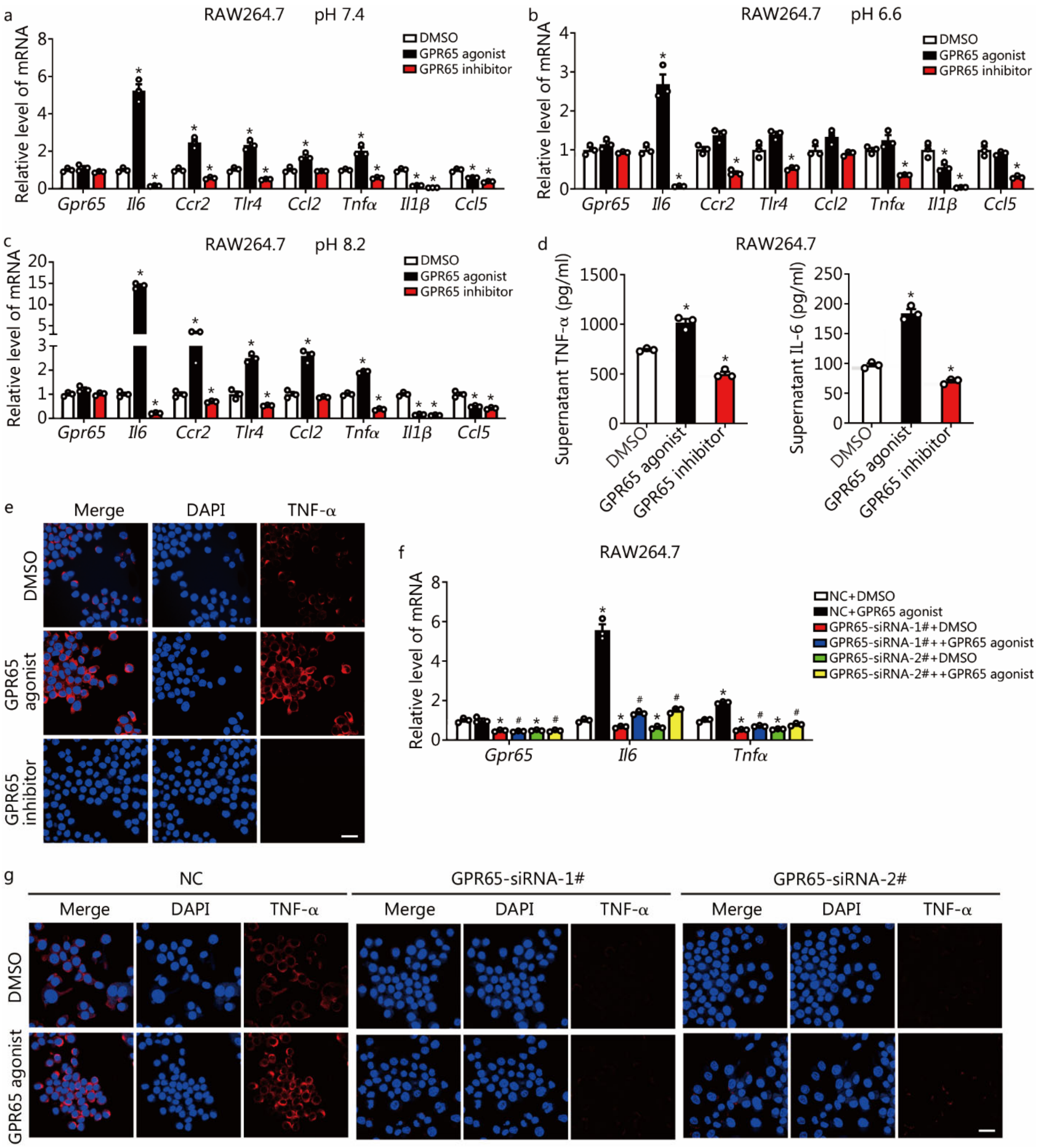

Fig. S14 Effect of GPR65 exogenous agonist and inhibitor on RAW264.7 cells polarization, related to **Fig. S12**. RAW264.7 cells were treated with DMSO, 30 μmol/L GPR65 agonist or GPR65 inhibitor at pH 7.4 (**a**), pH 6.6 (**b**) or pH 8.2 (**c**) for 24 h, qRT-PCR was used to assess the mRNA level of *Gpr65*, *Il6*, *Ccr2*, *Tlr4*, *Ccl2*, *Tnfα*, *Il1β* and *Ccl5* ($n = 3$). RAW264.7 cells were treated with DMSO, 30 μmol/L GPR65 agonist or GPR65 inhibitor at pH 7.4 for 24 h, TNF-α and IL-6 level in the supernatant were detected by ELISA ($n = 3$, **d**); the expression and location of TNF-α were assessed by confocal microscopy (**e**). Scale bar = 20 μm. RAW264.7 cells were transfected with NC, GPR65-siRNA-1# or GPR65-siRNA-2# for 24 h, followed by treated with DMSO or GPR65 agonist for 24 h, qRT-PCR was used to assess the mRNA level of *Gpr65*, *Il6* and *Tnfα* ($n = 3$, **f**); the expression and location of TNF-α was assessed by confocal microscopy (**g**). Scale bar = 20 μm. $^{*}P < 0.05$ vs. DMSO or NC + DMSO; $^{\#}P < 0.05$ vs. NC + GPR65 agonist. ELISA enzyme-linked immunosorbent assay, qRT-PCR quantitative real-time reverse transcription-polymerase chain reaction, TNF-α tumor necrosis factor-α, IL-6 interleukin-6

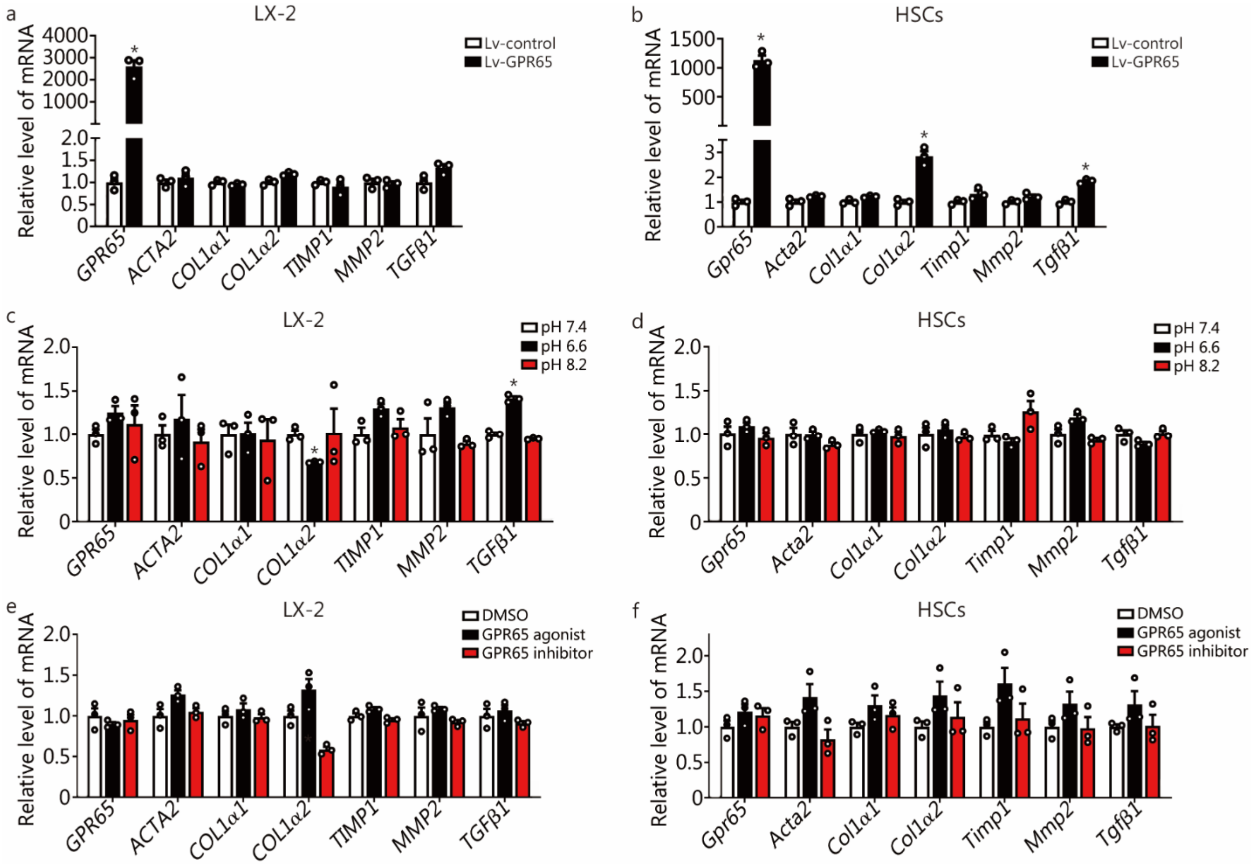

Fig. S15 GPR65 is not involved in regulation of HSCs activation directly, related to **Fig. 5**. LX-2 cells (**a**) and mouse primary HSCs (**b**) were infected with lentivirus-mediated control or GPR65 for 72 h, qRT-PCR was used to assess the expression of *GPR65*, *ACTA2*, *COL1α1*, *COL1α2*, *TIMP1*, *MMP2* and *TGFβ1* ($n = 3$). LX-2 cells (**c**) and mouse primary HSCs (**d**) were incubated in pH 7.4, pH 6.6 or pH 8.2 for 24 h, qRT-PCR was used to assess the expression of *GPR65*, *ACTA2*, *COL1α1*, *COL1α2*, *TIMP1*, *MMP2* and *TGFβ1* ($n = 3$). LX-2 cells (**e**) and mouse primary HSCs (**f**) were treated with DMSO, 30 μmol/L GPR65 agonist or GPR65 inhibitor for 24 h, qRT-PCR was used to assess the expression of *GPR65*, *ACTA2*, *COL1α1*, *COL1α2*, *TIMP1*, *MMP2* and *TGFβ1* ($n = 3$). $^{*}P < 0.05$ vs. Lv-control, pH 7.4 or DMSO. HSC hepatic stellate cell, qRT-PCR quantitative real-time reverse transcription-polymerase chain reaction

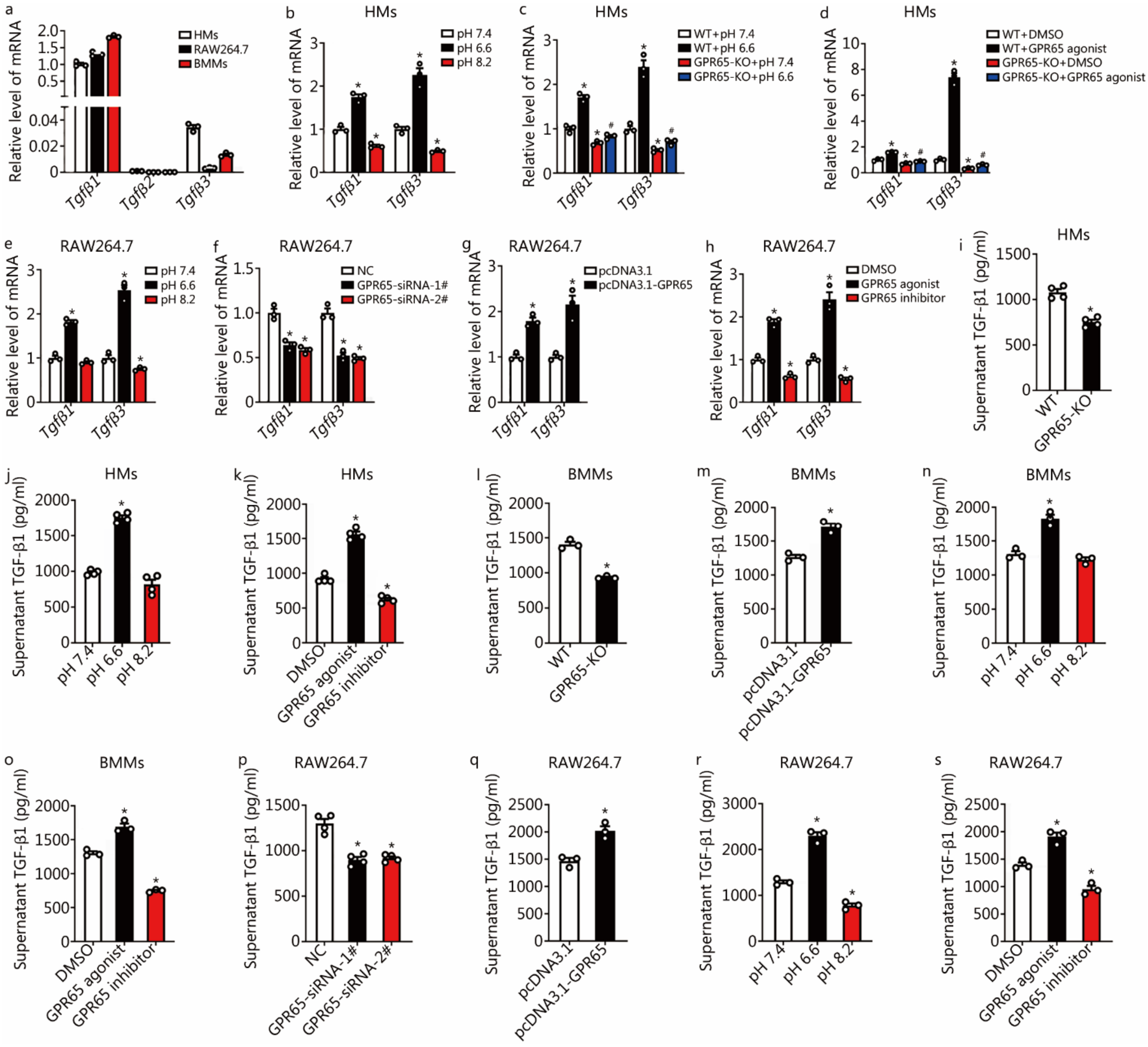

Fig. S16 GPR65 promotes the expression and release of TGF-β, related to **Fig. 5**. **a** qRT-PCR was used to assess the expression of *Tgfβ1*, *Tgfβ2* and *Tgfβ3* in HMs, RAW264.7 cells and BMMs (n = 3). **b-h** qRT-PCR was used to assess the expression of *Tgfβ1* and *Tgfβ3* in GPR65-KO, GPR65-silenced, GPR65-overexpressed, various pH-treated or GPR65 agonist/inhibitor-treated HMs or RAW264.7 cells (n = 3). **i-s** TGF-β1 level in the supernatant GPR65-KO, GPR65-silenced, GPR65-overexpressed, various pH-treated or GPR65 agonist/inhibitor-treated HMs, BMMs and RAW264.7 cells was detected by ELISA (n = 3 or 4). $^*P < 0.05$ vs. pH 7.4, WT + pH 7.4/DMSO, NC, pcDNA3.1, DMSO or WT; $^\#P < 0.05$ vs. WT + pH 6.6/GPR65 agonist. BMM bone marrow-derived macrophage, HM hepatic macrophage, ELISA enzyme-linked immunosorbent assay, KO knockout, qRT-PCR quantitative real-time reverse transcription-polymerase chain reaction, TGF-β1 transforming growth factor-β1

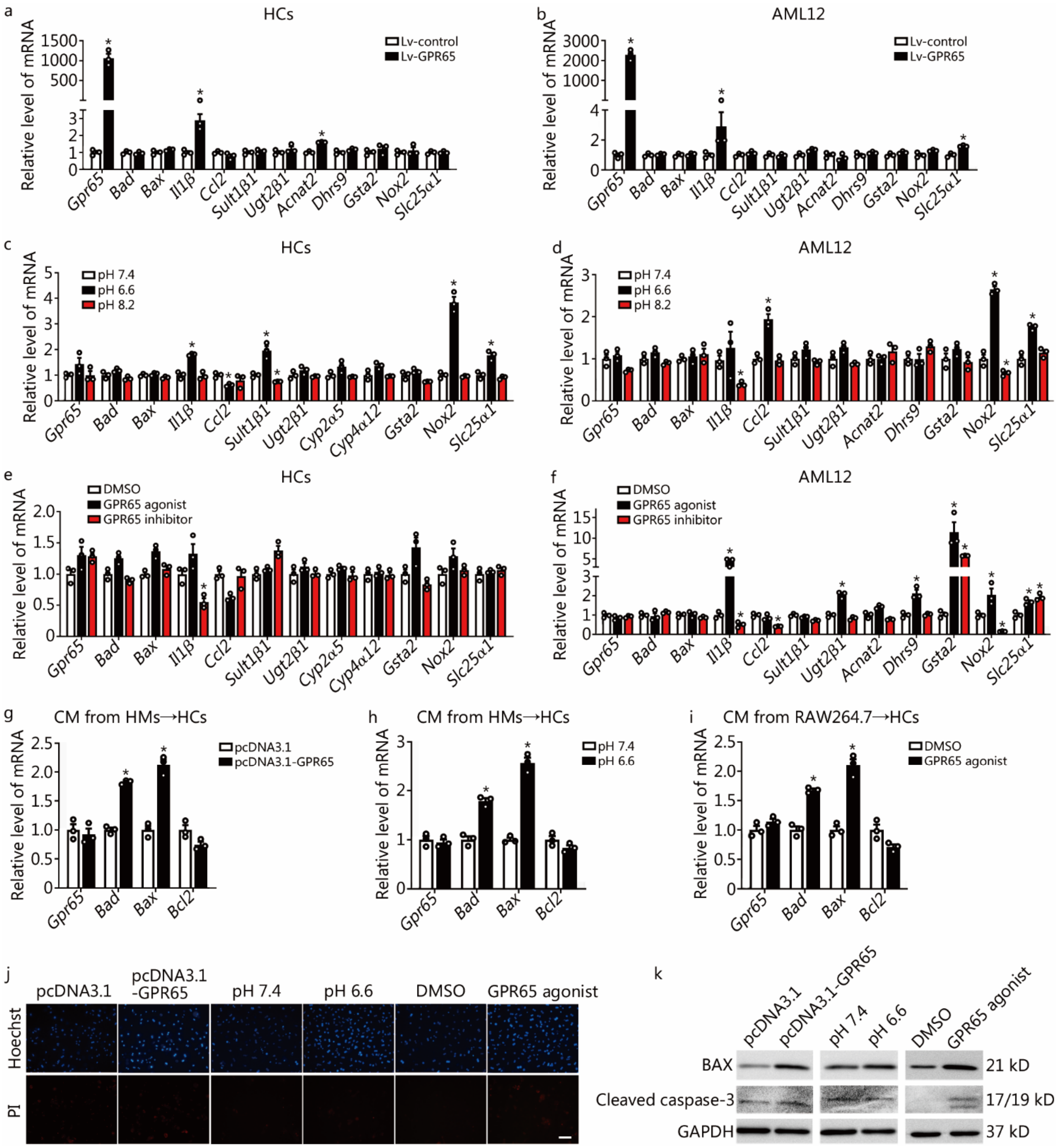

Fig. S17 GPR65 aggravates HCs damage through the signals derived from macrophage. Mouse primary HCs (**a**) and AML12 cells (**b**) were infected with lentivirus-mediated control or GPR65 for 72 h, qRT-PCR was used to assess the expression of *Gpr65,* apoptosis-related genes (*Bax, Bad*), inflammation-related genes (*Il1β, Ccl2*) and metabolism-related genes (*Sult1β1*, *Ugt2β1*, *Cyp2α5*, *Cyp4α12*, *Gsta2*, *Nox2*, *Slc25α1*, *Acnat2*, *Dhrs9*, $n = 3$). Mouse primary HCs (**c**) and AML12 cells (**d**) were incubated in pH 7.4, pH 6.6 or pH 8.2 for 24 h, qRT-PCR was used to assess the expression of *Gpr65,* apoptosis-related genes, inflammation-related genes and metabolism-related genes ($n = 3$). Mouse primary HCs (**e**) and AML12 cells (**f**) were treated with DMSO, 30 μmol/L GPR65 agonist or GPR65 inhibitor for 24 h, qRT-PCR was used to assess the expression of *Gpr65,* apoptosis-related genes, inflammation-related genes and metabolism-related genes ($n = 3$). The CM from control, GPR65-overexpressed (**g**), pH 6.6-treated (**h**) or GPR65

agonist-treated (**i**) macrophage cells were used to treat primary HCs for 24 h. qRT-PCR was used to assess the mRNA level of *Gpr65, Bad, Bax* and *Bcl2* ($n = 3$); the apoptosis of HCs was determined by Hoechst/PI double staining (**j**). Scale bar = 100 μm; Western blotting was used to determine the protein level of BAX and cleaved caspase-3 (**k**). $^{*}P < 0.05$ vs. Lv-control, pH 7.4, DMSO or pcDNA3.1. CM conditioned medium, HC hepatocyte, HM hepatic macrophage, KO knockout, qRT-PCR quantitative real-time reverse transcription-polymerase chain reaction

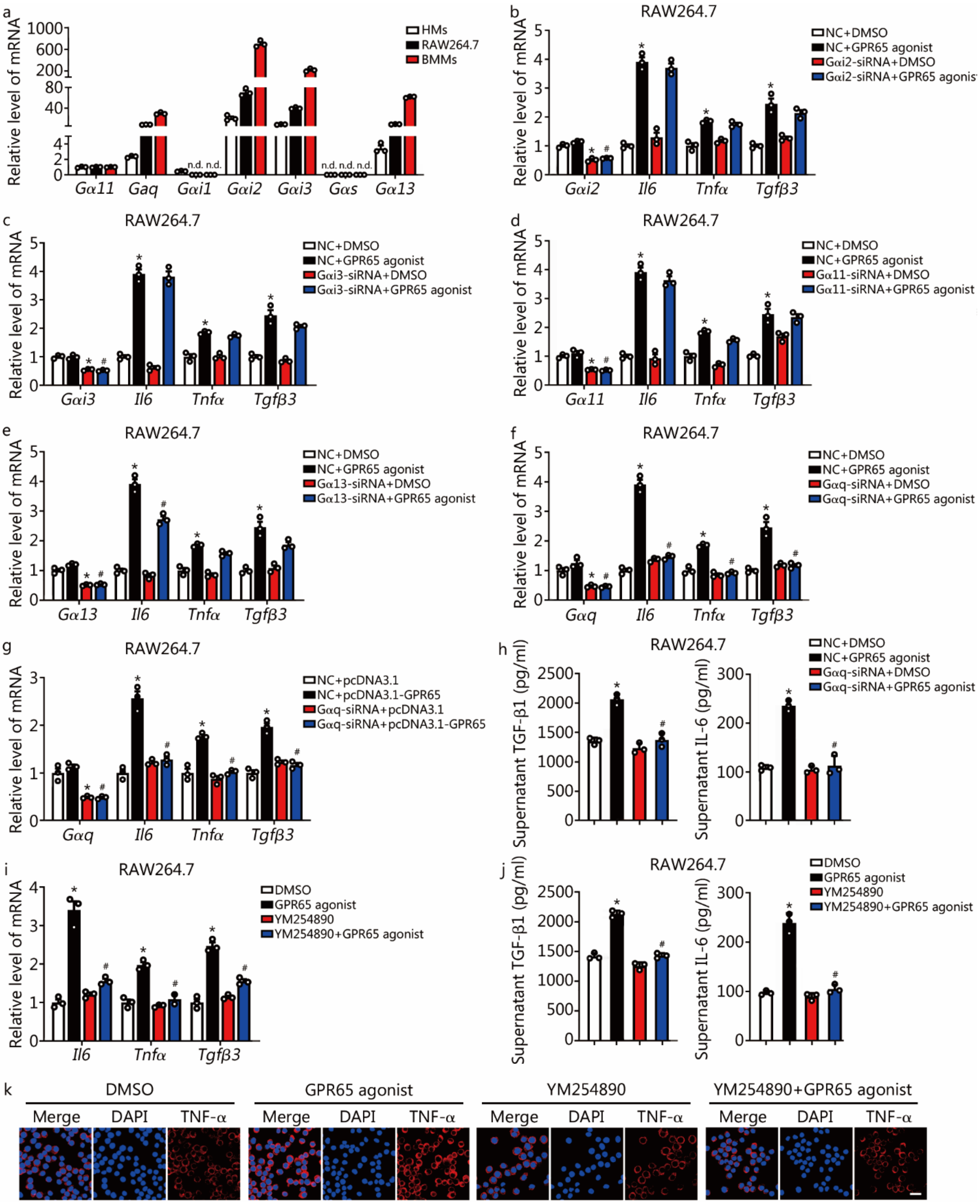

Fig. S18 Targeting GPR65 alleviates inflammation via Gαq. **a** qRT-PCR analysis of the expression of *Gαs, Gai1, Gαi2, Gαi3, Gαq, Gα11* and *Gα13* in HMs, RAW264.7 cells and BMMs (n = 3). RAW264.7 cells were transfected with NC, Gαi2-siRNA (**b**), Gαi3-siRNA (**c**), Gα11-siRNA (**d**), Gα13-siRNA (**e**) or Gαq-siRNA (**f**) for 24 h, followed by treated with DMSO or GPR65 agonist for 24 h, qRT-PCR was used to assess the mRNA level of *Gα*, *Il6*, *Tnfα* and *Tgfβ3* (n = 3). **g** RAW264.7 cells were transfected with NC or Gαq-siRNA for 12 h, followed by transfected with pcDNA3.1 or pcDNA3.1-GPR65 for 36 h, qRT-PCR was used to assess the mRNA level of *Gαq*, *Il6*, *Tnfα* and *Tgfβ3* (n = 3). **h** RAW264.7 cells were transfected with NC or Gαq-siRNA for 24 h, followed by treated with DMSO or GPR65 agonist for 24 h, IL-6 and TGF-β1 level in the supernatant were detected by ELISA (n = 3). Gαq inhibitor

YM254890 (1 μmol/L) was used to treat GPR65 agonist-treated RAW264.7 cells for 20 h, qRT-PCR was used to assess the mRNA level of *Il6*, *Tnfα* and *Tgfβ3* (n = 3, **i**); TGF-β1 and IL-6 level in the supernatant were detected by ELISA (n = 3, **j**); the expression and location of TNF-α was assessed by confocal microscopy (**k**). Scale bar = 20 μm. $^{*}P < 0.05$ vs. DMSO, NC + DMSO or NC + pcDNA3.1; $^{\#}P < 0.05$ vs. NC + GPR65 agonist, NC + pcDNA3.1-GPR65 or GPR65 agonist. ELISA enzyme-linked immunosorbent assay, qRT-PCR quantitative real-time reverse transcription-polymerase chain reaction, TGF-β1 transforming growth factor-β1, IL-6 interleukin-6, TNF-α tumor necrosis factor-α

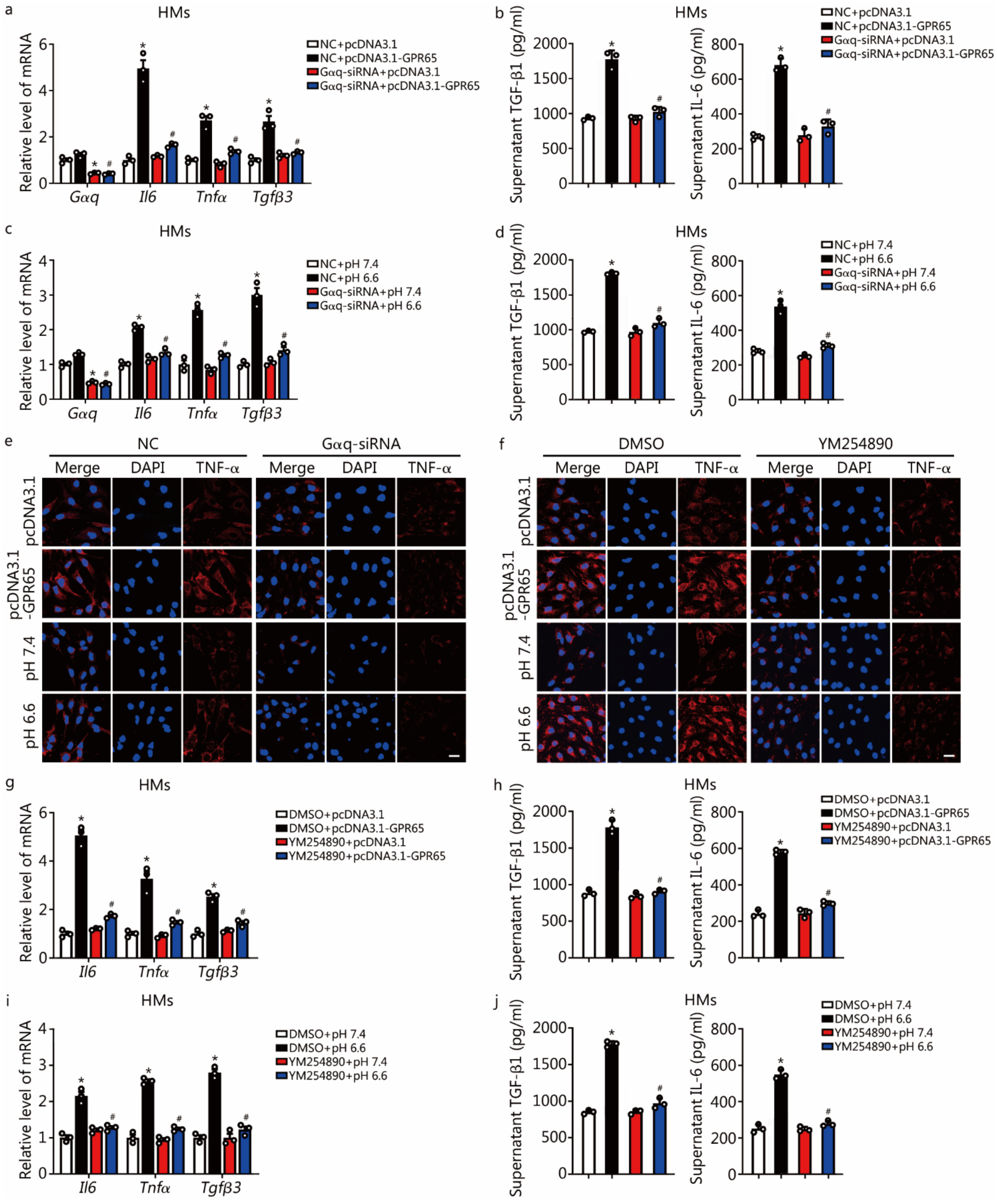

Fig. S19 Targeting GPR65 alleviates hepatic macrophage inflammation via Gαq, related to **Fig. S18**. HMs were transfected with NC or Gαq-siRNA for 12 h, followed by transfected with pcDNA3.1 or pcDNA3.1-GPR65 for 36 h, qRT-PCR was used to assess the mRNA level of *Gαq*, *Il6*, *Tnfα* and *Tgfβ3* (*n* = 3, **a**); TGF-β1 and IL-6 level in the supernatant were detected by ELISA (*n* = 3, **b**). HMs were transfected with NC or Gαq-siRNA for 24 h, followed by incubated in pH 7.4 or pH 6.6 for 24 h, qRT-PCR was used to assess the mRNA level of *Gαq*, *Il6*, *Tnfα* and *Tgfβ3* (*n* = 3, **c**); TGF-β1 and IL-6 level in the supernatant were detected by ELISA (*n* = 3, **d**). **e** HMs were transfected with NC or Gαq-siRNA for 12 or 24 h, followed by transfected with pcDNA3.1 or pcDNA3.1-GPR65 for 36 h, or

incubated in pH 7.4 or pH 6.6 for 24 h, the expression and location of TNF-α was assessed by confocal microscopy. Scale bar = 20 μm. **f-j** HMs were transfected with pcDNA3.1 or pcDNA3.1-GPR65 for 24 h, or incubated in pH 7.4 or pH 6.6 for 4 h, followed by treated with 1 μmol/L Gαq inhibitor YM254890 for 20 h, the expression and location of TNF-α was assessed by confocal microscopy (**f**). Scale bar = 20 μm. qRT-PCR was used to assess the mRNA level of *Il6*, *Tnfα* and *Tgfβ3* (n = 3, **g, i**); TGF-β1 and IL-6 level in the supernatant were detected by ELISA (n = 3, **h, j**). $^{*}P < 0.05$ vs. NC + pcDNA3.1/pH7.4 or DMSO + pcDNA3.1/ pH7.4; $^{\#}P < 0.05$ vs. NC + pcDNA3.1-GPR65/pH6.6 or DMSO + pcDNA3.1-GPR65/pH6.6. HM hepatic macrophage, ELISA enzyme-linked immunosorbent assay, qRT-PCR quantitative real-time reverse transcription-polymerase chain reaction, TGF-β1 transforming growth factor-β1, IL-6 interleukin-6, TNF-α tumor necrosis factor-α

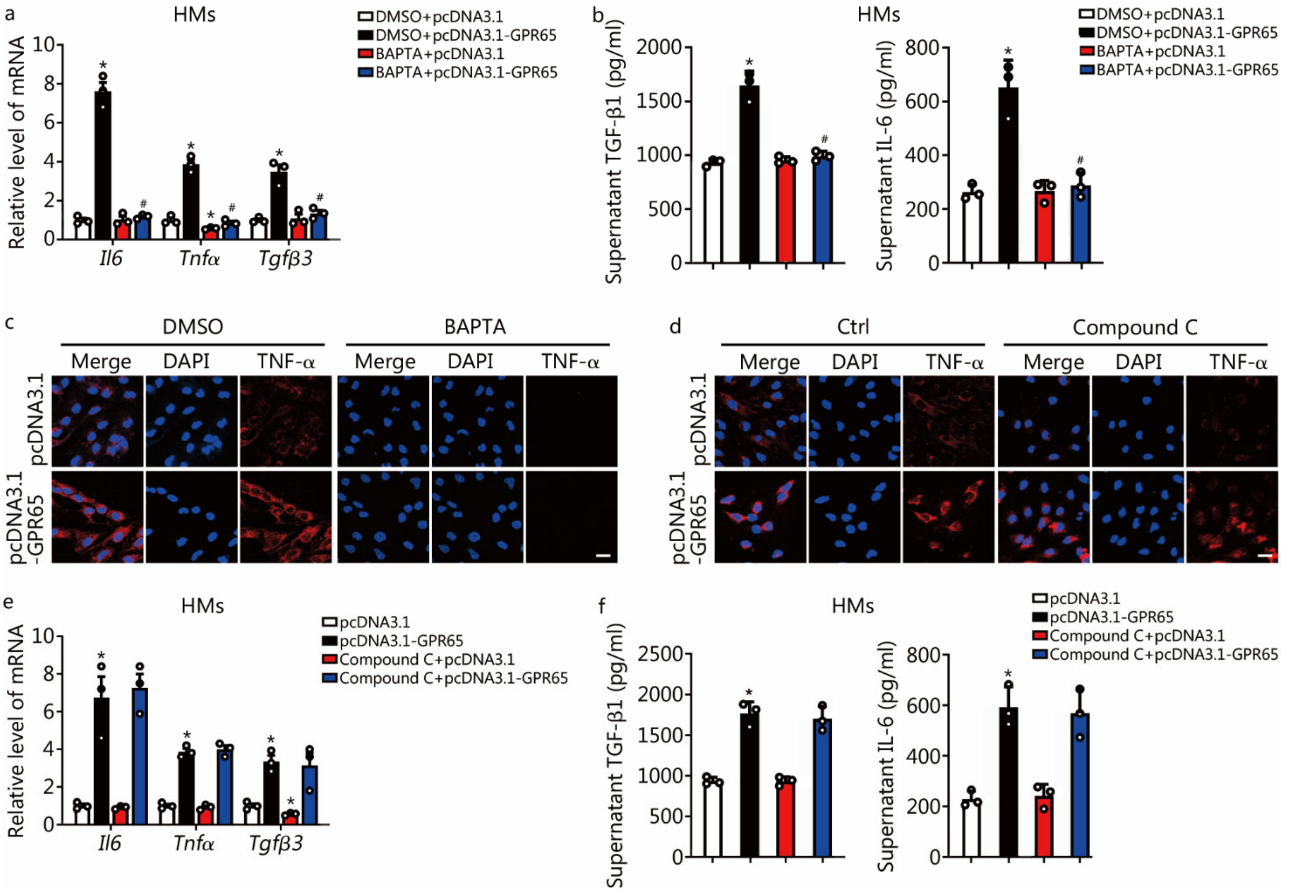

Fig. S20 Targeting GPR65 alleviates hepatic macrophage inflammation via Ca^{2+} rather than AMPK, related to **Fig. S18**. HMs were transfected with pcDNA3.1 or pcDNA3.1-GPR65 for 24 h, followed by treated with 10 μmol/L BAPTA (Ca^{2+} chelator) for 24 h, qRT-PCR was used to assess the mRNA level of *Il6*, *Tnfα* and *Tgfβ3* (n = 3, **a**); TGF-β1 and IL-6 level in the supernatant were detected by ELISA (n = 3, **b**); the expression and location of TNF-α was assessed by confocal microscopy (**c**). Scale bar = 20 μm. HMs were transfected with pcDNA3.1 or pcDNA3.1-GPR65 for 24 h, followed by treated with 10 μmol/L Compound C (AMPK inhibitor) for 12 h, the expression and location of TNF-α was assessed by confocal microscopy (**d**). Scale bar = 20 μm; qRT-PCR was used to assess the mRNA level of *Il6*, *Tnfα* and *Tgfβ3* (n = 3, **e**); TGF-β1 and IL-6 level in the supernatant were detected by ELISA (n = 3, **f**). $^{*}P < 0.05$ vs. pcDNA3.1 or DMSO + pcDNA3.1; $^{\#}P < 0.05$ vs. DMSO + pcDNA3.1-GPR65. HM hepatic macrophage, ELISA enzyme-linked immunosorbent assay, qRT-PCR quantitative real-time reverse transcription-polymerase chain reaction, TGF-β1 transforming growth factor-β1, IL-6 interleukin-6, TNF-α tumor necrosis factor-α

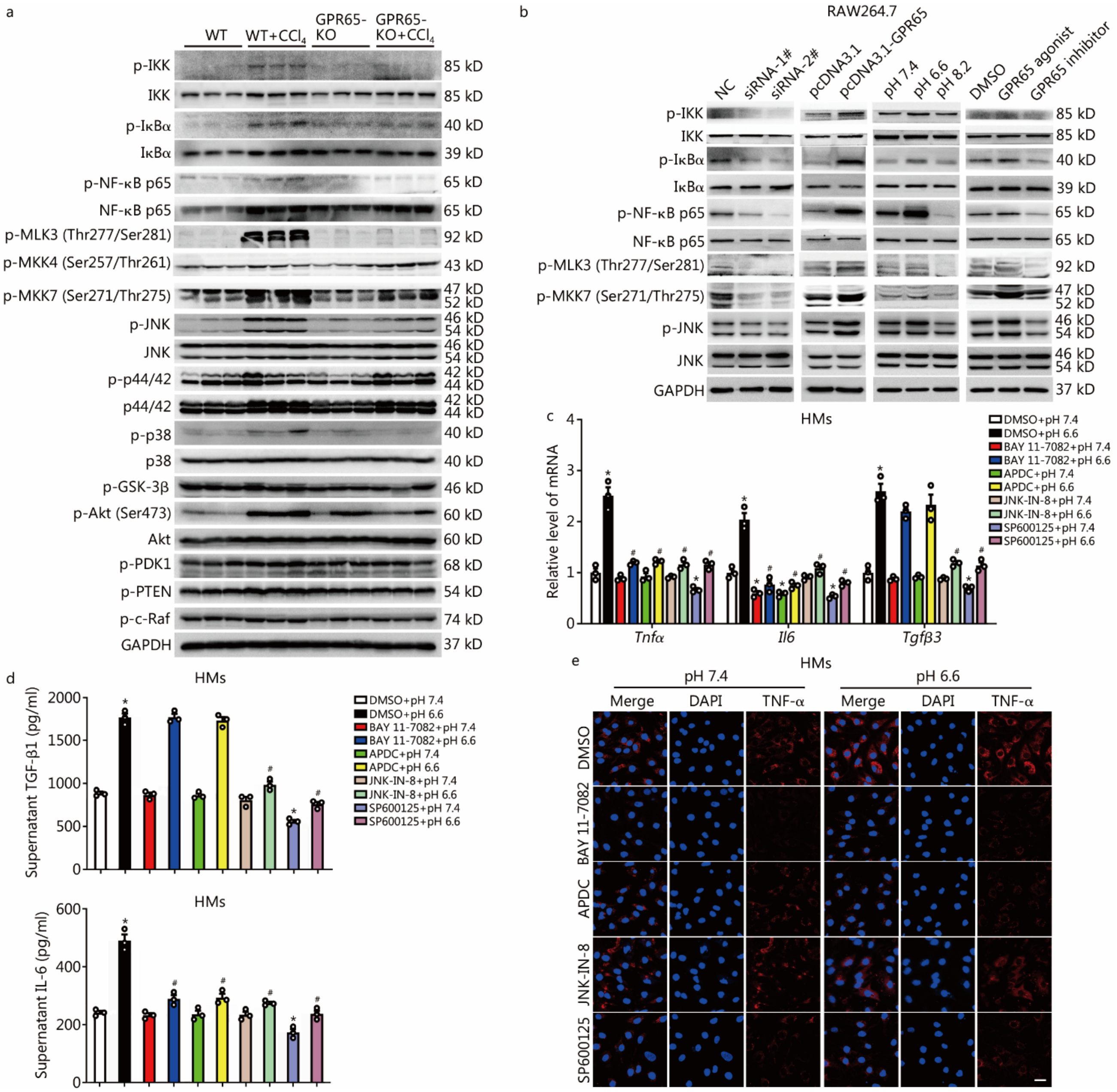

Fig. S21 Targeting GPR65 alleviates hepatic macrophage inflammation by suppressing the JNK/NF-κB pathways, related to **Fig. 6**. **a** Western blotting was used to determine the level of p-IKK, p-IκBα, p-NF-κB p65, IKK, IκBα, NF-κB p65, p-MLK3, p-MKK4, p-MKK7, p-JNK, JNK, p-p44/42, p44/42, p-p38, p38, p-GSK-3β, p-Akt (Ser473), Akt, p-PDK1, p-PTEN and p-c-Raf in liver tissues from WT, WT + CCl_4, GPR65-KO and GPR65-KO + CCl_4 mice. **b** Western blotting was used to determine the level of p-IKK, IKK, p-IκBα, IκBα, p-NF-κB p65, NF-κB p65, p-MLK3, p-MKK7, p-JNK and JNK in GPR65-silenced, GPR65-overexpressed, various pH-treated and GPR65 agonist/inhibitor-treated RAW264.7 cells. The specific inhibitors of JNK, SP600125 (10 μmol/L) and JNK-IN-8 (5 μmol/L), as well as the specific inhibitors of NF-κB, BAY 11-7082 (5 μmol/L) and APDC (20 μmol/L), were used to treat pH 6.6-treated HMs, qRT-PCR was used to assess the mRNA level of *Il6*, *Tnfα* and *Tgfβ3* (n = 3, **c**); TGF-β1 and IL-6 level in the supernatant were detected by ELISA (n = 3, **d**); the expression and location of TNF-α was assessed by confocal microscopy (**e**). Scale bar = 20 μm. $^*P < 0.05$ vs. DMSO + pH 7.4; $^\#P < 0.05$ vs. DMSO + pH 6.6. CCl_4 carbon tetrachloride, HM hepatic macrophage, ELISA enzyme-linked immunosorbent assay, KO knockout,

qRT-PCR quantitative real-time reverse transcription-polymerase chain reaction, IKK inhibitor of nuclear factor κB kinase, IκBα inhibitor of κB α, NF-κB p65 nuclear factor κB p65 subunit, MLK3 mixed lineage kinase 3, MKK4 mitogen-activated protein kinase kinase 4, MKK7 mitogen-activated protein kinase kinase 7, JNK c-Jun N-terminal kinase, GSK-3β glycogen synthase kinase 3β, Akt protein kinase B, PDK1 pyruvate dehydrogenase kinase-1, PTEN phosphatase and tensin homolog, c-Raf c-rapidly accelerated fibrosarcoma, TGF-β1 transforming growth factor-β1, IL-6 interleukin-6, TNF-α tumor necrosis factor-α

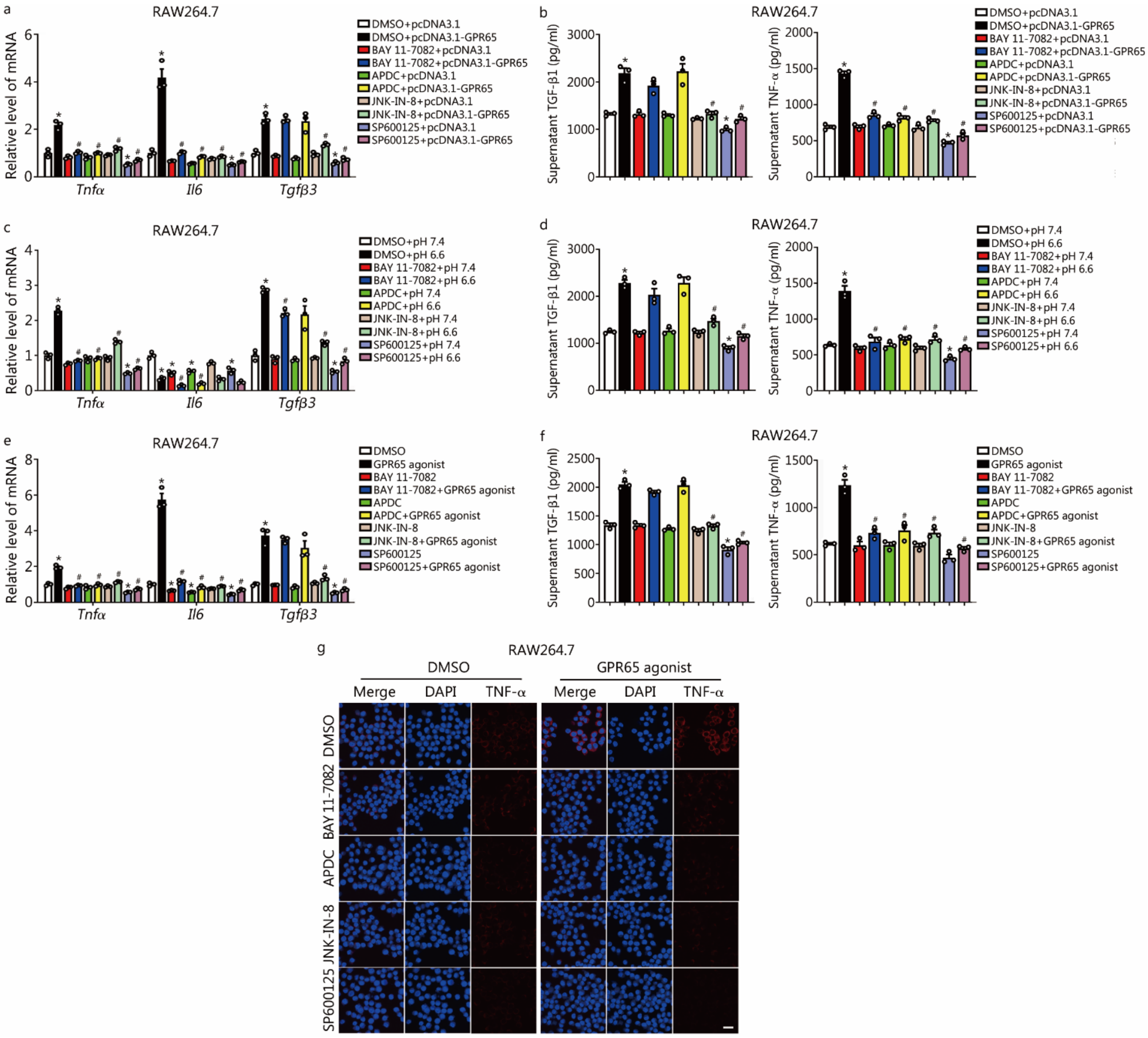

Fig. S22 Targeting GPR65 alleviates inflammation by suppressing the JNK/NF-κB pathways, related to **Fig. 6**. The specific inhibitors of JNK, SP600125 (10 μmol/L) and JNK-IN-8 (5 μmol/L), as well as the specific inhibitors of NF-κB, BAY 11-7082 (5 μmol/L) and APDC (20 μmol/L), were used to treat GPR65-overexpressed- (**a, b**), pH 6.6-treated (**c, d**) or GPR65 agonist-treated (**e, f**) RAW264.7 cells, qRT-PCR was used to assess the mRNA level of *Tnfα*, *Il6* and *Tgfβ3* (n = 3, **a, c, e**); TGF-β1 and TNF-α level in the supernatant were detected by ELISA (n = 3, **b, d, f**); the expression and location of TNF-α was assessed by confocal microscopy (**g**). Scale bar = 20 μm. $^{*}P < 0.05$ vs. DMSO + pcDNA3.1/pH 7.4 or DMSO; $^{\#}P < 0.05$ vs. DMSO + pcDNA3.1-GPR65/pH 6.6 or GPR65 agonist. ELISA enzyme-linked immunosorbent assay, qRT-PCR quantitative real-time reverse transcription-polymerase chain reaction, TGF-β1 transforming growth factor-β1, TNF-α tumor necrosis factor-α

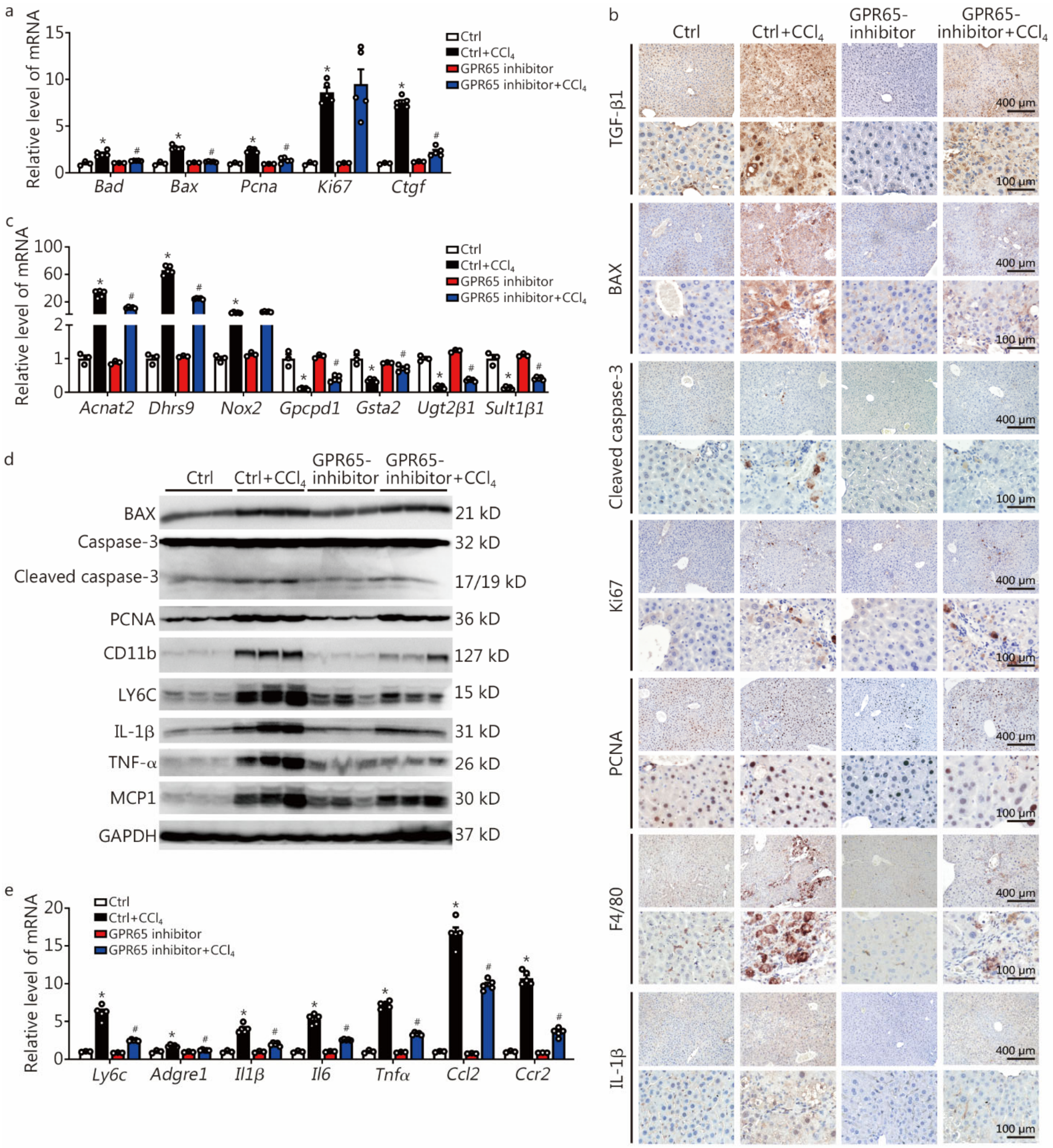

Fig. S23 Pharmacological GPR65 inhibition alleviates CCl_4-induced hepatic injury and inflammation, related to **Fig. 7**. **a** BALB/c mice were divided into 4 groups randomly: Ctrl, Ctrl + CCl_4, GPR65 inhibitor and GPR65 inhibitor + CCl_4. The GPR65 inhibitor (10 mg/kg) was administered 4 weeks after the first CCl_4 injection every two days. Mice were administered CCl_4 for 8 weeks. qRT-PCR was used to assess the mRNA level of *Bad*, *Bax*, *Pcna*, *Ki67* and *Ctgf* (n = 3, 5, 3, 5). **b** IHC for TGF-β1, BAX, cleaved caspase-3, Ki67, PCNA, F4/80 and IL-1β. Scale bar = 100 μm for 40× and 400 μm for 10×. **c** qRT-PCR was used to assess the mRNA level of *Acnat2*, *Dhrs9*, *Nox2*, *Gpcpd1*, *Gsta2*, *Ugt2β1* and *Sult1β1* (n = 3, 5, 3, 5). **d** Western blotting was used to determine the protein level of BAX, cleaved caspase-3, PCNA, CD11b, LY6C, IL-1β, TNF-α and MCP1. **e** qRT-PCR was used to assess the mRNA level of *Ly6c*, *Adgre1*, *Il1β*, *Il6*, *Tnfα*, *Ccl2* and *Ccr2* (n = 3, 5, 3, 5). $^*P < 0.05$ vs. Ctrl; $^\#P < 0.05$ vs. Ctrl + CCl_4. CCl_4 carbon tetrachloride, IHC immunohistochemistry, qRT-PCR quantitative real-time reverse transcription-polymerase chain reaction, TGF-β1 transforming growth factor-β1, BAX bcl2-associated X protein, PCNA proliferating cell nuclear

antigen, IL-1β interleukin-1β, LY6C lymphocyte antigen 6 complex, TNF-α tumor necrosis factor-α, MCP1 monocyte chemoattractant protein 1

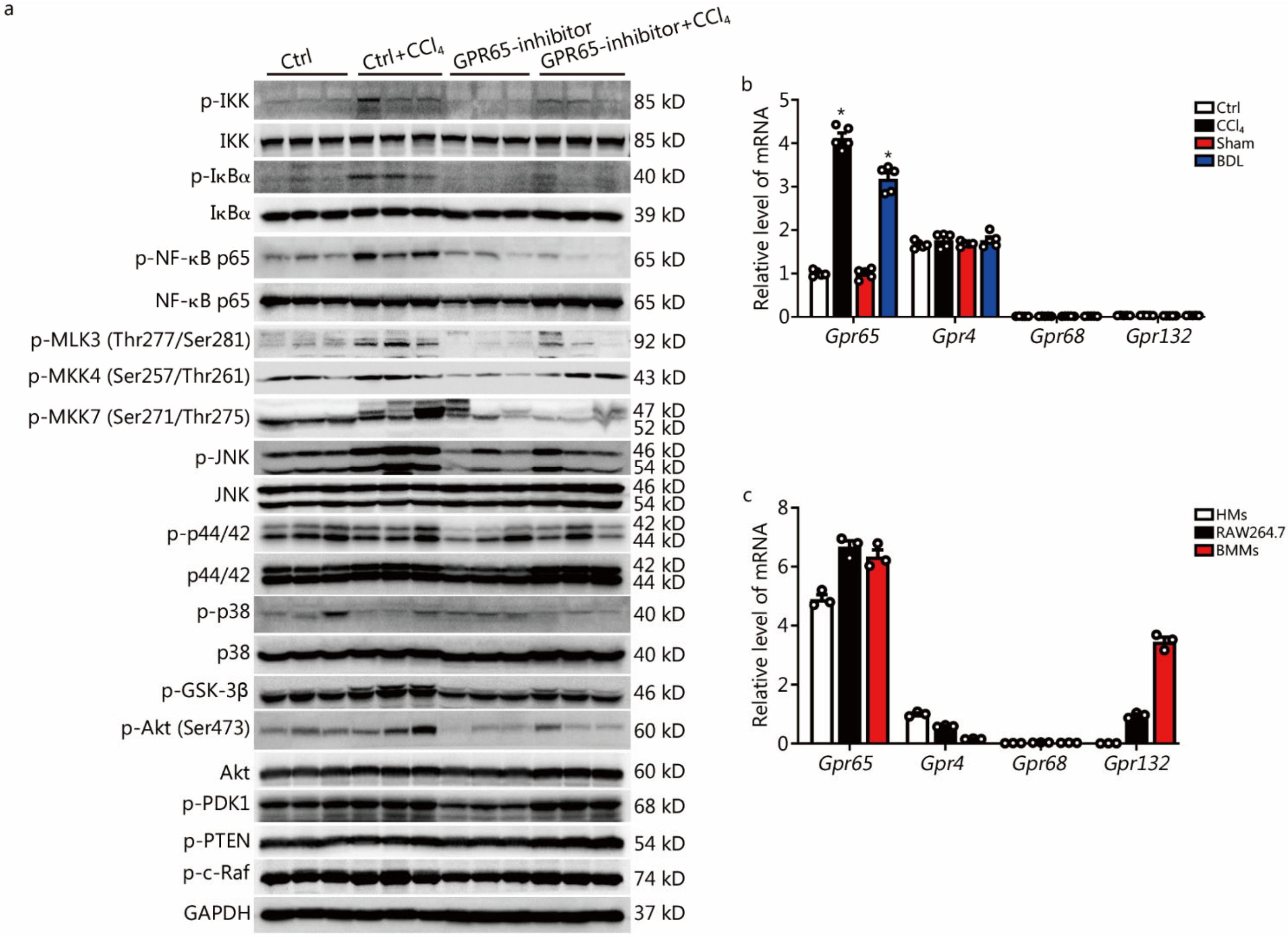

Fig. S24 Targeting GPR65 alleviates hepatic fibrosis by suppressing the JNK/NF-κB pathways, related to **Fig. 7**. **a** Western blotting was used to determine the level of p-IKK, p-IκBα, p-NF-κB p65, IKK, IκBα, NF-κB p65, p-MLK3, p-MKK4, p-MKK7, p-JNK, JNK, p-p44/42, p44/42, p-p38, p38, p-GSK-3β, p-Akt (Ser473), Akt, p-PDK1, p-PTEN and p-c-Raf in liver tissues from Ctrl, Ctrl + CCl_4, GPR65 inhibitor and GPR65 inhibitor + CCl_4 mice. **b** qRT-PCR was used to assess the expression of *Gpr65*, *Gpr4*, *Gpr68* and *Gpr132* in livers from mice treated with CCl_4 for 8 weeks or BDL for 21 d ($n = 5$). **c** qRT-PCR was used to assess the expression of *Gpr65, Gpr4, Gpr68* and *Gpr132* in HMs, RAW264.7 cells and BMMs ($n = 3$). $^{*}P < 0.05$ vs. Ctrl or Sham. BDL bile duct ligation, BMM bone marrow-derived macrophage, CCl_4 carbon tetrachloride, HM hepatic macrophage, qRT-PCR quantitative real-time reverse transcription-polymerase chain reaction, IKK inhibitor of nuclear factor κB kinase, IκBα inhibitor of κB α, IKK inhibitor of nuclear factor κB kinase, NF-κB p65 nuclear factor κB p65 subunit, MLK3 mixed lineage kinase 3, MKK4 mitogen-activated protein kinase kinase 4, MKK7 mitogen-activated protein kinase kinase 7, JNK c-Jun N-terminal kinase, GSK-3β glycogen synthase kinase-3β, Akt protein kinase B, PDK1 pyruvate dehydrogenase kinase-1, PTEN phosphatase and tensin homolog, c-Raf c-rapidly accelerated fibrosarcoma
